# Drivers of stunting reduction in Nepal: a country case study

**DOI:** 10.1093/ajcn/nqaa218

**Published:** 2020-09-05

**Authors:** Kaitlin Conway, Nadia Akseer, Raj Kumar Subedi, Samanpreet Brar, Basudev Bhattarai, Raja Ram Dhungana, Muhammad Islam, Anustha Mainali, Nikita Pradhan, Hana Tasic, Dip Narayan Thakur, Jannah Wigle, Mahesh Maskey, Zulfiqar A Bhutta

**Affiliations:** Centre for Global Child Health, Hospital for Sick Children, Toronto, Canada; Centre for Global Child Health, Hospital for Sick Children, Toronto, Canada; Dalla Lana School of Public Health, University of Toronto, Toronto, Canada; Nepal Public Health Foundation, Kathmandu, Nepal; Centre for Global Child Health, Hospital for Sick Children, Toronto, Canada; Nepal Public Health Foundation, Kathmandu, Nepal; Nepal Public Health Foundation, Kathmandu, Nepal; Centre for Global Child Health, Hospital for Sick Children, Toronto, Canada; Nepal Public Health Foundation, Kathmandu, Nepal; Nepal Public Health Foundation, Kathmandu, Nepal; Centre for Global Child Health, Hospital for Sick Children, Toronto, Canada; Nepal Public Health Foundation, Kathmandu, Nepal; Centre for Global Child Health, Hospital for Sick Children, Toronto, Canada; Dalla Lana School of Public Health, University of Toronto, Toronto, Canada; Nepal Public Health Foundation, Kathmandu, Nepal; Centre for Global Child Health, Hospital for Sick Children, Toronto, Canada; Dalla Lana School of Public Health, University of Toronto, Toronto, Canada; Center of Excellence in Women and Child Health, the Aga Khan University, Karachi, Pakistan

**Keywords:** stunting, linear growth, HAZ, children, under-5, nutrition, Nepal, South Asia, mixed methods

## Abstract

**Background:**

Chronic child malnutrition represents a serious global health concern. Over the last several decades, Nepal has seen a significant decline in linear growth stunting – a physical manifestation of chronic malnutrition – despite only modest economic growth and significant political instability.

**Objective:**

This study aimed to conduct an in-depth assessment of the determinants of stunting reduction in Nepal from 1996 to 2016, with specific attention paid to national-, community-, household-, and individual-level factors, as well as relevant nutrition-specific and -sensitive initiatives rolled out within the country.

**Methods:**

Using a mixed-methods approach, 4 types of inquiry were employed: *1*) a systematic review of published peer-reviewed and gray literature; *2*) retrospective quantitative data analyses using Demographic and Health Surveys from 1996 to 2016; *3*) a review of key nutrition-specific and -sensitive policies and programs; and *4*) retrospective qualitative data collection and analyses.

**Results:**

Mean height-for-age z-scores (HAZ) improved by 0.94 SDs from 1996 to 2016. Subnational variation and socioeconomic inequalities in stunting outcomes persisted, with the latter widening over time. Decomposition analysis for children aged under 5 y explained 90.9% of the predicted change in HAZ, with key factors including parental education (24.7%), maternal nutrition (19.3%), reduced open defecation (12.3%), maternal and newborn health care (11.5%), and economic improvement (9.0%). Key initiatives focused on decentralizing the health system and mobilizing community health workers to increase accessibility; long-standing nationwide provision of basic health interventions; targeted efforts to improve maternal and child health; and the prioritization of nutrition-sensitive initiatives by both government and donors. National and community stakeholders and mothers at village level highlighted a mixture of poverty reduction, access to health services, improved education, and increased access to water, sanitation, and hygiene as drivers of stunting reduction.

**Conclusions:**

Improvements in both nutrition-specific and nutrition-sensitive sectors have been critical to Nepal's stunting decline, particularly in the areas of poverty reduction, health, education, and sanitation.

## Introduction

Globally, stunting prevalence among children aged under 5 y has declined from 39.2% in 1990 to 21.9% in 2018. Regionally, South Asia has 1 of the highest stunting prevalence rates in the world, with 61% of children aged under 5 y stunted in 1990, down to 34% in 2018 (1).

**FIGURE 1:**
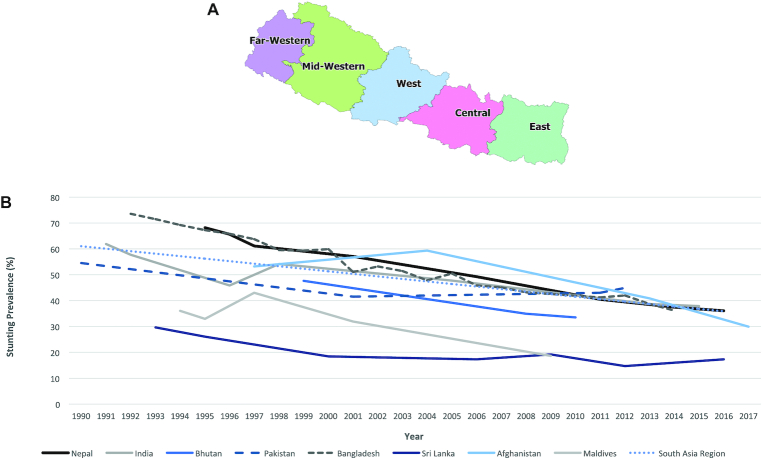
(A) Map of Nepal. (B) The prevalence of stunting in children aged under 5 y in Nepal and its neighboring countries in the South Asia Region, 1990–2018. Source: (1).

Over the last 2 decades, Nepal, a South Asian country landlocked between China and India (**Figure 1**A), has seen a sustained reduction in stunting among children aged under 5 y, especially when compared with many of the other countries in the region (Figure 1B). Between 1996 and 2016, the prevalence of stunting in Nepal was reduced from nearly 66% to 36% (2).

This achievement in addressing chronic malnutrition is especially noteworthy when considering Nepal's recent history, which includes significant political transitions and civil conflict, environmental shocks, including devastating earthquakes, and persistent poverty (3–9).

As of 2016, the population of Nepal was 29 million, made up of 125 caste/ethnic groups, with the vast majority living in rural areas (10–12). The country's terrain consists of 3 distinct ecological regions, including the flat Terai plains in the south, centrally located hills, and the Himalayan mountain range in the north.

Generally categorized as a low-income country, Nepal has seen sustained albeit modest growth in its gross domestic product (GDP), and an overall reduction in extreme poverty over the last several decades (13, 14). There has also been a sizeable increase in remittance incomes generated through labor migration, with remittances rising from 2% of GDP in 2000 to 28% in 2017 (15). Along with these improvements in socioeconomic outcomes, the country has made appreciable gains in a number of key health indicators, particularly for women and children, such as significant reductions in total and adolescent fertility rates and increases in antenatal care (ANC) visits (4+) among expectant mothers (16–18). Additional trends in key development indicators can be seen in **Supplemental Figure 1A and B** (14, 19, 20, 21–24).

Stunting is indicative of deficiencies in a child's overall environment, including their access to adequate nutrition and health care, and their exposure to repeated infections (25). Existing literature on the major drivers of Nepal's stunting decline over the last 2 decades suggest a number of important correlates, including geographic location within the country (e.g., living in the mountains or hills versus plains or in urban versus rural areas) (26–32); wealth index (5, 6, 27, 29, 31–41); maternal education (5, 6, 36, 40, 42–46); food security (35, 47–49); infant and young child feeding practices (33, 49–54); access to health services (5, 6, 29, 36, 37, 55, 56); improved sanitation (5, 6, 36, 37, 56); immunization (5, 31, 50, 57); and maternal nutrition (5, 6, 35, 40, 41, 50, 58) (**Panel A**, **Supplemental Appendix 1**). Despite the breadth of literature available on child growth outcomes in Nepal, many of these studies were subnational and/or cross-sectional in their design, focused on only a small handful of potential factors, and very few utilized a mixed-methods approach or examined the full period of stunting decline in the country from 1996 to 2016.

Panel A **Systematic literature review of stunting determinants**

Among the basic determinants of stunting in Nepal, our literature review identified living rurally and within the mountain or hill regions of the country (26–32), wealth index (5, 6, 27, 29, 31–41), and maternal education (5, 6, 36, 40, 42–46) to be significant. A total of 7 studies found the prevalence of stunting among children aged under 5 y in Nepal to be higher in rural areas and in the mountain or hill regions compared with more urban locations and the Terai plains. In particular, 1 study by Gaire et al. in 2016 found that children living in the hill region were 1.24 times as likely to be stunted as children in the Terai plains region, and children in the mountain region were 1.52 times as likely to be stunted, though only the mountain region findings were statistically significant (31). In terms of the impact of wealth index on child growth, 11 studies showed that children in the poorest households were more likely to be stunted than children in the richest households, with many of these studies showing the odds to be twice as likely or more (27, 29, 31, 32–35, 38–41). Corresponding to this, 4 studies found that asset accumulation, which can be used as a proxy for increases in wealth, was linked to improvements in HAZ among children in Nepal. However, despite these findings and the overall reduction in poverty that Nepal has experienced over the last 2 decades, 3 studies found persistent inequalities across wealth quintiles for stunting decline over the years, with larger reductions seen among the wealthy compared with the poor (28, 42, 59). Lastly, maternal education and women's empowerment, particularly as it relates to household agricultural production (26, 60, 61), were seen to have an impact on stunting outcomes in Nepal. In total, 10 studies found maternal education to have a positive impact on stunting outcomes (5, 6, 36, 40, 42–46). In particular, a 2015 study by Headey and Hoddinott found that the HAZ difference between the child of a mother with no education and 1 with 6 y of education was ∼0.17 SDs, and between a child whose mother had no education and 1 whose mother had completed secondary school was 0.34 SDs difference (6).

Underlying determinants of stunting in Nepal were food insecurity (35, 47–49), infant and young child feeding (33, 49–54), access to health services (5, 6, 29, 36, 37, 55, 56), and improved sanitation (5, 6, 36, 37, 56). A 2010 cross-sectional study conducted by Osei et al. in the Terai region found that 69% of households were food insecure and that the prevalence of stunting was slightly higher among food-insecure homes, however, this was not found to be statistically significant (35). In contrast to this, however, 3 studies included in our literature review did find living in food-deficit families to be a statistically significant risk factor for stunting in children, with 1 study by Paudel et al. in 2012 showing food insecurity to increase the odds of being stunted by 4.26 times compared with children in food-secure homes (47–49). Along with food insecurity, infant and young child feeding practices were linked to stunting in 7 studies included in the literature review (33, 49–54). Specifically, issues around inappropriate exclusive breastfeeding, complementary feeding, and a lack of dietary diversity were seen to be risk factors (49). The data compiled by Chaparro et al. in 2014 showed that the greatest increases in stunting in Nepal were taking place in infants aged between 9 and 18 mo due to inadequate breastfeeding and complementary feeding and the rise in illness and infection at this age (51). An additional 5 studies supported this finding of higher rates or worsening in the severity of stunting among older infants in Nepal (35, 43, 62, 63, 64). Access to health services, and particularly maternal and newborn health care, has been linked to a reduction in stunting in Nepal by 7 studies (5, 6, 29, 36, 37, 55, 56). A 2015 study using linear probability modeling of Nepal's DHS data from 2001 to 2011 by Headey and Hoddinott found that women attending ≥4 antenatal care visits predicted a 0.09 SD increase in HAZ, and delivery in a hospital was associated with an almost 0.20 SD improvement in HAZ (6). Lastly, improvements in sanitation were shown to have an impact on stunting outcomes in Nepal in 5 studies (5, 6, 36, 37, 56). In 1 study, community toilet use showed a 0.14 SD improvement in children's HAZ (5). Findings on the impact of access to an improved water source have been more mixed, with 2 of the 5 aforementioned studies showing tube well water sources to have a positive impact on HAZ (6, 36), whereas all 5 studies showed piped water to have no impact on HAZ (5, 6, 36, 37, 56).

The immediate determinants of stunting reduction in Nepal according to our literature review include immunization (5, 31, 50, 57) and maternal nutrition (5, 6, 35, 40, 41, 50, 58). Overall, 4 studies found links between immunization for childhood illnesses and HAZ outcomes among children in Nepal (5, 31, 50, 57). In particular, a 2016 study by Cunningham et al. found, through regression analysis of Nepal's DHS data from 1996 to 2011, that there was a relative contribution of 0.18 SDs to HAZ among children who received all of their vaccinations (5). Conversely, 2 studies included in this literature review found an association between infection and illness in Nepalese children and poor growth outcomes (64, 65). Maternal nutrition was also linked to child growth outcomes in 7 studies. Specifically, 2 studies found associations between mother's BMI and the stunting and/or length-for-age outcomes of their children, whereas 5 studies found the intergenerational impact of maternal height on child stunting outcomes to be significant, albeit to varying degrees (5, 6, 35, 40, 41, 50, 58).

Given the diversity of stunting determinants outlined in the existing body of literature and Nepal's meaningful advances in several of these areas in recent decades, it appears that there is a multifaceted success story at play in the country's achievements in reducing stunting in children aged under 5 y. Given this, our study aimed to fill existing knowledge gaps and untangle the wide array of potential drivers of stunting decline previously outlined by conducting an in-depth assessment of Nepal's stunting reduction from 1996 to 2016, with specific attention paid to national-, community/household-, and individual-level factors, as well as important nutrition-specific and -sensitive policies and programs.

The specific objectives of the study include: *1*) to quantitatively examine the determinants of stunting reduction in Nepal and to decompose long-term stunting change into the relative contribution of key drivers; *2*) to explore national- and community-level perspectives on Nepal's nutrition evolution and the major contributing factors behind stunting reduction; and *3*) to generate a systematic landscape of the major stunting-relevant policies and programs that have taken place in the country over the study period.

## Methods

### Study design

**FIGURE 2:**
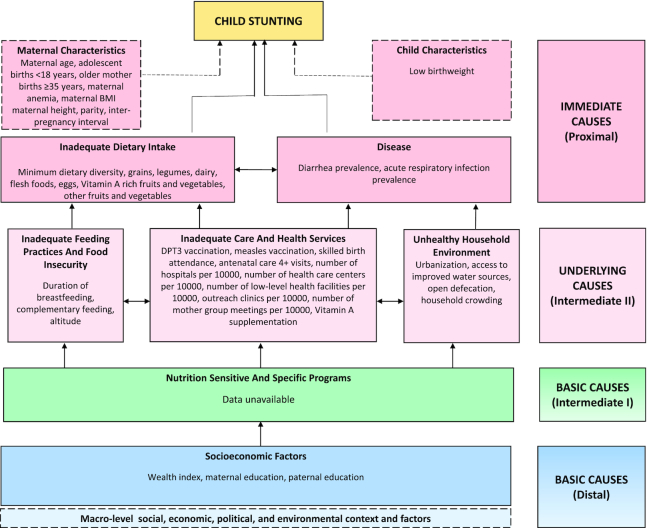
Conceptual framework showing distal, intermediate, and proximal determinants of stunting. Note: framework reflects only indicators that were measurable and available for quantitative analysis. Altitude, birthweight, and vitamin A were not available in the 2001 survey. DPT3, diptheria, pertussis, and tetanus vaccine.

This study took a mixed-methods approach, with 4 types of inquiry employed, including a systematic scoping literature review, retrospective quantitative data analyses, a comprehensive review of nutrition-specific and -sensitive policies and programs, and retrospective qualitative data collection and analyses. An adapted conceptual framework was designed for this study based on the 1995 UNICEF nutrition framework and the 2008 Lancet Maternal and Child Undernutrition series framework (66, 67) (see **Figure 2**). For the full framework used across all case studies in this project, refer to the methods article within this supplement by Akseer et al.

Ethics approval for the study, inclusive of primary data collection, was obtained from the Ethical Review Board of the Nepal Health Research Council. Ethics approval for the broader stunting case study was also obtained through the Research Ethics Board at the Hospital for Sick Children (SickKids), in Toronto, Canada. Written informed consent was obtained from the participants before primary data collection.

### Systematic literature review

Between November 2017 and July 2018, a systematic search of peer-reviewed and gray literature related to stunting of children aged under 5 y in Nepal published from 1990 to 2017 was carried out in order to understand and collate existing information on the contextual factors, national and subnational interventions, policies, strategies, programs, and initiatives that could have contributed to the decline in stunting seen in the country. Search terms employed included “stunting” or “linear growth” or “linear growth stunting” or “HAZ” or “height” or “height-for-age” or “LAZ” or “length” or “length-for-age” or “undernutrition” or “malnutrition” or “nutr*” AND “child*” or “infan*” AND “Nepal*.” Published peer-reviewed and gray literature were searched in >15 online databases and relevant gray literature sources. Of the 4604 articles initially found in this process, 109 were ultimately included in the literature review based on title/abstract and full-text screening (**Supplemental Appendix 2,Supplemental Figure 2**). A full breakdown of the systematic literature review methods, process, and detailed results can be found in Supplemental Appendix 2.

### Quantitative methods

#### Data sources

Nepal's series of Demographic and Health Surveys (DHS) (1996–2016) were the primary quantitative datasets used in this study. Available under 5 y anthropometry data by survey round are presented in **Table 1**.

**TABLE 1:** Sample size by survey year based on valid child anthropometric data^1^

	Year of DHS survey				
Age group	1996	2001	2006	2011	2016
<6 mo	564	585	440	205	216
6–23 mo	1807	1714	1398	640	686
24+ mo	755^2^	1844	1802	925	913
<36 mo	3126	3121	2625	1200	1257
<5 y	—	4143	3640	1770	1815

1 Note: based on index (youngest) child data. DHS, Demographic and Health Survey.

2 24–35 mo.

#### Outcomes and covariables

Child height-for-age z-scores (HAZ) and stunting prevalence (HAZ < –2 SDs) were the main study outcomes and were estimated using WHO child growth standards (68). Covariables were selected in line with Figure 2 as available in DHS surveys (individual/household variables) and the Nepal district health information system (DHIS) (ecological variables at district level). Potential determinants were grouped into hierarchical levels as distal, intermediate, and proximal factors to align with “basic causes,” “underlying causes,” and “immediate causes” in Figure 2.

#### Statistical analysis

We estimated child HAZ kernel density plots for all survey years to examine population shifts in growth faltering over time. Child HAZ versus age plots (“Victora curves”) were calculated using smoothed local polynomial regressions to examine the growth-faltering process from birth to age 5 y (69). Piecewise linear splines were used to estimate slopes and inflection points of Victora curve growth trajectories (70). Standardized and well-established methods for equity analyses were used to study stunting prevalence by wealth quintile (Q1–Q5), maternal education, area of residence (urban, rural), and child gender (71–73). Wealth quintiles were derived from household asset data using principal components analysis. Slope index of inequality (SII) and concentration index (CIX) accounting for cumulative distribution of the asset index were calculated to measure absolute and relative socioeconomic inequalities, respectively (71, 72). Compound annual growth rates (CAGRs) were generated to assess relative change (decline) in stunting prevalence of each region in Nepal (74).

Two sets of multivariable analyses between HAZ and various covariables were conducted using a series of stepwise linear regression models and hierarchical modeling of distal-, intermediate-, and proximal-level variables as suggested by Victora 1997 (66). The DHS 2001–2016 rounds were assembled into a panel data and difference-in-difference (DID) methods with time*covariable interaction terms used to understand whether a change in a proposed predictor of HAZ leads to a change in HAZ over the studied time period (75). The regression-based Oaxaca–Blinder decomposition methods were used to decompose change in mean child HAZ between 2001 and 2016 into its relative statistical drivers (covariables). Full method details are included in **Supplemental Appendix 3** and the methods article in this series (Akseer et al.). All analyses were conducted with Stata 14.0 (Hospital for Sick Children) and accounted for survey design and weighting.

### Policy and program review

In order to explore the impact of nutrition-specific and -sensitive policies and programs in Nepal, a timeline of key initiatives from 1990 to 2018 was assembled through an iterative process between the research team in Nepal and national expert stakeholders. Beginning with a desk review of literature identified through a systematic approach, an initial timeline was proposed by the Nepal-based research team and shared with national expert stakeholders for corroboration and further input. This process continued until consensus on the definitive nature of the timeline was reached by national experts and the Nepal research team.

### Qualitative methods

The qualitative component of this study sought to understand the drivers of stunting reduction in Nepal through the perspective of national expert stakeholders involved in the development and implementation of relevant policies and programs, and the first-hand experiences of community stakeholders and mothers at the village level. The conceptual framework presented in Figure 2 helped to inform the design of qualitative interview and focus group discussion guides and subsequent analyses. Participants were identified and selected using purposive sampling strategies, including snowball sampling (76, 77). Inclusion criteria are summarized in **Supplemental Appendix 4**(**Supplemental Table 1**).

The Thecho and Dukuchhap communities within Lalitpur district were selected for community-level data collection using convenience sampling, as they represented different geographic settings that had seen a dramatic decrease in stunting prevalence over the study period (Supplemental Appendix 4,**Supplemental Figure 3A–B**).

Qualitative data collection involved in-depth interviews with 18 national expert stakeholders and 10 community-based health workers, as well as 2 focus group discussions with 10–12 mothers in Thecho and Dukuchhap who had children born from 1995 to 2000 and 2010 to 2015, in order to understand the changes in child health and nutrition that had taken place over the study period. Data collection took place from December 2017 to May 2018. Interviews and focus group discussions were transcribed and translated into English from Nepali. Full qualitative data collection and analysis methods are detailed in Supplemental Appendix 4.

## Results

### Descriptive analyses

#### HAZ kernel density plots and Victora curves

**FIGURE 3:**
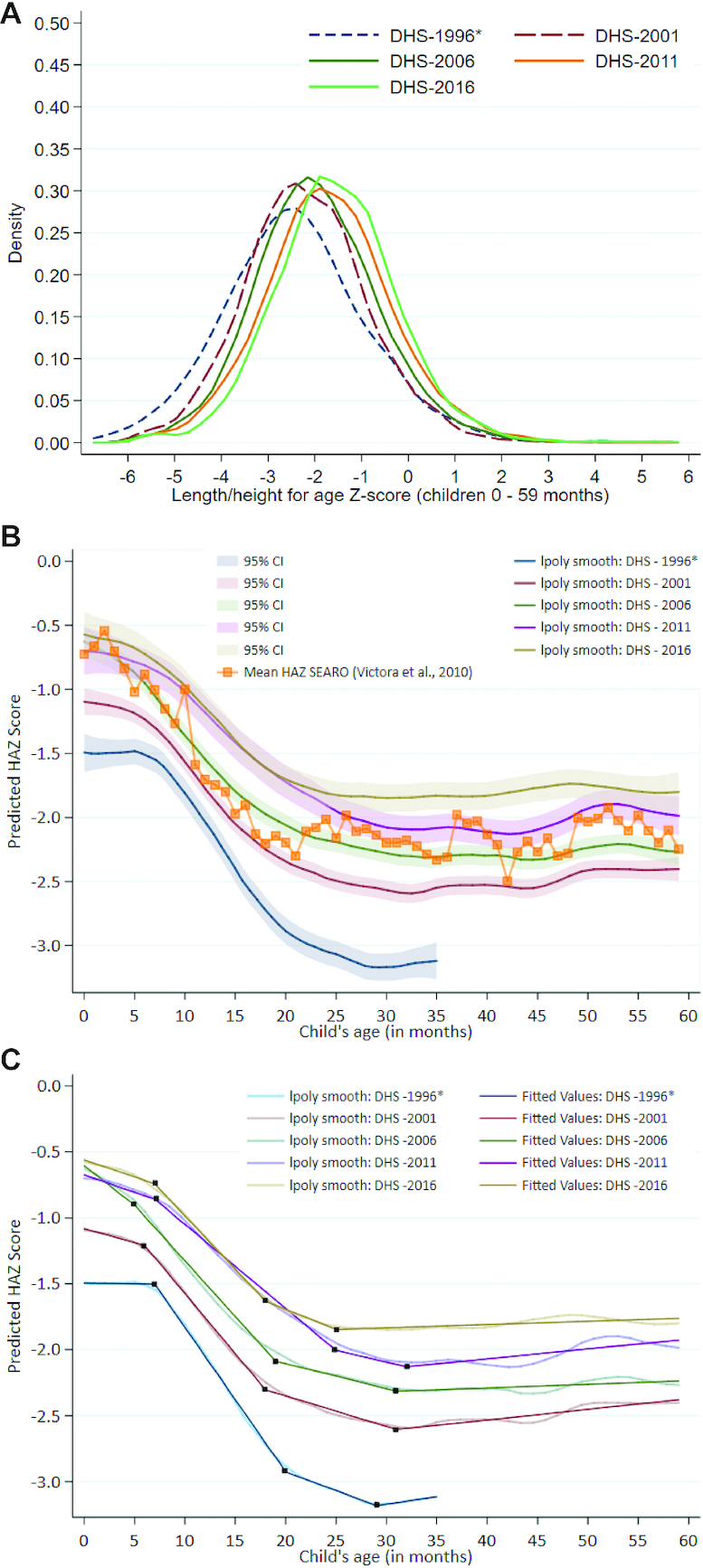
(A) Kernel density plot for HAZ distribution in children aged <5 y, 1996–2016. (B) Victora curves using data from the 1996, 2001, 2006, 2011, and 2016 DHS surveys among children aged <5 y, including SEARO mean HAZ curve. SEARO, which stands for the Regional Office for South East Asia, is 1 of the WHO's 6 regions of focus. SEARO includes Bangladesh, Bhutan, Democratic People's Republic of Korea, India, Indonesia, Maldives, Myanmar, Nepal, Sri Lanka, Thailand, and Timor-Leste. (C) Victora curves using data from the 1996, 2001, 2005, 2011, and 2016 DHS surveys among children <5 y with piecewise linear splines. *Note: DHS 1996 included <36 mo population only and thus values were adjusted to reflect the entire 24-59 mo population as described in the methods. DHS, Demographic and Health Survey; HAZ, height-for-age z-score.

**Figure 3**A outlines the HAZ distribution for children aged under 5 y in Nepal using DHS data from 1996 to 2016. The rightward shifting and narrowing of the curves over time point to nutritional gains for the entire population of children aged under 5 y over this 20 y period. The largest change occurs between 1996 and 2001, after which there are more incremental improvements in HAZ. The mean HAZ improved by 0.94 SDs over the study period, from –2.35 SD in 1996 to –1.41 SD in 2016.

Victora curves and associated piecewise linear splines are shown in Figure 3B and C; detailed coefficient estimates are in **Supplemental Appendix 5** and **Supplemental Figure 4A–E**. The greatest decline in predicted mean HAZ in 1996 was observed between children aged 7–20 mo at a rate of 0.11 SD per month (95% CI: –0.111, –0.108), reaching –2.05 SD at 12 mo. This was followed by a slower decline from 20–29 mo, falling below –3 SD at 23 mo. After 30 mo, there is a slight increase in mean HAZ, though at 35 mo it is still below –3 SD. By 2016, overall HAZ had improved significantly, to above stunted levels. Despite this, however, the relation between child age and mean HAZ is similar to that of 2001. In 2016, the steepest drop in mean HAZ occurred between 7 and 18 mo with a decline of 0.081 SD per month (95% CI: –0.084, –0.078). This is followed by a slower decline in HAZ after 18 mo, which then levels off before showing a slightly increasing HAZ from 25 to 60 mo.

#### Equity analysis

**FIGURE 4:**
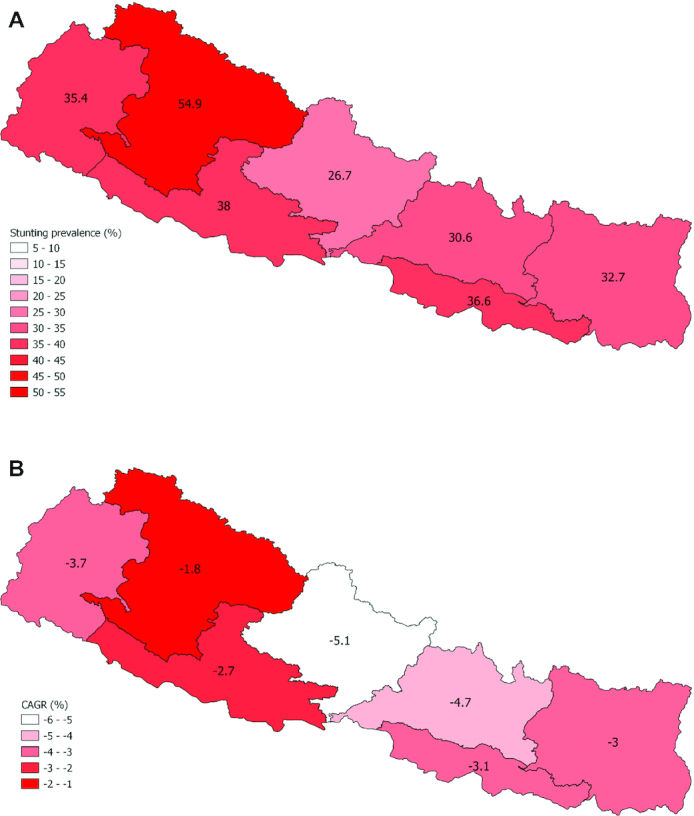
(A) Subnational stunting estimates for children aged under 5 y in Nepal in 2016. (B) CAGR by state, 2001–2016. Prior to 2015, Nepal was subnationally divided into 5 regions rather than the current 7 states. Where possible, back-calculation of subnational stunting estimates by state was done through an amalgamation of data at the district level. This was not possible for 1996 as the DHS district identifiers were not available for this year. As such, CAGR calculations are presented for the period 2001–2016. CAGR, compound annual growth rate; DHS, Demographic and Health Survey.

Despite a significant reduction in national stunting prevalence over the last 20 y, subnational disparities continue to exist within Nepal, with higher stunting prevalence rates seen in the western states (see **Figure 4**A). Stunting prevalence by province for other years is shown in Supplemental Appendix 5 and **Supplemental Figure 5A–D**.

Between 2001 and 2016, state 4 showed the largest annual reduction in stunting over this period, with a CAGR of –5.1, whereas state 6 (CAGR –1.8) saw the least change, with a CAGR of –1.85 (Figure 4B).

**FIGURE 5:**
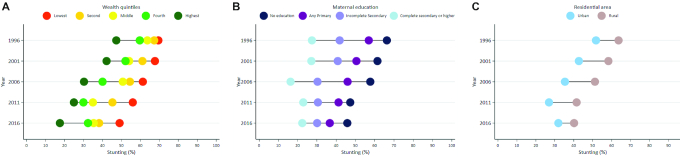
(A) Stunting prevalence in Nepal by wealth quintile, 1996–2016. (B) Stunting prevalence in Nepal by maternal education, 1996–2016. (C) Stunting prevalence in Nepal by residential area, 1996–2016.

Child stunting disparities favoring the rich were notable, with the gap between the richest and poorest widening over time (**Figure 5**A). In 1996, the difference in stunting prevalence between the wealthiest quintile (47.3%) and the poorest quintile (69.5%) was 22.2%. By 2016, this gap had increased to 32%. The widening of inequality based on wealth was also supported when analyzing the SII and the CIX (Supplemental Appendix 5, **Supplemental Figure 6A and B**). Children of mothers with greater education had lower stunting prevalence compared with those with less or no education, though the latter did experience notable stunting decline over time (Figure 5B). Disparities in stunting prevalence between those living in rural and urban areas rose between 1996 and 2011, after which they began to decline, likely tied to increases in urbanization. Despite rising urbanization, however, rural areas in Nepal have consistently higher stunting prevalence compared with urban areas (Figure 5C). Disparities across gender were minimal (Supplemental Appendix 5, **Supplemental Figure 7**).

### Multivariable analyses

**FIGURE 6:**
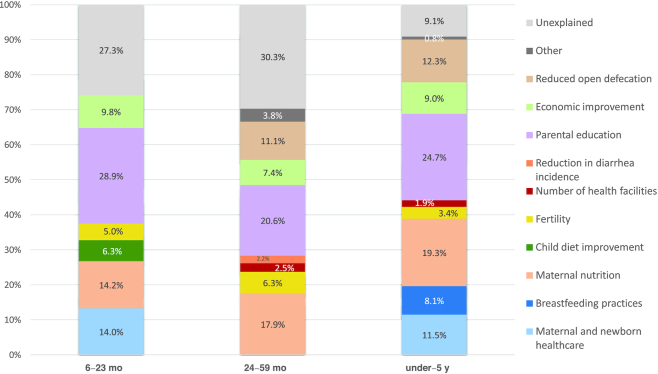
Decomposing predicted changes in HAZ (i.e., percent contribution of determinant domains) from 2001 to 2016. 1. Maternal and newborn health care (SBA and ANC 4+), maternal nutrition (maternal BMI and maternal height), fertility (number of children and pregnancy interval), reduction in diarrhea incidence, economic improvement (wealth index), other (child age, gender, and region), breastfeeding practices (duration of breastfeeding), child diet improvement (use of grains, roots, tubers, fruits, and vegetables), number of health facilities (total number of health posts or lower level health facilities per 10,000 population), parental education (maternal and paternal education), reduced open defecation. 2. Parental education breakdown: children 6–23 mo: maternal: 14.6%, paternal: 14.3%; children 24–59 mo: maternal: 13.6%, paternal: 7.1%; and children aged under 5 y: maternal: 12.2%, paternal: 12.5%. Other category includes child age, gender, and region. Note the under 6 mo age category results are not presented due to the small sample size. The 1996 DHS collected data on children aged under 3 y only and thus was not used as the starting point for analysis. ANC, antenatal care; DHS, Demographic and Health Survey; HAZ, height-for-age z-score; SBA, skilled birth attendant.

**Figure 6** illustrates child HAZ decomposition results from 2001 to 2016 for 3 age groups: children 6–23 mo (∼73% changed in HAZ explained), 24–59 mo (∼70% explained), and aged under 5 y (∼90% explained). Explanatory factors have been color-coded and their contribution to predicted changes in HAZ are shown along the *y*-axis (see Supplemental Appendix 5, **Supplemental Tables 2–5** and **Supplemental Figures 8**–**10** for supportive analyses). Improvement in parental education comes across as the strongest predictor for all age groups, ranging from a 20% to nearly 30% contribution, followed by maternal nutrition with a 14% to nearly 20% contribution. Economic improvement was also notable across age groups, with a 7–10% contribution. Improvements in maternal and newborn care and reduced open defecation also contribute meaningfully to ∼11–14% change across age groups. Child dietary improvement was meaningful in the 6–23 mo age group where data was available for analysis (6.3%).

The DID analyses (Supplemental Appendix 5, **Supplemental Tables 6–9**) showed that, across age groups, increased piped water access, lower diarrhea incidence, greater number of health facilities, and having skilled attendants at birth each contributed to HAZ change between 2001 and 2016.

### Policy and program review

**FIGURE 7:**
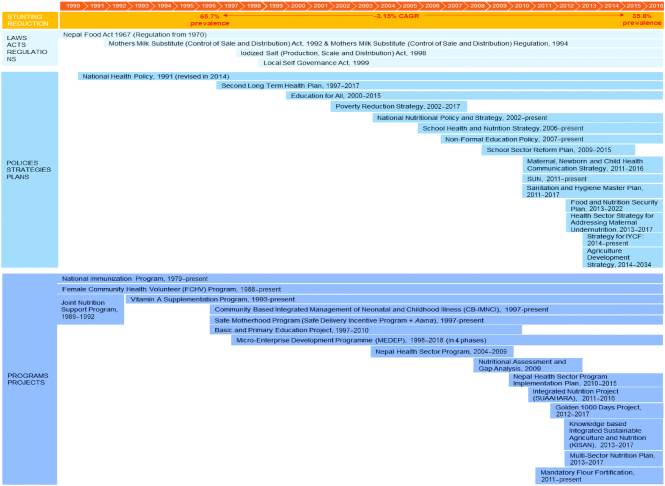
Overview of laws, policies, programs, and enablers between 1990 and 2018 in Nepal. CAGR, compound annual growth rate. IYCF, Infant and Young Child Feeding; SUAAHARA, 'Good Nutrition,' a USAID/Nepal project; SUN, Scaling Up Nutrition.

A timeline of key nutrition-specific and -sensitive programs and policies adopted in Nepal is shown in **Figure 7** and **Panel B** (4, 78–83). For several decades, Nepal has established long-term visions and plans for broad national development, as well as targeted policies and programs to address health challenges and inequities. Investments in health system strengthening by the government and external development partners involved the establishment of a cadre of female community health volunteers (FCHVs), dramatic expansion of the health facility infrastructure, substantial investments in public health services [e.g., immunization, vitamin A supplementation, and Community-based Integrated Management of Neonatal and Childhood Illness (CB-IMNCI)], as well as the establishment of an essential health care service (EHCS). Long-term health plans and a series of health sector reforms (e.g., National Health Policy, Nepal Health Sector Program (NHSP)-Implementation Plan, NHSP-II, and the Nepal Health Sector Support Programme) have had ongoing political commitment to improving population health in Nepal. Investments in nutrition-sensitive sectors including education/literacy, propoor economic growth/poverty reduction, and locally driven efforts to improve water, sanitation, and hygiene (WASH) have also been key. Expanded information on each initiative is in **Supplemental Appendix 6** and **Supplemental Table 10**.

Panel B **Program and Policy Review**

Following the establishment of a constitutional monarchy and multiparty democracy in 1990, the government of Nepal launched several initiatives to reform and strengthen health care delivery. Chief among these was the new vision for the health system via the 1991 National Health Policy, which focused on providing essential care services to the country's largely rural population in a more accessible way. Along with this came the Second Long Term Health Plan (1997–2017), which built on the First Long Term Health Plan's efforts to improve health service delivery by working to address disparities in health outcomes and making quality health care more readily available at the community level.

In 2007, Nepal's Interim Constitution stated that every citizen had the right to free basic health services provided by the state. Since then, free essential health services have been progressively rolled out, reaching a national scale in 2009. All citizens are now eligible to access district hospitals, primary health care centers, health posts, and subhealth posts without paying a registration fee. They can also access free outpatient, in-patient, and emergency services, and free medication from a list of essential drugs (9, 78). Despite the government commitment to health as a human right and an observed increase in service utilization, challenges to the implementation of free essential health services have been ongoing in Nepal. Among these are issues surrounding proximity and distribution of health services across the country, inadequate budgeting for health, drug stock outs, and a lack of sufficient human resources for health, particularly in remote and rural areas (78,80).

These initiatives also came alongside long-standing commitments by both the government and external donors to lifesaving health interventions such as the National Immunization Program (1979–present) and the National Vitamin A Supplementation Program, as well as the development of new packages of interventions such as the CB-IMNCI (1997–present), and the National Nutritional Policy and Strategy (2004–present).

In order to achieve its goal of greater access within the health system and the broader provision of essential services, the government began a push in the 1990s for decentralization through 2 key initiatives. Firstly, the FCHV Program helped extend the reach of the health system through the training of married women between the ages of 25 and 45 y to provide health and nutrition information and basic health services within their communities. When it was rolled out nationally in 1992, the FCHV Program had ∼20,000 volunteers. According to the most recent estimates, there are currently >50,000 volunteers working across Nepal (81). Secondly, the Local Self Governance Act of 1999 created the legal framework for important decisions to be made at the community level, including those related to health and education.

Along with a focus on basic health services, the government of Nepal and its development partners placed special focus on improving maternal and newborn health outcomes through overarching programs such as the Safe Motherhood Program (1997–present) (82). From this initiative, subsequent efforts such as the 2005 Safe Delivery Incentive Program (later becoming *Aama*, the Nepali word for “mother”) and the 2006 National Policy on Skilled Birth Attendants were created. While the former works to provide free delivery services and cash incentives to both the institutions providing maternal care and the women accessing it, the latter has worked to drive-up the number of skilled birth attendants available to assist in delivery (4, 83).

The prioritization of key nutrition-sensitive programs by both government and donors was also noteworthy. Among these include long-term efforts to improve education and literacy through the Education for All Initiative (2000–2015); a focus on propoor economic growth and increased access to social and economic resources for the marginalized through the Poverty Reduction Strategy (2002–2007); and locally driven efforts to improve the WASH sector via Community-Led Total Sanitation (CLTS) beginning in 2000.

### Qualitative inquiry results

Across stakeholders consulted, there was general consensus that child stunting reduction was related to key drivers including improved education of women and girls, greater financial and social empowerment of households and mothers, poverty reduction, improved living conditions, reduction in open defecation, less child illness, improved dietary intake/diversity (including better breastfeeding practices), reduced adolescent births, and lower fertility/increased birth spacing. For the full qualitative results, see **Panel C** and **Supplemental Appendix 7, Supplemental Tables 11–12**.

Panel C **Qualitative inquiry results**

**National expert stakeholders**

Among the contextual factors for stunting decline that national expert stakeholders pointed to were the overcoming of political instability and conflict, improvements in education (particularly for women and girls), women's empowerment, increases in remittances allowing for improvements in the standard of living and nutrition, reduction in poverty, and urbanization.

Nutrition-specific and -sensitive policies and programs discussed during interviews with national expert stakeholders were ranked based on the number of times they were mentioned or endorsed by the participants. Overall, what was most frequently referenced was the importance of a broad mixture of initiatives that target both specific outcomes such as maternal and newborn health and nutrition, and more broad-reaching efforts, such as those focused on WASH, poverty reduction, and education.

Among the underlying causes of stunting reduction, national expert stakeholders pointed to improvements in WASH, an increase in the accessibility of essential health services at the community level, and critical improvements in food security and overall dietary diversity due to changing agricultural practices.

“WASH-related policies were also quite useful in reducing infection. Campaigns on ODF (Open Defecation Free) were instrumental in reducing infections among children, thereby improving nutritional status including stunting.” - Representative from the Ministry of Health and Population (former).

Among the immediate causes of stunting reduction, expert stakeholders outlined a reduction in infections and communicable diseases, increased dietary intake (particularly of macro- and micronutrients and improved infant and young child feeding practices), and decreased fertility, especially among the adolescent population.

**Community stakeholders**

Community stakeholders, represented by FCHVs and other community health workers, described increases in remittance incomes and expanded access to health services as essential to improvements in child health outcomes. Importantly, the labor migration of men within communities and increased education among women were seen to have knock-on effects for women's empowerment, with more women assuming roles as the head of the household and as decision-makers.

“...Education, especially among women, has improved over the years and has led to self-understanding about the availability and utilization of health services. Women's education is a proven and important means of achieving gender equality, the effects of which are felt throughout families and communities if compared with the past.” - Community Health Worker, Thecho.

Related to nutrition-specific and -sensitive policies and programs, community health workers outlined the importance of increased donor and development partner funding leading to an expansion of health service providers, which led to improvements in the ability to address maternal, newborn, and child health, as well as nutrition.

For underlying causes, many community health workers described increased access to health services and WASH improvements over the study period. For immediate causes, participants among the health workers also pointed to a reduction in child illness and improved dietary intake, including exclusive breastfeeding.

**Mothers in communities**

Among the contextual factors outlined by mothers were improvements in socioeconomic status and a reduction in poverty as well as improvements in education and the empowerment of women to make decisions within their families and communities.

“…compared to early days, it has improved a lot. We had nothing. We were very much poor. The major source of income was only agriculture. We struggled for minimal day-to-day survival. But now the times have changed. We are now economically strong. Our sons/daughters are abroad. They send us money. We do have our own agricultural land. We consume food for ourselves from there and also sell seasonally.” (Smiling) - P1: mother of a child born between 1995–2000.

Among the underlying factors for stunting reduction, mothers pointed to improvements in food security, water and sanitation, and overall access to health services. In particular, women pointed out how the conflict from 1996 to 2006 and issues of geographic remoteness had an impact on the availability of food. They also recognized how demands on their time had changed over the last 20 y, allowing more attention to be paid to infant and young child feeding, and particularly to breastfeeding. In terms of health services, local access via community health workers and improvement of government and private health facilities was seen to be important. Several participants also pointed to improvements in roads and transportation services, though many noted progress is still needed in this area.

“The health facilities in the past used to have no medical equipment and professional personnel used to be very far, which is not the case today. Now the transports reach to the door of health institutions. Everything has improved with time. Availability of drugs and other services is noted to be continually provided to every sick patient with no age bar” - P5: mother of child born between 1995–2000.

Among the immediate causes of stunting reduction mentioned by mothers in the community were improved dietary diversity and overall dietary intake, and decreased incidence of infectious disease, low birth weight, and fertility rates, as well as greater spacing between births.

## Discussion

### Summary

Our mixed-methods assessment of the determinants of reducing the stunting in children aged under 5 y in Nepal over the last 2 decades supports the multifactorial nature of this country's remarkable achievement. Investments in both nutrition-specific and -sensitive sectors have been critical, particularly in the areas of poverty reduction, health, education, and sanitation.

Government policies and donor initiatives coupled with a grassroots community-based health system and availability of remittances appears to have allowed for the driving down of poverty as well as greater dietary diversity, food security, and health-seeking behavior. Both the government and its donor partners showed a commitment to improving maternal, newborn, and child health through long-standing health programming and the delivery of basic health services at the community level via FCHVs. Improvements in education and literacy for all (including children, fathers, and mothers), but especially for women and girls, as well as significant improvements to WASH access through community-driven initiatives allowed greater female empowerment and safer environments for children to grow with a lowered risk of infection, respectively.

Running through these key areas of improvement are the recurring themes of a focus on the poor and marginalized by both the government and its partners, strong donor collaboration in achieving development outcomes, and an overall push towards decentralization in order to meet the needs of the country's largely rural-based population.

### Strengths and limitations

There are 5 key strengths to this study. First, to our knowledge, this is the first mixed-methods, comprehensive overview of the drivers of stunting reduction in Nepal utilizing a 4-pronged approach of a systematic literature review, quantitative analyses of national-level data, qualitative collection and analyses of both national and subnational data, and review of nutrition-specific and -sensitive initiatives within the country. Second, it includes an expansion of the range of determinants of stunting reduction than has been previously examined, achieved through the use of our evidence-based conceptual framework (for instance, dietary intake markers for children aged 6–23 mo, older and adolescent maternal age, incidence of diarrhea and acute respiratory infection, and maternal anemia among others), harmonization of individual-level DHS data from 1996 to 2016, and consideration of district-level ecological variables from reliable sources. Third, by using a hierarchical model in our analyses, we were better able to explore the pathways of the determinants of stunting. Fourth, by incorporating both national- and community-level perspectives in our qualitative analyses, we were able to capture a diverse range of viewpoints and a more complete picture of the nutritional transition for children in Nepal. Lastly, through our policy and program analysis, vetted by national expert stakeholders, we provide a comprehensive timeline of key initiatives in the country that could have impacted nutritional outcomes.

There were also several limitations met within this study. First, in terms of the qualitative data collection, due to logistical and time constraints, focus group discussions with mothers were only conducted in 1 district, when sampling of mothers from multiple districts around the country would have provided a more accurate representation. Second, in terms of our ecological variables, limitations may exist where regional data may not actually translate to individual-level effects. Third, though adjustments were made for confounding variables, confounding may still arise due to unmeasured confounders or variables with poor estimations. Fourth, there is also the potential for limitations within the application of Oaxaca–Blinder decomposition, as has been outlined in existing literature (36, 37, 84). Lastly in terms of data, in general, direct measures for a number of variables such as those related to food insecurity and dietary intake for children aged under 5 y were lacking, necessitating the use of proxy variables when possible.

### Existing evidence

An examination of the results provides a multifaceted story combining contextual factors with targeted policy and program initiatives and the amelioration of many of the underlying and immediate causes of child malnutrition within the country.

Despite only moderate economic growth and a prolonged period of political instability and conflict, Nepal was able to reduce stunting dramatically over the last 20 y, from a 66% prevalence in 1996 to 36% in 2016. Interestingly, in contrast to many other countries who have experienced civil and political unrest, Nepal was able to maintain improvements in many indicators throughout years of conflict, particularly in relation to improvements in human development and maternal, newborn, and child health (8, 85). Although evidence on how these improvements continued to be achieved is limited, existing literature describes the relatively small impact of the conflict on long-standing health services such as micronutrient distribution and immunization coverage, as well as the pressure placed on combatants to allow health services to continue as possible explanations (85).

Our results point to several key drivers of stunting decline in the country including, firstly, poverty reduction and an increase in food security. Poverty reduction and wealth accumulation were seen as determinants for improvements in stunting outcomes in Nepal across both our quantitative and qualitative findings, and are supported widely in the existing literature (5, 6, 27, 29, 31–41). Beyond remittances, some literature has also pointed to the role of growth in the agriculture sector (3.5% per year between 2001 and 2011) in reducing poverty in Nepal, however, the gains made in this area were not shown through our findings to be large enough to have significantly impacted stunting (5). Overall, low productivity and a lack of competitiveness in the agriculture market have continued to present challenges to significant progress in poverty reduction and overall food security being attributed to agricultural growth in Nepal.

Secondly, since the 1980s, there has also been a commitment among government and development partners to support long-standing national health programs such as the National Immunization Program, the Vitamin A Supplementation Program, the Safe Motherhood Program, Community-based Integrated Management of Neonatal and Childhood Illness (CB-IMNCI), and the National Nutrition Policy and Strategy. Along with this, there have been significant efforts to reform the health system and commit to the delivery of essential health services through efforts such as the 1991 National Health Policy and the Second Long Term Health Plan. These 2 initiatives acted as frameworks from which there has been a notable improvement in accessibility within the health system, furthered by efforts such as the launching of the FCHV Program and the Local Self Governance Act.

Existing literature supports the impact of increased access and utilization of health services on child growth outcomes in Nepal over the last several decades (5, 86), and particularly as they relate to increased immunization (5, 31, 50, 57) and the provision of ANC (29, 6, 37, 55). Although the literature has been mixed on the impact of vitamin A on child growth outcomes in Nepal (87), throughout our study, the country's long-standing national Vitamin A Supplementation Program came forth as an important indicator of both a child's overall engagement with the health system and the government's commitment to nutrition-specific initiatives. Improvements in maternal nutrition markers such as BMI (76), nutritional intake such as vegetables (88), and maternal height (89, 32, 35, 36, 39) have also shown strong associations to child undernutrition in several earlier studies in Nepal.

Thirdly, Nepal's successful investments in education for all have had a notable impact on health and nutrition. Father's education had a significant impact on improving child HAZ, and mechanisms of impact could be linked to better overall understanding of child and family health care needs, healthier choices in food, better employment prospects and higher household income, and greater overall importance given to the education of women and girls, along with support and empowerment in the household. Improved mother's education was also an important driver of improved child HAZ in Nepal. Along with a strengthening of reproductive and maternal health services, increased education and empowerment have helped to lower fertility rates among both women and adolescents and impacted rates of early marriage, all of which can be tied to improved child nutritional outcomes. The impacts of both education and women's empowerment, as well as reproductive and maternal health outcomes on HAZ and stunting in Nepal have been widely supported in the literature (5, 6, 29, 35–37, 40, 41, 42–46, 55, 58, 88, 60, 90–92,60, 90–92). Nepal's efforts in improving health service reach and access through extension workers, greater access to ANC services, improved prenatal supplementation, and higher female education may be key reasons for greater child birth size observed between 1996 and 2006; the plateauing of birth size thereafter could be due to poor quality of care and pervasive subnational inequalities that preclude national-level improvement.

Fourthly, the results of both our quantitative and qualitative analyses show the importance of community-driven initiatives around WASH. These have led to significant improvements in access to clean water and a reduction of open defecation that have significant implications for lowering infection, disease, and poor nutritional outcomes in children. Existing literature on the impact of WASH interventions is mixed, with the WASH Benefits trials in Kenya and Bangladesh and the Sanitation Hygiene Infant Nutrition Efficacy (SHINE) trials in Zimbabwe showing WASH interventions alone to have no effect on reducing stunting in low-income country settings (93–96). In contrast to these aforementioned 2 y trials, however, our findings are representative of the impact of WASH interventions over a 20 y period, and may provide a more holistic assessment of the benefits of improved WASH on child growth. Existing literature specific to Nepal supports our findings related to the positive impact of WASH interventions on linear growth outcomes, with a study by Cunningham et al. in 2016 using linear regression modeling to show community toilet use having an impact of 0.14 SD on HAZ outcomes for children aged under 5 y (5).

Another driver relates to donor coordination and a commitment to multisectoral initiatives for improving nutrition. This has set into motion a number of recent policies and programs whose potential impact for further stunting decline are a promising way forward for Nepal, and provide a strong example for other countries.

### Remaining challenges and future research

Despite Nepal's dramatic reduction in stunting between 1996 and 2016, the national prevalence remains high at 36%, or more than one-third of children aged under 5 y with stunted growth. Along with this, subnational disparities persist, particularly related to wealth and maternal education, and in the case of wealth, these disparities are increasing over time. Further reduction in stunting prevalence will require targeted action towards reaching these disadvantaged populations. Progress in child stunting since 2011 has slowed and this could be due to a multitude of factors including an overburdened health extension worker system, changing government priorities, new challenges in program implementation, among other factors; future work should investigate bottlenecks and solutions to continue progress. Our study revealed important insights related to child growth-faltering trajectories for specific age groups over time that warrant further study to understand the mechanisms of change.

Additionally, despite progress made in stunting, signs of other nutritional deficiencies in Nepal remain a cause for concern. For example, underweight among children aged under 5 y remains quite high at 27%, and the rate of wasting has gone largely unchanged over our study period, currently resting at 10% (97, 98). Maternal anemia also continues to pose a significant health risk within the country, with 40% of pregnant women experiencing anemia as of 2016 (99).

Further research is required in order to examine and test this article's findings on the subnational drivers of stunting reduction in Nepal. For instance, whereas several states have done well, state 6 has clearly lagged behind in stunting progress; additional research should investigate the challenges and barriers experienced by this state particularly as it may pertain to socioeconomic factors and relevant nutrition policies and programs. Additionally, research that examines the determinants of other child and maternal nutritional outcomes alongside stunting could shed light on areas where further gains can be made. Beyond this, cost-effective analyses of various programs and policies outlined throughout this article could allow prioritizing of future funding as well as crosscountry learning opportunities.

## Conclusion

Though there is no silver bullet responsible for the decline in stunting seen in Nepal over the last 20 y, when all of the above factors are combined together, a picture emerges of a multipronged approach centered on poverty reduction, health care access, education, and sanitation that has allowed Nepal to make laudable progress in combating chronic child malnutrition.

## Supplementary Material

nqaa218_Supplemental_Files

## Notes

Presented at the conference, World Health Assembly held in Geneva, Switzerland, 17–21 May, 2020.

This study was funded by a grant to the Centre for Global Child Health from Gates Ventures.

Data described in the manuscript, code book, and analytic code will be made available upon request pending application and approval.

Published in a supplement to the *American Journal of Clinical Nutrition*. The Guest Editor for this supplement was Mark Manary and reports no conflicts of interest. The publication costs for this supplement were defrayed in part by the payment of page charges by Gates Ventures. The opinions expressed in this publication are those of the authors and are not attributable to the sponsors or the publisher, Editor, or Editorial Board of the *American Journal of Clinical Nutrition*.

The supplement coordinator for the supplement publication was NA, Gates Ventures/Hospital for Sick Children, Toronto, Canada. The supplement coordinator reports no conflicts of interest. The Stunting Exemplars research principal investigator was ZAB, Hospital for Sick Children, Toronto, Canada. The principal investigator reports no conflicts of interest.

Supplemental Tables 1–12, Supplemental Figures 1–10, and Supplemental Appendices 1–7 are available from the “Supplementary data” link in the online posting of the article and from the same link in the online table of contents at https://academic.oup.com/ajcn/.

SB and JW, authors listed alphabetically.

Abbreviations used: ANC, antenatal care; CAGR, compound annual growth rate; CB-IMNCI, Community-based Integrated Management of Neonatal and Childhood Illness; CIX, concentration index; DHS, Demographic and Health Survey; DID, difference-in-difference; FCHV, female community health volunteer; GDP, gross domestic product; HAZ, height-for-age z-scores; NHSP, Nepal Health Sector Program; SII, slope index of inequality; WASH, water, sanitation, and hygiene.

## References

[1] WHO WHO | Joint Child Malnutrition Estimates - Levels and Trends(2019 edition) [Internet] WHO. World Health Organization; 2019; [cited 4 Jun, 2019]. Available from: https://www.who.int/nutgrowthdb/estimates2018/en/.

[2] The World Bank Prevalence of Stunting, Height for Age (% of Children Under 5) | Data. [Internet] 2019; [cited 7 Mar, 2019]. Available from: https://data.worldbank.org/indicator/SH.STA.STNT.ZS?locations=NP.

[3] Reuters TIMELINE: Main events in Nepal's Maoist war and march to peace | Reuters. [Internet] 2008; [cited 19 Oct, 2018]. Available from: https://www.reuters.com/article/us-nepal-elections-maoists/timeline-main-events-in-nepals-maoist-war-and-march-to-peace-idUSDEL23817820080414.

[4] Ministry of Health and Population Nepal, Partnership for Maternal, Newborn & Child Health, WHO WB and A for HP and SR Success Factors for Women's and Children's Health, NEPAL. [Internet] Geneva: Bulletin of the World Health Organization; 2014 Available from: http://www.who.int/pmnch/knowledge/publications/nepal_country_report.pdf.

[5] Cunningham K, Headey D, Singh A, Karmacharya C, Rana PP Maternal and child nutrition in Nepal: examining drivers of progress from the mid-1990s to 2010s. Glob Food Sec. 2017;; 13;30–7.

[6] Headey DD, Hoddinott J. Understanding the rapid reduction of undernutrition in Nepal, 2001–2011. PLoS One. 2015;10:e0145738. 26699135 10.1371/journal.pone.0145738 PMC4690594

[7] UNICEF Situation of Children and Women in Nepal. [Internet] Kathmandu, Nepal; 2006 Available from: https://www.unicef.org/Nepal_SitAn_2006.pdf.

[8] Partap U, Hill DR The Maoist insurgency (1996–2006) and child health indicators in Nepal. Int Health. 2012;4:135–42. 24029152 10.1016/j.inhe.2011.12.004

[9] Rafferty JP Nepal Earthquake of 2015 | Magnitude, Death Toll, Aftermath, & Facts | Britannica.com. [Internet] 2018; [cited 19 Oct, 2018]. Available from: https://www.britannica.com/topic/Nepal-earthquake-of-2015.

[10] The World Bank Population, total | Data. [Internet] 2019; [cited 19 Feb, 2019]. Available from: https://data.worldbank.org/indicator/SP.POP.TOTL?locations=NP.

[11] Central Bureau of Statistics National Population and Housing Census 2011 (National Report). [Internet] 2012 Available from: http://cbs.gov.np/image/data/Population/National Report/National Report.pdf.

[12] UNFPA Nepal Population Situation Analysis of Nepal (With Respect to Sustainable Development). [Internet] 2017 Available from: https://www.unfpa.org/.

[13] The World Bank GDP (current US$) | Data. [Internet] Open Data. 2018; [cited 21 Aug, 2018]. Available from: https://data.worldbank.org/indicator/NY.GDP.MKTP.CD?locations=NP.

[14] The World Bank Poverty Headcount Ratio at $1.90 a Day (2011 PPP) (% of Population) | Data. [Internet] 2019; [cited 19 Feb, 2019]. Available from: https://data.worldbank.org/indicator/SI.POV.DDAY?locations=NP.

[15] The World Bank Personal Remittances, Received (% of GDP) | Data. [Internet] 2019; [cited 18 Mar, 2019]. Available from: https://data.worldbank.org/indicator/BX.TRF.PWKR.DT.GD.ZS?locations=NP.

[16] The World Bank Fertility Rate, Total (Births Per Woman) | Data. [Internet]. Open Data 2018; [cited 4 Sep, 2018]. Available from: https://data.worldbank.org/indicator/SP.DYN.TFRT.IN?locations=NP.

[17] The World Bank Adolescent Fertility Rate (births per 1,000 women ages 15-19) | Data. [Internet] 2019; [cited 11 Mar, 2019]. Available from: https://data.worldbank.org/indicator/SP.ADO.TFRT?locations=NP.

[18] USAID Antenatal visits for pregnancy: 4+ visits [Internet]. STATcompiler: The DHS Program. 2019 [cited 25 Feb, 2019]. Available from: https://www.statcompiler.com/en/.

[19] The World Bank GDP per capita, PPP (constant 2011 international $). [Internet] Open Data. 2018 Available from: https://data.worldbank.org/indicator/NY.GDP.PCAP.PP.CD?locations=NP.

[20] The World Bank People Practicing Open Defecation (% of Population) | Data. [Internet] 2019; [cited 25 Feb, 2019]. Available from: https://data.worldbank.org/indicator/SH.STA.ODFC.ZS?locations=NP.

[21] The World Bank Urban Population (% of Total) | Data. [Internet] 2019; [cited 7 Mar, 2019]. Available from: https://data.worldbank.org/indicator/SP.URB.TOL.IN.ZS?locations=NP.

[22] The World Bank Literacy rate. , adult total (% of people ages 15 and above) | Data. [Internet] Open Data. 2018; [cited 6 Sep, 2018]. Available from: https://data.worldbank.org/indicator/SE.ADT.LITR.ZS?locations=NP&view=chart.

[23] The World Bank Literacy Rate, Adult Female (% of Females Ages 15 and Above) | Data. [Internet] 2019; [cited 25 Feb, 2019]. Available from: https://data.worldbank.org/indicator/SE.ADT.LITR.FE.ZS?locations=NP.

[24] UNDP Human Development Reports: Gender Development Index (GDI). [Internet] 2018; [cited 25 Feb, 2019]. Available from: http://hdr.undp.org/en/indicators/137906#.

[25] Leroy JL, Frongillo EA Perspective: what does stunting really mean? A critical review of the evidence. Adv Nutr. 2019;10:196–204. 30801614 10.1093/advances/nmy101 PMC6416038

[26] Shively G, Gars J, Sununtnasuk C A Review of Food Security and Human Nutrition Issues in Nepal. [Internet] West Lafayette, Indiana; 2011 Available from: http://ageconsearch.umn.edu/bitstream/116190/2/11-5.pdf.

[27] Devkota MD, Adhikari RK, Upreti SR Stunting in Nepal: looking back, looking ahead. Matern Child Nutr. 2016;12:257–9. 27187924 10.1111/mcn.12286 PMC5084730

[28] Gurung G. Child health status of Nepal: social exclusion perspective. J Nepal Paediatr Soc. 2009;29:79–84.

[29] Nisar YB, Dibley MJ, Aguayo VM Iron-folic acid supplementation during pregnancy reduces the risk of stunting in children less than 2 years of age: a retrospective cohort study from Nepal. Nutrients. 2016;8:67. 26828515 10.3390/nu8020067 PMC4772031

[30] Thapa M, Neopane AK, Singh UK, Aryal N, Agrawal K, Shrestha B Nutritional status of children in two districts of the mountain region of Nepal. J Nepal Health Res Counc. 2013;11:235–9. 24908522

[31] Gaire S, Delbiso TD, Pandey S, Guha-Sapir D Impact of disasters on child stunting in Nepal. Risk Manag Healthc Policy. 2016;9:113–27. 27354834 10.2147/RMHP.S101124 PMC4908949

[32] Shively G, Sununtnasuk C Agricultural diversity and child stunting in Nepal. (Special Issue: farm-level pathways to improved nutritional status.). J Dev Stud. 2015;51:1078–96.

[33] Tiwari R, Ausman LM, Agho KE Determinants of stunting and severe stunting among under-fives: evidence from the 2011 Nepal Demographic and Health Survey. BMC Pediatr. 2014;14:239. 25262003 10.1186/1471-2431-14-239 PMC4263111

[34] Devakumar D, Kular D, Shrestha BP, Grijalva-Eternod C, Daniel RM, Saville NM, Manandhar DS, Costello A, Osrin D, Wells JCK Socioeconomic determinants of growth in a longitudinal study in Nepal. Matern Child Nutr. 2018;14:e12462. 10.1111/mcn.12462 PMC5763270 28449415

[35] Osei A, Pandey P, Spiro D, Nielson J, Shrestha R, Talukder Z, Quinn V, Haselow N Household food insecurity and nutritional status of children aged 6 to 23 months in Kailali District of Nepal. Food Nutr Bull. 2010;31:483–94.

[36] Headey D, Hoddinott J, Park S Drivers of nutritional change in four South Asian countries: a dynamic observational analysis. Matern Child Nutr. 2016;12:210–8. 27187917 10.1111/mcn.12274 PMC5084796

[37] Headey D, Hoddinott J, Park S Accounting for nutritional changes in six success stories: a regression-decomposition approach. Glob Food Sec. 2017;13:12–20.

[38] Niraula SR, Barnwal SP, Paudel S, Mishra S, Dahal S, Das S, Pradhan S, Ghimire S, Khanal S, Sharma S et al. Prevalence and associated risk factors with malnutrition among under-five Nepalese children of Borbote village, Ilam. Heal Renaiss. 2013;11:111–8.

[39] Pradhan A Fitting ordinal regression analysis to anthropometric data. J Nepal Health Res Counc. 2011;9:61–6. 22929716

[40] Dorsey JL, Manohar S, Neupane S, Shrestha B, Klemm RDW, West KP Individual, household, and community level risk factors of stunting in children younger than 5 years: findings from a national surveillance system in Nepal. Matern Child Nutr. 2018;14:e12434. 10.1111/mcn.12434 PMC6865942 28233455

[41] Kim R, Mejia-Guevara I, Corsi DJ, Aguayo VM, Subramanian SV Relative importance of 13 correlates of child stunting in South Asia: insights from nationally representative data from Afghanistan, Bangladesh, India, Nepal, and Pakistan. Soc Sci Med. 2017;1847:144–54. 10.1016/j.socscimed.2017.06.017 28686964

[42] Krishna A, Mejía-Guevara I, McGovern M, Aguayo V, Subramanian SV Trends in inequalities in child stunting in South Asia. Matern Child Nutr. 2018;14:e12517. 29048726 10.1111/mcn.12517 PMC6519254

[43] Gaurav K, Poudel IS, Bhattarai S, Pradhan PMS, Pokharel PK Malnutrition status among under-5 children in a hill community of Nepal. Kathmandu Univ Med J. 2014;12:264–8. 10.3126/kumj.v12i4.13732 26333581

[44] Miller LC, Joshi N, Lohani M, Rogers B, Mahato S, Ghosh S, Webb P Women's education level amplifies the effects of a livelihoods-based intervention on household wealth, child diet, and child growth in rural Nepal. Int J Equity Health. 2017;16:183. 29047376 10.1186/s12939-017-0681-0 PMC5648516

[45] Dancer D, Anu R Maternal autonomy and child nutrition: evidence from rural Nepal. Indian Growth Dev Rev. 2009;2:18–38.

[46] Sarki M, Robertson A, Parlesak A Association between socioeconomic status of mothers, food security, food safety practices and the double burden of malnutrition in the Lalitpur district, Nepal. Arch Public Heal. 2016;74:35. 10.1186/s13690-016-0150-z PMC5020528 27625786

[47] Sreeramareddy CT, Ramakrishnareddy N, Mayoori S Association between household food access insecurity and nutritional status indicators among children aged <5 years in Nepal: results from a national, cross-sectional household survey. Public Health Nutr. 2015;18:2906–14. 25435296 10.1017/S1368980014002729 PMC10271803

[48] Psaki S, Bhutta ZA, Ahmed T, Shamsir A, Bessong P, Munirul I, John S, Kosek M, Lima A, Nesamvuni C et al. Household food access and child malnutrition: results from the eight-country MAL-ED study. Popul Health Metr. 2012;10::24. 23237098 10.1186/1478-7954-10-24 PMC3584951

[49] Paudel R, Pradhan B, Wagle RR, Pahari DP, Onta SR Risk factors for stunting among children: a community based case control study in Nepal. Kathmandu Univ Med J. 2012;10:18–24. 10.3126/kumj.v10i3.8012 23434956

[50] Singh GCP, Manju N, Grubesic RB, Connell FA Factors associated with underweight and stunting among children in rural Terai of Eastern Nepal. Asia Pacific J Public Heal. 2009;21:144–52. 10.1177/1010539509332063 19251720

[51] Chaparro C, Oot L, Sethuraman K Nepal Nutrition Profile. Washington, DC: FHI 360/FANTA; 2014.

[52] Lamichhane DK, Leem JH, Kim HC, Park MS, Lee JY, Moon SH, Ko JK Association of infant and young child feeding practices with undernutrition in children: evidence from the Nepal Demographic and Health Survey. Paediatr Int Child Heal. 2016;36:260–9. 10.1080/20469047.2015.1109281 26863233

[53] Busert LK, Neuman M, Rehfuess EA, Sophiya D, Harthan J, Chaube SS, Bishnu B, Costello H, Costello A, Manandhar DS et al. Dietary diversity is positively associated with deviation from expected height in rural Nepal. J Nutr. 2016;146:1387–93. 27306894 10.3945/jn.115.220137 PMC4926845

[54] Poudel KC, Nakahara S, Okumura J, Wakai S Day-care centre supplementary feeding effects on child nutrition in urban slum areas of Nepal. J Trop Pediatr. 2004;50:116–9. 15088803 10.1093/tropej/50.2.116

[55] Pokhrel K, Nanishi K, Poudel KC, Pokhrel KG, Tiwari K, Jimba M Undernutrition among infants and children in Nepal: maternal health services and their roles to prevent it. Matern Child Heal J. 2016;20:2037–49. 10.1007/s10995-016-2023-z 27236701

[56] Cunningham K, Singh A, Headey DD, Rana PP, Karmacharya C Reaching New Heights: 20 Years of Nutrition Progress in Nepal. Washington DC: IFPRI; 2011.

[57] Shively G, Sununtnasuk C, Brown M Environmental variability and child growth in Nepal. Heal Place. 2015;35:37–51. 10.1016/j.healthplace.2015.06.008 26183566

[58] Sharma KR. Farm commercialization and nutritional status of children: the case of the vegetables, fruits, and cash crops programme in western Nepal. Food Nutr Bull. 1999;20:445–53.

[59] Restrepo-Méndez MC, Barros AJ, Black RE, Victora CG Time trends in socio-economic inequalities in stunting prevalence: analyses of repeated national surveys. Public Health Nutr. 2015;18:2097–104. 25521530 10.1017/S1368980014002924 PMC4909139

[60] Malapit HJL, Suneetha K, Quisumbing AR, Cunningham K, Tyagi P Women's empowerment mitigates the negative effects of low production diversity on maternal and child nutrition in Nepal. (Special Issue: farm-level pathways to improved nutritional status.). J Dev Stud. 2015;51:1097–123.

[61] Cunningham K, Ploubidis GB, Purnima M, Ruel M, Suneetha K, Uauy R, Ferguson E Women’s empowerment in agriculture and child nutritional status in rural Nepal. Public Health Nutr. 2015;18:3134–45. 25797070 10.1017/S1368980015000683 PMC10271315

[62] Kattel S, McNeil N, Tongkumchum P Social determinants of linear growth among under five years children in Nepal. Pertanika J Soc Sci Humanit. 2017;25(2):851–9.

[63] Panter-Brick C Seasonal growth patterns in rural Nepali children. Ann Hum Biol. 1997;24:1–18. 9022902 10.1080/03014469700004732

[64] Shrestha B Nutritional status of under-five children in western Nepal. J Nepal Paediatr Soc. 2014;34:119–24.

[65] Panter-Brick C, Lunn PG, Langford RM, Makhan M, Manandhar DS Pathways leading to early growth faltering: an investigation into the importance of mucosal damage and immunostimulation in different socio-economic groups in Nepal. Br J Nutr. 2009;101:558–67. 18662426 10.1017/S000711450802744X

[66] Victora CG, Huttly SR, Fuchs SC, Olinto MT The role of conceptual frameworks in epidemiological analysis: a hierarchical approach. Int J Epidemiol. 1997;26:224–7. 9126524 10.1093/ije/26.1.224

[67] Black RE, Allen LH, Bhutta ZA, Caulfield LE, de Onis M, Ezzati M, Mathers C, Rivera J. Maternal and Child Undernutrition Study Group Maternal and child undernutrition: global and regional exposures and health consequences. Lancet. 2008;371:243–60. 18207566 10.1016/S0140-6736(07)61690-0

[68] World Health Organization, UNICEF WHO Child Growth Standards and The Identification of Severe Acute Malnutrition in Infants and Children. [Internet] 2009 Available from: www.who.int/childgrowth/standards. 24809116

[69] Victora CG, De Onis M, Hallal PC, Blössner M, Shrimpton R Worldwide timing of growth faltering: revisiting implications for interventions. Pediatrics. 2010;125:e473–80. 20156903 10.1542/peds.2009-1519

[70] UCLA Institute for Digital Research & Education How can I run a Piecewise Regression in Stata?. [Internet] 2019 Available from: https://stats.idre.ucla.edu/stata/faq/how-can-i-run-a-piecewise-regression-in-stata/.

[71] Barros A, Victora CG Measuring coverage in MNCH: determining and interpreting inequalities in coverage of maternal, newborn, and child health interventions. PLoS Med. 2013;10:e1001390. 23667332 10.1371/journal.pmed.1001390 PMC3646214

[72] Kakwani N, Wagstaff A, Van Doorslaer E Socioeconomic inequalities in health: measurement, computation, and statistical inference. J Econom. 1997;77:87–103.

[73] International Center for Equity in Health. Equity Analyses [Internet]. Pelotas, Brazil; Available from: https://www.equidade.org/.

[74] Van Genuchten M, Hatton L Compound annual growth rate for software. IEEE Softw. 2012;29:19–21.

[75] Strumpf EC, Harper S, Kaufman JS Fixed Effects and Difference-in-Differences. Methods in Social Epidemiology. Second Edition, edited by Oakes JM and Kaufman JS Jossey-Bass, San Francisco CA; 2017.

[76] Sandelowski M Sample size in qualitative research. Res Nurs Heal. 1995;18:179–83. 10.1002/nur.4770180211 7899572

[77] Green J, Browne J Principles of Social Research. Green J, and Browne J editors. Maidenhead: Open University Press; 2009.

[78] GTZ Free Health Care in Nepal: Findings of a Rapid Assessment. Kathmandu, Nepal: Health Sector Support Programme (HSSP), Ministry of Health and Population (MoHP); 2009.

[79] Health Sector Reform Support Programme A Summary of the Free Cost of Health Care in Nepal. [Internet] 2008 Available from: http://www.hsrsp.org/pdf/Technical_Brief_1_-Costing_Summary.pdf.

[80] Mishra SR, Khanal P, Karki DK, Kallestrup P, Enemark U National health insurance policy in Nepal: challenges for implementation. Glob Health Action. 2015;8:28763. 26300556 10.3402/gha.v8.28763 PMC4546934

[81] An Analytical Report on National Survey of Female Community Health Volunteers of Nepal. [Internet].2007. Available from: https://dhsprogram.com/pubs/pdf/FR181/FCHV_Nepal2007.pdf.

[82] The Government of Nepal. National Safe Motherhood Plan (2002–2017). [Internet] 2002 Available from: http://www.nnfsp.gov.np/PublicationFiles/3b106da9-8411-45f6-85d3-0ded0fe80469.pdf.

[83] Government of Nepal Ministry of Health and Population National Policy on Skilled Birth Attendants. [Internet]. 2006. [cited 2 May, 2018] ; Available from: http://www.mohp.gov.np/app/webroot/upload/files/Safe Motherhood and SBA Policy.pdf.

[84] Alderman H, Headey D The timing of growth faltering has important implications for observational analyses of the underlying determinants of nutrition outcomes. PLoS One. 2018;13:e0195904. 29694431 10.1371/journal.pone.0195904 PMC5919068

[85] Price JI, Bohara AK Maternal health care amid political unrest: the effect of armed conflict on antenatal care utilization in Nepal. Health Policy Plan. 2013;28:309–19. 22773608 10.1093/heapol/czs062

[86] Gillespie S, Hodge J, Yosef S, Pandya-Lorch R Nourishing Millions: Stories of Change in Nutrition. [Internet] 2016 Available from: http://ebrary.ifpri.org/utils/getfile/collection/p15738coll2/id/130395/filename/130606.pdf.

[87] West KP, LeClerq SC, Shrestha SR, Wu LS, Pradhan EK, Khatry SK, Katz J, Adhikari R, Sommer A Effects of vitamin A on growth of vitamin A-deficient children: field studies in Nepal. J Nutr. 1997;127:1957–65. 9311951 10.1093/jn/127.10.1957

[88] Joshi AR Maternal schooling and child health: preliminary analysis of the intervening mechanisms in rural Nepal. Health Transit Rev. 1994;4:1–28. 10147162

[89] WHO UNICEF JMP. [Internet] 2019; [cited 25 Feb, 2019]. Available from: https://washdata.org/data/household#!/npl.

[90] Sah N, Determinants of Child Malnutrition in Nepal: A Case Analysis from Dhanusha, Central Terai of Nepal. J Nepal Health Research Council. 2(2).

[91] NPC, CBS, WFP, World Bank, UNICEF Nepal Thematic Report on Food Security and Nutrition 2013. 2013;99:[Internet]. Available from: http://documents.wfp.org/stellent/groups/public/documents/ena/wfp256518.pdf?_ga=1.182793378.523972516.1476818874.

[92] Shively GE Infrastructure mitigates the sensitivity of child growth to local agriculture and rainfall in Nepal and Uganda. Proc Natl Acad Sci. 2017;114:903–8. 28096416 10.1073/pnas.1524482114 PMC5293016

[93] Humphrey JH, Mbuya MN, Ntozini R, Moulton LH, Stoltzfus RJ, Tavengwa NV, Mutasa K, Majo F, Mutasa B, Mangwadu G et al. Independent and combined effects of improved water, sanitation, and hygiene, and improved complementary feeding, on child stunting and anaemia in rural Zimbabwe: a cluster-randomised trial. Artic Lancet Glob Heal. 2019;7:132–79.

[94] Maleta KM, Manary MJ WASH alone cannot prevent childhood linear growth faltering. [Internet] The Lancet Global Health. 2019 Available from: www.thelancet.com/lancetgh. 10.1016/S2214-109X(18)30420-0 30554752

[95] Luby SP, Rahman M, Arnold BF, Unicomb L, Ashraf S, Winch PJ, Stewart CP, Begum F, Hussain F, Benjamin-Chung J et al. Effects of water quality, sanitation, handwashing, and nutritional interventions on diarrhoea and child growth in rural Bangladesh: a cluster randomised controlled trial. Artic Lancet Glob Hea. 2018;6:302–17. 10.1016/S2214-109X(17)30490-4 PMC5809718 29396217

[96] Null C, Stewart CP, Pickering AJ, Dentz HN, Arnold BF, Arnold CD, Benjamin-Chung J, Clasen T, Dewey KG, H Fernald LC et al. Effects of Water Quality, Sanitation, Handwashing, and Nutritional Interventions on Diarrhoea and Child Growth in Rural Kenya: A Cluster-Randomised Controlled Trial[Internet]. 2018; [cited 2 Apr, 2019] ; Available from: www.thelancet.com/lancetgh. 10.1016/S2214-109X(18)30005-6 PMC5809717 29396219

[97] The World Bank Prevalence. of underweight, weight for age (% of children under 5) | Data. [Internet] Open Data. 2019; [cited 23 Apr, 2019]. Available from: https://data.worldbank.org/indicator/SH.STA.MALN.ZS?locations=NP.

[98] The World Bank Prevalence of wasting, weight for height (% of children under 5) | Data. [Internet] Open Data. 2019; [cited 23 Apr, 2019]. Available from: https://data.worldbank.org/indicator/SH.STA.WAST.ZS?locations=NP.

[99] The World Bank Prevalence of anemia among pregnant women (%) | Data. [Internet] Open Data. 2019; [cited 23 Apr, 2019]. Available from: https://data.worldbank.org/indicator/SH.PRG.ANEM?locations=NP.

