# Supplementary material for: Drivers of stunting reduction in Nepal: a country case study
Source: Am J Clin Nutr. 2020 Sep 5;112(Suppl 2):844S–859S. doi: 10.1093/ajcn/nqaa218 (PMC7487432; doi:10.1093/ajcn/nqaa218)
Supplement: nqaa218_Supplemental_Files [file nqaa218_supplemental_files.zip › Clean_Nepal_Online_Supplementary_Appendices_July052020.docx]

**Title**: Drivers of stunting reduction in Nepal: a country case study

**Authors**: Kaitlin Conway, Dr. Nadia Akseer, Raj Kumar Subedi, Samanpreet Brar, Basudev Bhattarai, Dr. Raja Ram Dhungana, Muhammad Islam, Anustha Mainali, Nikita Pradhan, Hana Tasic, Dip Narayan Thakur, Jannah Wigle, Dr. Mahesh Maskey, Dr. Zulfiqar A. Bhutta

**Supplementary Appendices**

**List of Supplementary Appendices**

[Supplementary Appendix 1: Descriptive Analysis of Contextual Factors 2](#_Toc44880428)

[**Supplementary Figure 1A:** Trends in GDP per capita, poverty and urbanization, 1990-2016 3](#_Toc44880429)

[**Supplementary Figure 1B:** Trends in key contextual indicators,1990-2016 3](#_Toc44880430)

[Supplementary Appendix 2: Systematic Literature Review 4](#_Toc44880431)

[**Supplementary Figure 2:** Literature review flow diagram 5](#_Toc44880432)

[Supplementary Appendix 3: Multivariable Analyses Methods 20](#_Toc44880433)

[Supplementary Appendix 4: Qualitative Data Collection and Analyses Methods 23](#_Toc44880434)

[**Supplementary Table 1:** Inclusion Criteria 24](#_Toc44880435)

[**Supplementary Figure 3A:** Location of Lalitpur district in Nepal 25](#_Toc44880436)

[**Supplementary Figure 3B:** Lalitpur Map displaying the study sites for FGDs and in-depth interviews 25](#_Toc44880437)

[Supplementary Appendix 5: Quantitative Results 28](#_Toc44880438)

[**Supplementary Figure 4A**: Spline analysis of inflection points of change in the slope of HAZ, 1996 28](#_Toc44880439)

[**Supplementary Figure 4B**: Spline analysis of inflection points of change in the slope of HAZ, 2001 28](#_Toc44880440)

[**Supplementary Figure 4C**: Spline analysis of inflection points of change in the slope of HAZ, 2006 29](#_Toc44880441)

[**Supplementary Figure 4D**: Spline analysis of inflection points of change in the slope of HAZ, 2011 29](#_Toc44880442)

[**Supplementary Figure 4E**: Spline analysis of inflection points of change in the slope of HAZ, 2016 30](#_Toc44880443)

[**Supplementary Figure 5A**: 1996 stunting prevalence by region 30](#_Toc44880444)

[**Supplementary Figure 5B**: 2001 stunting prevalence by state 30](#_Toc44880445)

[**Supplementary Figure 5C**: 2006 stunting prevalence by state 31](#_Toc44880446)

[**Supplementary Figure 5D**: 2011 stunting prevalence by state 31](#_Toc44880447)

[**Supplementary Figure 6A**: Change in absolute SII by year in Nepal 32](#_Toc44880448)

[**Supplementary Figure 6B**: Change in relative CIX by year in Nepal 32](#_Toc44880449)

[**Supplementary Figure 7:** Stunting prevalence disaggregated by child sex, 1996 – 2016 33](#_Toc44880450)

[**Supplementary Table 2**: Descriptive trends in stunting determinants in 2001-2016 in children <5 years 33](#_Toc44880451)

[**Supplementary Table 3:** Decomposition analysis for children among 6 – 23 months from 2001 – 2016 39](#_Toc44880452)

[**Supplementary Figure 8:** Decomposing predicted changes in HAZ among children between 6-23 months (i.e. relative ranking of product coefficients for determinant domains) from 2001-2016 40](#_Toc44880453)

[**Supplementary Table 4:** Decomposition analysis for children 24-59 months from 2001 – 2016 40](#_Toc44880454)

[**Supplementary Figure 9:** Decomposing predicted changes in HAZ among children between 24-59 months (i.e. relative ranking of product coefficients for determinant domains) from 2001-2016 41](#_Toc44880455)

[**Supplementary Table 5:** Decomposition analysis for under-5 children from 2001-2016 41](#_Toc44880456)

[**Supplementary Figure 10:** Decomposing predicted changes in HAZ among children under-5 (i.e. relative ranking of product coefficients for determinant domains) from 2001-2016 42](#_Toc44880457)

[**Supplementary Table 6:** Difference-in-differences multivariable regression for children under-5 years from 2001 - 2016 42](#_Toc44880458)

[**Supplementary Table 7:** Difference-in-differences multivariable regression for children 24-59 months from 2001 – 2016 50](#_Toc44880459)

[**Supplementary Table 8:** Difference-in-differences multivariable regression for children 6-23 months old using DHS surveys from 2001 – 2016 59](#_Toc44880460)

[**Supplementary Table 9:** Difference-in-differences multivariable regression for children under 6 months from 2001 – 2016 70](#_Toc44880461)

[Supplementary Appendix 6: Programs and Policies 79](#_Toc44880462)

[**Supplementary Table 10**: Detailed timeline of nutrition-specific and -sensitive laws, policies and programs in Nepal 79](#_Toc44880463)

[Supplementary Appendix 7: Qualitative Results 91](#_Toc44880464)

[**Supplementary Table 11:** Qualitative Inquiry – Full Results 91](#_Toc44880465)

[**Supplementary Table 12a:** Summary of National Stakeholders 91](#_Toc44880466)

[Supplementary Table 12b: Hierarchy of Nutrition-Specific and –Sensitive Policy Events 95](#_Toc44880467)

[Acknowledgments 113](#_Toc44880468)

[**Supplementary Appendices References** 113](#_Toc44880469)

# **Supplementary Appendix 1:** Descriptive Analysis of Contextual Factors

## **Supplementary Figure 1A:** Trends in GDP per capita, poverty and urbanization, 1990-2016

Sources: (1–3)

## **Supplementary Figure 1B:** Trends in key contextual indicators,1990-2016

Source: (4–8)

# **Supplementary Appendix 2:** Systematic Literature Review

**Methods**

Search terms employed included "stunting" or "linear growth" or "linear growth stunting" or "HAZ" or "height" or "height-for-age" or "LAZ" or "length" or "length-for-age" or "undernutrition" or "malnutrition" or "nutr*" AND “child*” or “infan*” AND “Nepal*.” Published peer-reviewed literature were located within 15 online databases, including MEDLINE, Embase, AMED, CAB Abstracts, CINAHL, Cochrane CENTRAL, Campbell Collaboration, EPPI Centre Trials Register (TRoPHI), 3ie, JOLIS, African Journals Online, WHOLIS, LILACS, Scopus, and Web of Science. Grey literature was found by searching websites for UNICEF, WHO, UNDP, WFP, FAO, World Bank Group Open Knowledge Repository, Asian Development Bank, Nutrition International, Global Alliance for Improved Nutrition, International Food Policy Research Institute, and the Government of Nepal including the Ministry of Health, Ministry of Agriculture and Livestock Development and the Central Bureau of Statistics.

The exported set of records were de-duplicated and screened for relevance. Records were included if they met all of the following inclusion criteria:

i) included an under-5 population in Nepal;

ii) published between 1990-2017;

iii) examined one or more of the determinants of chronic undernutrition (e.g. determinants, risk factors, policies, programs, interventions, or initiatives); and

iv) examined effects on child growth or a reduction in stunting

Of the 4,604 articles initially found in this process, 109 were ultimately included in the literature review based on title/abstract and full-text screening. This included 84 published peer-reviewed articles and 25 pieces of grey literature. Additional targeted searches were completed for more information on key topics to supplement and expand on important factors in the Nepal stunting narrative, resulting in 428 additional documents (**Supplementary Figure 2**).

**Supplementary Figure 2:** Literature review flow diagram
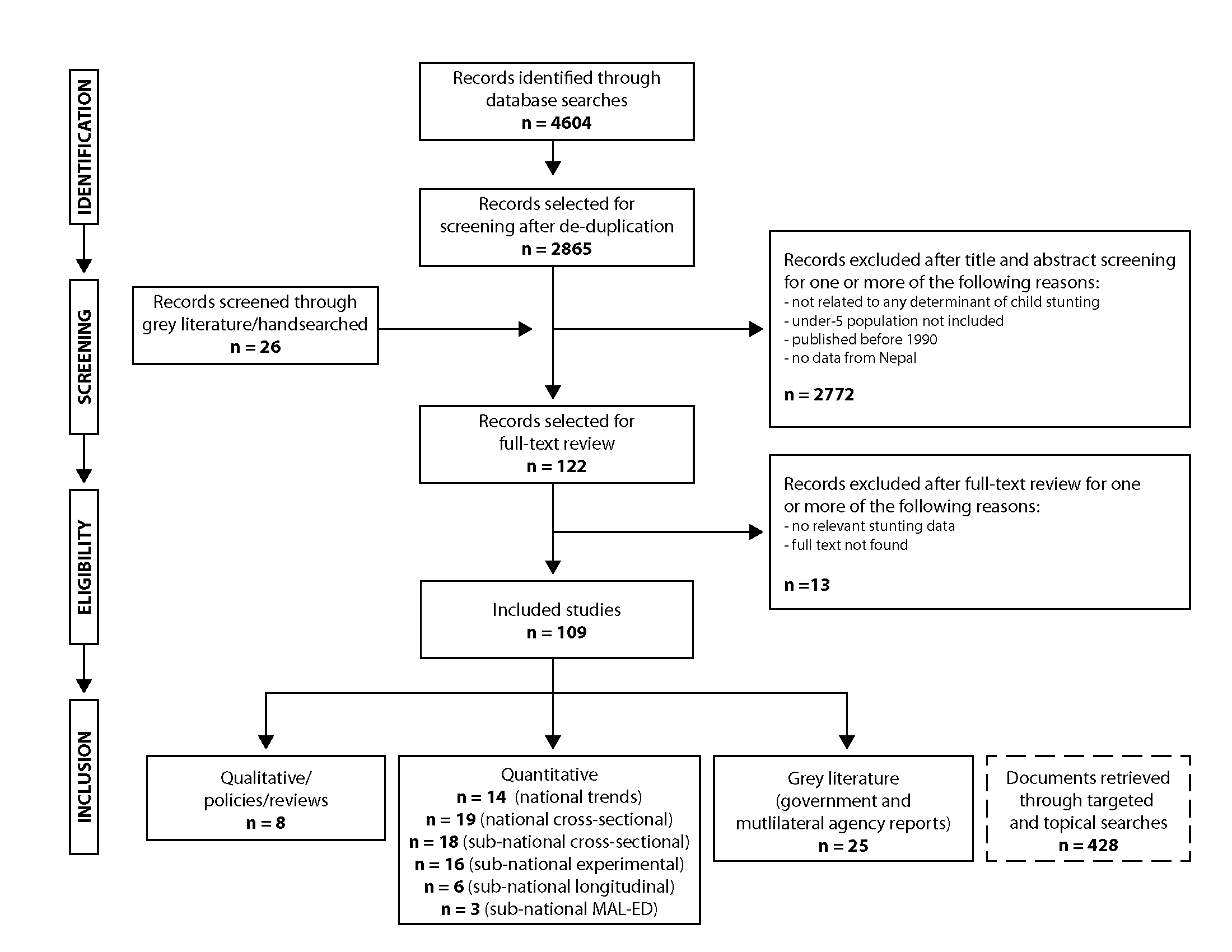


**Full Literature Review**

Factors that have contributed to a reduction in stunting in Nepal are presented in relation to an adapted version of UNICEF’s conceptual framework for malnutrition (9). In particular, high levels of poverty, low levels of education, low levels of improved water and sanitation, and a lack of accessibility within the health system contributed to chronic malnutrition among children in Nepal. Over the last several years, key events such as the establishment of democracy and the end to political unrest, increased education and women’s empowerment, poverty reduction through remittance incomes, improvements in water and sanitation and the decentralization as well as strengthening of the health system have targeted the basic, underlying and immediate causes of malnutrition. Along with this, a recent increase in coordinated, multi-sectoral nutrition-specific and nutrition sensitive policies and programs has allowed for a more focused approach to improving the nutritional status of the people of Nepal. The aim of this literature review is to summarize data from programs, policies, and intervention studies that relate to the causes of chronic malnutrition in Nepal.

**Basic causes (Distal)**

*Environmental Context and Social Demographics*

Nepal is a landlocked country, sharing borders with India to the south, east and west and China to the north. Nepal is made up of three distinct ecological zones, including mountains, hills and Terai plains. Inequalities in health, education and wealth continue to exist and impact malnutrition outcomes across the country’s different regions, particularly among the hard to reach remote areas (10–12). Geographically speaking, stunting in Nepal is found to be more prevalent in rural rather than urban areas (51% compared to 36% in 2011), and is also more prevalent within the country’s remote mountain and hill regions where approximately 60% of children are stunted compared to 46.3% in the Terai region (12–18). A study by Gaire et al. in 2016 found that children who lived in mountain regions were significantly more likely to be stunted than those from the Terai region, with an Adjusted Odds Ratio (AOR) = 1.52 (95% CI: 1.01, 2.31, p = 0.048) (17).

A nutritional survey conducted by UNICEF in districts within Nepal’s mountain region affected by drought and other severe weather conditions showed that 72% of children in Bajura district were stunted, while in Jumla district stunting rates reached as high as 82.3% (19). The impact of severe weather conditions such as drought on child growth outcomes in Nepal is supported by a study by Shively et al. conducted in 2017 using DHS data from 2001, 2006 and 2011. Findings from linear and multi-level regression showed that a one standard deviation increase in agricultural rainfall (about 110 milimeters) was associated with a mean increase in HAZ between 4% and 13% (20). Conversely, however, other studies have warned of the impact of rainfall shocks during monsoon seasons on HAZ in Nepal, with some indication that a monsoon rainfall and its effect on agriculture can lead to growth faltering in children under five, though this is not shown to be permanent (21). Similarly, the aforementioned study by Gaire et al. in 2016 showed through bivariate and multivariate analyses of 2011 DHS data that flooding in Nepal can impact child stunting among those 6-59 months, with an AOR = 0.57 (95% CI: 0.31, 0.96) for severe stunting, and AOR = 0.66 (95% CI: 0.41, 0.94) for moderate stunting (17).

Ethnically speaking, Nepal’s population consists of 125 different ethnic groups/castes (10). A review of food security and nutrition issues in Nepal conducted by Shively et al. in 2011 found that among household socioeconomic indicators, being a member of a minority group increased the probability of a child being stunted and underweight (13). In 2016, Gaire et al. found that Nepalese children who belonged to the Brahmin/Chhetri (AOR =0.36; 95% CI: 0.18, 0.69; p=0.002); Janajati (AOR =0.38; 95% CI: 0.20, 0.74; p=0.004); and Muslim ethnic groups (AOR =0.30; 95% CI: 0.09, 0.98; p=0.046) were comparatively less likely to be severely stunted than those from Dalit and other ethnic groups (17). Another study conducted in 2015 by Brainerd and Menon used linear regression to understand differences in child health based on religious affiliation. In Nepal, it was found that Hindu children had higher height for age (HAZ) z-scores compared to Muslim children within the rural population, with Hindu infant boys in particular being significantly taller than Muslim infant boys. Though Muslim infants had the same or greater HAZ scores at the beginning, over the long term it was found that Hindu children gained an advantage in HAZ that began after 12 months of age (22).

*Political Context*

Over the past three decades, Nepal has undergone a process of democratization marked by prolonged periods of political instability. Beginning in 1990, then King Birenda agreed to the creation of a democratic constitution following mounting public pressure. In 1996, a Maoist revolution broke out with the goal of completely abolishing Nepal’s long-standing monarchy. The result of this Maoist insurgency was a decade long armed conflict, known as the “people’s war.” This resulted in more than 12,000 people being killed, 200,000 people being internally displaced and an estimated 2 million people crossing the border into India (23–27). Throughout the conflict, the hills region was one of the first and most consistently affected areas of the country (27).

In the midst of this conflict, in 2001, the Crown Prince Dipendra killed several members of the royal family during a dinner party at the palace, including King Birenda and Queen Aishwarya, before taking his own life. The event became known as the “royal massacre,” and led to even greater political instability in the country. Following two failed attempts at a ceasefire in 2001 and 2003, the Maoist revolution came to an end in 2006 with the signing of a peace deal and the declaration of Nepal as a federal democratic republic (26). The democratization of Nepal was finalized in the summer of 2008 with the election of the country’s first president, Ram Baran Yadav, ending the country’s 239 year-long monarchy. Somewhat surprisingly, stunting in Nepal continued to decline throughout the years of armed conflict, reducing from 56.6% in 1996 to 49.3% by the conflict’s end in 2006 (27).

*Economic Growth and Migration*

Despite political instability, Nepal’s Gross Domestic Product (GDP) has been on the rise over the past several decades, increasing from US $508.3 million in 1990 to US $24.5 billion in 2017 (1). Government expenditure on health has fluctuated between 2000 and 2015, though it has increased overall, from 3.57% of GDP in 2000 to 6.15% of GDP in 2015 (28). Health sector expenditure per capita has also increased over the last several years, from US $8.6 in 2000 to US $44.4 in 2015 (29).

An important component of Nepal’s economic growth has been remittance incomes due to outward migration over the last several decades, with one study attributing remittances to have had a 27% stake in poverty reduction in the country between 1995 and 2010 (24,30). Between 1991 and 2011, the percentage of Nepalese living abroad more than doubled, from 3.4% to 7.3% (10,31). During this same time period, the remittances being sent back to Nepal from those working abroad increased from 2.1 billion rupees to 434.6 billion rupees, with an increasing number of migrants travelling to more lucrative positions in the Gulf States compared to past years when neighboring India was the most popular destination for foreign employment (24). According to the Nepal Living Standards Survey 2010-2011, recipients of remittances in Nepal spend approximately 79% of what they receive on daily consumption, and about 56% of households in the country receive remittances (32).

*Wealth Index*

Between 1995 and 2010, Nepal’s poverty rate dropped by 43 percentage points, from 68% to 25%. Besides remittances, some have tied the reduction of poverty in the country to growth in the agricultural sector at 3.8% per year between 2001 and 2011, and a strong recovery of the tourism sector following the end of the armed conflict in 2006 (24).

A decline in the poverty rate over the past several decades can be seen as important to reductions in stunting in Nepal, as several studies have found a connection between household wealth and stunting outcomes. A study by Devkota et al. conducted in 2016 found that children in the poorest households in the country are more than twice as likely to be stunted as children in the wealthiest households (56% versus 26%) (12). This supports an earlier subnational study conducted in 2010 by Osei et al., who found through multiple logistic regression modelling that households in the lower third of the wealth index had 2.21 times the odds of being stunted compared to those in the upper third (OR= 2.21, CI = 1.10-4.44) (33). This finding is further supported by similar results from 9 other studies, including a cross-sectional study by Pradhan et al. in 2011 and Niraula et al. in 2013; a retrospective cohort study by Nisar et al. in 2016; bivariate and multivariate analyses by Gaire et al. in 2016; and regression analysis by Tiwari et al. in 2014, Shively and Sununtnasuk in 2015, and 2017 studies by Dorsey et al. Kim et al., and Devakumar et al. (15,17,18,34–39).

Corresponding to this, asset accumulation, sometimes used as a proxy for improvements in wealth, has come out as important in several other studies as a contributor to changes in HAZ in Nepal (24,40–42). Despite the decline in the poverty rate outlined above, it should be noted that a number of studies found persistent inequalities in terms of stunting decline by wealth quintile in Nepal, with larger declines seen in wealthier groups compared to the poorest groups (14,43,44).

*Maternal Education and Empowerment*

Improvements in education, including maternal education, can be seen as important in the decline of stunting among children in Nepal. This has been linked to the impacts maternal education can have on the utilization of health services, changes in household health behaviours, and increased attention paid to dietary diversity for children (45,46). In their 2009 study, Dancer and Rammohan used 2006 DHS data from Nepal in Ordinary Least Squares (OLS) and logit models to show the positive impact of mother’s education on their child’s nutritional outcomes. Maternal primary education was shown to be important for reducing the odds of being stunted in all children in the study and when looking at girls only (though only at the 10% level). Maternal primary education did not, however, have an effect when looking at boys only. Along with this, the study found that having a mother with secondary education and above compared to no education was shown to dramatically reduce the odds of being stunted in all children, with a slightly larger effect in girls (nearly 2 times less likely to be stunted) compared to boys (1.45 times less likely to be stunted). In this study, paternal education had no significant impact on stunting (47).

This gendered impact of maternal education on children’s stunting outcomes was also seen within a 2016 study by Sarki et al. In this study, findings of a statistical analysis of socioeconomic, food safety-related and anthropometric data from 289 mother-child pairs showed that higher education levels among mothers was associated with a higher HAZ score in female children, but not for male children (48).

Findings on the impact of maternal education specifically on stunting outcomes are also supported by a modeling of the intermediate determinants of stunting in several other studies (24,36,40,42,43,46,47,49). Decomposition analysis of DHS data from 2001, 2006 and 2011 conducted by Headey and Hoddinott in 2015 showed that the difference in HAZ between the child of a mother with no education and one with six years of education (i.e. completing primary school) was about 0.17 standard deviations, and 0.34 standard deviations for completing secondary school. The impact of paternal education on HAZ was noted in this study, though it is shown to have about one fourth the impact of maternal education (25).

Over the last several decades, there has been increasing focus in Nepal on improving educational outcomes for the entire population. Beginning in 1999, the World Bank supported the 10-year Basic and Primary Education Project with the goal of decentralizing management of schools to the community-level for quality improvement and better distribution of resources to marginalized groups. The Basic and Primary Education Project also sought to improve the quality of teaching and educational content in schools and increase access to education (50). Between 2000 and 2015, the Education for All plan promoted early child development activities and increasing access to education, particularly for the poor and marginalized. Overlapping with this period was an increase in literacy rates for 15-24 year olds from 49.6% in 1990 to 88.6% in 2011, and an increase in the female literacy rate among those 15 years and older from 17% in 1990 to 47% in 2011. Furthermore, the proportion of women with no education was cut in half between 1996 and 2011, with maternal education increasing from 1.2 years in 1996 to 3.9 years in 2011, representing an annual change of 15.0% (23,51).

Beginning in 2007, the Non-Formal Education Policy was enacted to target marginalized groups including women and girls, the poor and those living in remote areas of the country into out-of-school education activities. This policy allows for literacy, post-literacy and awareness raising programming, as well as programs that provide continued education, skills development and income generation (26,52). One example of non-formal education programming in Nepal is mother’s groups. These community-based groups allow women to gather for the purpose of knowledge-sharing and learning and have been shown to be important for women’s empowerment and reducing the effects of chronic malnutrition among participants’ children through activities such as growth monitoring (53).

A 2015 cross-sectional survey of 4080 households by Cunningham et al. examined the association between women’s in agriculture and nutritional status among children under 2 years of age in 240 rural communities across sixteen districts in Nepal. The study found that women’s empowerment in agriculture was positively associated with length-for-age z-scores (LAZ) (β=0.20, p=0.04), as were satisfaction with leisure time (β=0.27, p<0.01), access to and decisions regarding credit (β=0.20, p=0.02) and autonomy in production (β=0.10, p=0.04) (54). This is supported by a review of the literature on nutrition issues in Nepal by Shiveley et al. in 2011, who found that the odds of a child being stunted decreased when mothers had more autonomy in decision-making (13).

In another study by Malapit et al. in 2015, similar analysis of the relationship between women’s empowerment in agriculture and production diversity on both maternal and child dietary diversity and anthropometric outcomes found that children have better diets and long-term nutritional status in households where there is greater equality between women and men. It was also noted that mothers with greater control over spending within the household were more likely to have children with better long-term nutritional status (55). This highlights the important role of women’s empowerment overall for improving child growth outcomes in Nepal.

These increases in literacy, education and women’s empowerment have also been taking place alongside a decline in early marriage in Nepal, with the number of women aged 15-19 years who were not married rising from 60% in 2000 to 71% in 2011 (23). Data from the World Bank also indicates that the age at marriage for women in the country has also been impacted by outward migration of a predominantly male population for work abroad (56).

Despite the gains that have been made, however, gender inequalities in education still exist in Nepal, particularly in the number of years and quality of education received by boys and men compared to girls and women (26,51). Nepal’s Annual Household Survey for 2015/2016 found that the male adult literacy rate had reached 74.2% of the population while the female literacy rate was 58.2% (57). Similar disparities also exist between urban and rural populations, with the literacy rate of those 5 years and older in 2011 82.2% in urban areas versus 62.5% in rural areas (58).

**Underlying causes**

*Food Security*

Among the important underlying causes of stunting in Nepal is food security. Variations in food security continue to exist within the population of Nepal both regionally and between wealth quintiles. Nearly half of households in the bottom wealth quintile are food insecure. Those living in the more remote mountain zone tend to experience severe food insecurity more often than the other regions, relying on their own household food production for daily consumption, whereas those in the rural hills and Terai tend to purchase the bulk of their food (59,60). Nepal has been a net importer of food for the last decade, as it has experienced a prolonged period of agricultural stagnation (26,61).

In a 2010 study by Osei et al., data was analyzed from a cross sectional survey of families of 368 children aged between 6 and 23 months living in one of Nepal’s Terai districts called Kailali. The study found that more than two-thirds (69%) of households were food insecure, and that the prevalence of stunting was slightly higher among children in food-insecure homes, though it did not find a statistically significant association between household food insecurity and stunting (33). A community-based case control study of 354 children (118 cases and 236 controls) by Paudel et al. conducted in 2012 did find that being a food deficit family was a risk factor for stunting with an odds ratio of 4.26 (CI 1.73-10.45) (62). Similar findings to those above were reported in a study by Sreeramareddy et al. in 2015 using data from the 2011 Nepal DHS, as well as by Psaki et al. in an eight-country MAL-ED study conducted in 2012, which included Nepal (63,64).

A study by Shively and Sununtnasuk in 2015 that utilized regression modeling found that for children, especially under 24 months, increases in overall yield of household agricultural production are associated with significant improvements in HAZ and decreased probabilities of stunting. However, they also found that higher ratios of own-consumption of agriculture was associated with lower HAZ and increased probabilities of stunting. In terms of specific crop production, higher proportions of root production was correlated to reductions in the probability of stunting, and production of animal products showed improvements in HAZ by nearly 0.25 standard deviations (18).

In recent years, the government of Nepal and its development partners have also made efforts in the form policies and programs in order to improve agricultural outputs. In 2004 the World Bank-supported Poverty Alleviation Fund was launched to improve rural welfare in the 40 poorest districts in the country. A 2012 evaluation of the project found a statistically significant causal impact of the program on key welfare outcomes, including a 19% decrease in the incidence of food insecurity (65). A 2014 longitudinal randomized control trial by Miller et al. looked at six communities in the Terai and hills of Nepal that were randomly assigned to receive community development and poverty alleviation activities from the organization Heifer Nepal. Activities provided by Heifer Nepal centered on livestock training, and it was found that at 12 months, the Terai intervention group had improved child weight (p = 0.04), child height (p = 0.05) and sanitation practices (p <0.01), reduced sick days (p = 0.03), as well as increasing household income (p = 0.004), ownership of animals (p = 0.04)., and land (p = 0.04). Longer participation in the program was also associated with better growth outcomes, showcasing the potential impact of poverty alleviation programs to indirectly improve child growth (66).

Another randomized control trial by Osei et al. in 2017 tested the impact of an enhanced homestead food production program in rural areas of Baitadi District, Nepal among 2106 and 2614 mother–child pairs (children aged 12-48 months) at baseline and follow-up, respectively. They found that this integrated intervention, which included home gardening, backyard poultry rearing and nutrition behavior change communication played an important role in improving household food security and child feeding practices, along with other positive impacts to maternal and child health. Specifically, they found that between baseline and post-intervention, food insecure households decreased by 26% in the intervention group, and that the proportion of children in the intervention group who were fed the WHO-recommended minimum acceptable diet improved by 44 percentage points compared to 6 percentage points among the control group (67).

Noteably, in the Nepal Living Standards Survey (NLSS) from 2010/11 remittance incomes and urbanization within Nepal were indicated to have helped with both food insecurity and a reduction in poverty (59). More recent programs aimed at improving food security include the FAO supported Food and Nutrition Security Plan which began in 2013, and the 20 year Agricultural Development Strategy which began in 2014, though these have yet to be evaluated (68,69).

*Infant and Young Child Feeding*

Infant and young child feeding practices, including exclusive breastfeeding, complementary feeding and dietary diversity are seen as important to maintaining overall child nutritional status and reducing stunting, however the literature indicates that there is room for improvement among these indicators in Nepal.

Though the percentage of children ever breastfed in Nepal between 1996 and 2016 is remarkably high at 98% and 99%, respectively, data suggests that the rate of exclusive breastfeeding in Nepal has declined over the same period, from 75% to 66% (70). Data also suggests that rates of exclusive breastfeeding, complementary feeding and dietary diversity have remained stagnant in Nepal over the last several years, though initiation of breastfeeding within one hour of birth and meal frequency have increased. Regression analysis using DHS data from 1996, 2001, 2006 and 2011 by Cunningham et al in 2016 found that there had been -1.1% change in exclusive breastfeeding for children 0-6 months of age between 2001 and 2011, and a 0.8% increase in the percentage of children aged 6-8 months who were being fed any solid foods. While there had only been a 0.7% decline in the number of children 6-24 months of age who were fed 4 types of food between 2001 and 2011, there was a noticeable increase of 12.8% in the number of children aged 6 to 23 months who were fed 3 or more times a day (24). These findings echo similar ones outlined in a study conducted by Headey and Hoddinott in 2015 (40).

A review of sub-national analyses of infant and young child feeding practices in Nepal further support the idea that there is much room for improvement in this area. In 2010, Osei et al. conducted data analysis from a cross sectional survey of children 6-23 months from 368 families in a Terai district of the country. Results showed low numbers of appropriate infant and young child feeding, with early initiation of breastfeeding occurring among only 64.9% of respondents (n=285); timely introduction of complementary feeding occurring among 62.1% of respondents, and minimum dietary diversity taking place among only 13% of respondents (33). A 2009 study of 443 rural Terai children aged 6 to 36 months in Eastern Nepal by Singh et al. found that only 30.5% of the 338 children 1 year of age or more within their study population were exclusively breastfed for 9 months or more, and that among stunted children it was 45.6% (71). Furthermore, in a 2012 community-based case control study conducted in mid-west Nepal by Paudel et al., data showed that inappropriate exclusive breastfeeding (OR=6.90, 95% CI: 2.81, 16.97), complementary feeding less than four times a day (OR=3.60, 95% CI: 1.32, 9.95) and dietary diversity below the WHO standard (OR=4.06, 95% CI: 1.70, 9.67) were all risk factors for stunting (62).

Data compiled by Chaparro et al. in 2014 indicated that the greatest increases in stunting prevalence in Nepal were occurring between 9 and 18 months of age due to suboptimal breastfeeding and complementary feeding, coupled with a rise in illness and/or infection rates at this age (68). This phenomenon of higher rates or worsening in the severity of stunting in older infants has also been seen in five other studies by Panter-Brick in 1997, Osei et al. in 2010, Shrestha et al. and Gaurav et al., both in 2014, and Kattel et al. in 2017 (33,49,72–74).

A study by Tiwari et al. in 2014 found through regression analysis of Nepal’s DHS data from 2011 that prolonged breastfeeding, i.e. beyond 12 months, was a significant risk factor for stunting and severe stunting among children aged 0-23 months and 0-59 months, with an AOR for stunted children aged 0–23 months = 2.60 (95% CI: 1.87, 4.02); AOR for severely stunted children aged 0–23 months = 2.87 (95% CI: 1.54, 5.34); AOR for stunted children aged 0–59 months = 3.54 (95% CI: 2.41, 5.19) and AOR for severely stunted children aged 0–59 months = 4.15 (95% CI: 2.45, 6.93) (38).

In 2015, Lamichhane et al. conducted multiple linear and logistic regressions of 2011 Nepal DHS data and found both dietary diversity index and minimum dietary diversity to be associated with a higher HAZ as well as lower odds of being stunted in children ages 0-23 months. With each increasing dietary diversity index point, HAZ rose by 0.1 (p<0.05); and when child age, gender and maternal education were controlled for, minimum dietary diversity was significantly associated with the log odds of stunting (OR=0.6, p<0.05). It was also found that adjusted mean HAZ was higher in children who obtained minimum dietary diversity (75).

Similar findings can be seen in a 2016 study by Busert et al. In this study, the importance of dietary diversity in reducing the risk of stunting and improving growth following growth faltering in children was observed in cohort data of children 0-59 months who were followed up after 9 and 29 months. After adjustment, increasing dietary diversity by just one food group was found to be associated with a 0.09 cm (95% CI: 0.00, 0.17 cm) increase in conditional growth in the second observation period (76).

A study by Poudel et al. (2004) showed the potential positive impact of supplementary feeding of a noon-time meal and afternoon snacks to children ages 1-5 within one of two day care settings within the urban slums of Nepal. In the study, the nutritional status of the children attending these two daycare centers (one which was government run and one run by a non-governmental organization) was compared to the nutritional status of non-attendee children from the same neighbourhood. Compared to the attendees of both daycare centres, non-attendees were more likely to be stunted with an OR = 3.5 (95% CI: 1.04, 11.83), and also tended to be more underweight with an OR=2.1 (95 % CI: 0.51, 8.40), though the likelihood of stunting in non-attendees compared to attendees was greater when comparing against the NGO-run daycare. Results also suggested that the longer the attendance at a daycare centre, the greater the improvement in a child’s nutritional status, particularly related to HAZ (77).

*Access to Health Services*

Improvements in child growth outcomes in Nepal have been linked to increased access to and utilization of health services over the last several decades (24,61). Through a variety of policies and programs beginning with the National Health Policy in 1991, Nepal has been working to decentralize the health system. There has also been an increase in coordination between the various levels of government and their external development partners towards aligning on common goals for accessibility (23,24).

Improvements in the health system have largely focused on the expansion of health institutions—by the late 2000s, the government had created 15,000 primary health outreach clinics throughout the country which are staffed by Female Community Health Volunteers (FCHVs) (40). FCHVs are able to work at the local level to deliver essential services such as Vitamin A supplementation, deworming, vaccinations and treatment of childhood illnesses, prenatal and antenatal care, iron folic acid supplementation, family planning, referral services and health education (61,68). The Government of Nepal has also been working to further improve access to health services for those living remotely by increasing road coverage (23).

In 2014, Yadav et al. conducted a case-control impact evaluation of a 12-month community-based nutrition program led by Female Community Health Workers in rural areas of the Mahottari district of Nepal. Participants included children under three years of age and their mothers, and the program showed an impact on reducing the prevalence of wasting and underweight in the intervention group, though not on stunting.

Despite the lack of effect on stunting, however, it is important to note the impact of this Female Community Health Worker-led program on key improvements in the nutritional intake among participating children. During the intervention, Vitamin A supplementation increased by 7.27% in the intervention group compared to an only 1.93% increase in the control group, (p=0.03). Initiation of breastfeeding within one hour of birth increased by 8.44% in the intervention group, while it showed a 3.52% decrease in the control group (x^2^=3.74, p=0.05). Exclusive breastfeeding up to six months also increased by 6.21% in the intervention group compared to a 3.02% decrease in the control group, though this was not found to be statistically significant (x^2^=2.52, p=0.11). Results further showed that mother’s feeding of colostrum to their children at birth increased in the intervention group by 6.7%, compared to a decrease in the control group of 1.3% (x^2^=7.37, p=0.00) (78).

Though Vitamin A supplementation is widespread in Nepal due to the proliferation of FCHVs, the reported impacts of Vitamin A supplementation on child growth have been mixed. In a randomized control trial of 3377 rural Nepalese children aged 12-60 months by West et al. in 1997, Vitamin A supplementation was shown to have no effect on linear growth or weight gain (79).

Through a coordinated, multi-sectoral effort between the Government of Nepal and development partners, efforts have also been made to specifically improve maternal and newborn health in the country, including increasing access to this type of care. The Safe Delivery Incentive Program (2005), also known as *Aama* (“Mother”) provides free delivery care and financial incentives to pregnant women who utilize antenatal and postnatal care services and deliver in a health facility (61). The National Policy on Skilled Birth Attendants (2006) was also put in place to increase the number and improve the training of this cadre of health professionals (23). Between 1996 and 2011, the percentage of women attending four or more antenatal care appointments increased from 6.3% to 29.6%, the percentage of deliveries occurring in a hospital increased from 9% to 41%; and iron supplementation during pregnancy increased from 11.6% to 82.3% (61).

These improvements are especially important for stunting reduction as several studies have linked a lack of ANC care to stunting outcomes. For example, in a study by Pokhrel et al. in 2016, regression modeling showed that the absence of ANC was positively associated with stunting for children 6-11 months (AOR 6.51; 95 % CI 2.11–20.10), and for children 12–23 months (AOR 3.32; 95 % CI: 1.50–7.31). This study also connected lack of at least one ANC visit with poor infant and young child feeding practices (80). Additionally, linear probability modeling by Headey and Hoddinott in 2015, among others, found that receiving at least four ANC visits predicted a 0.09 standard deviation improvement in HAZ, while delivery in a hospital was associated with an almost 0.20 standard deviation improvement in HAZ (15,40,41).

Though progress on access to health services and maternal and newborn care has been marked in Nepal, there still remains room for growth. For example, a survey of 4287 households across 21 Village Development Communities (VDCs) and all 3 ecological regions conducted in 2016 by USAID and partners found that 54% of non-pregnant women were anemic, rising to 59% among pregnant women (60). Another 2016 study by Nisar et al. on iron folic acid (IFA) supplementation among pregnant women in Nepal found the adjusted relative risk of being stunted was 14% lower among children whose mother used any IFA supplements during pregnancy compared to those whose mothers used no supplementation (15).

*Sanitation and Access to Safe Water*

Community-led water and sanitation efforts have been important in making improvements in access to clean water and appropriate sanitation in Nepal, which have been linked to improvements in child health over the past several decades. Between 1990 and 2012, the proportion of the population using an improved sanitation facility rose from 6% to 62% (23). This time period coincides with the ramp up of Community-Led Total Sanitation (CLTS) programs by the Government of Nepal and a range of development partners and non-governmental organizations. Beginning with a CLTS pilot in 2003, mass sensitization and behavior change activities were used to encourage communities to build and use basic toilets. This was eventually expanded and School-Led Total Sanitation (SLTS) programs began 2006 that encouraged school-based clubs to promote sanitation activities. From 2001 to 2011, the percentage of households engaging in open defecation decreased from 75.1% to 42.3% (25). As of 2015, 27 of Nepal’s then 75 (now 77) districts were declared Open Defecation Free (51). These improvements are especially important as reductions in open defecation at the village-level have be linked to improvements in HAZ, with community-toilet use showing a positive contribution to HAZ scores of 0.14 standard deviations in linear regression modeling by Cunningham et al. in 2017 (24). To a lesser degree, utilization of improved water sources has also been increasing over recent decades in Nepal, with access to piped water rising from 35.4% in 2001 to 48.3% in 2011(25).

In terms of hygiene, a 2011 longitudinal evaluation of the impact of a community-based handwashing program for infants aged 3-12 months in slums around Kathmandu by Langford et al. showed the importance of hygiene-centered programs on reducing incidence of diarrheal disease in young children. They found the intervention group saw a 41% reduction in diarrheal morbidity, and that children from intervention areas reported 3.0 episodes of diarrhea over the study period versus 4.33 episodes for the control group (p=0.049) (81). This can be seen as significant as diarrheal diseases have shown an association with stunting outcomes. In the previously mentioned Paudel et al. study, diarrhea was found to be significantly associated with stunting among the 354 Nepalese children aged 6 to 59 months included in their case control study (OR=7.46, 95% CI: 2.98, 18.65) (62).

**Immediate causes (Proximal)**

*Infection/Disease*

Illness in children can have important impacts on their growth outcomes. In a study by Panter-Brick in 1997, of 71 boys and girls aged 0-49 months in rural Nepal, children who had been ill twice to five times during the period of observation (n=14) gained significantly less weight and height than children who had no or one bout of illness (n = 17). ANOVA for weight was 809 vs 1402 g, p < 0.01; and for height was 57 vs 74 cm, p < 0.003 in those who had experienced 2-5 times versus those who had experienced illness 0-1 times, respectively (73). The importance of infection to growth outcomes was also outlined in a study by Panter-Brick et al. in 2009 (82).

Immunizations are an important protector against illness and disease at all points of life, including childhood, and can help children to avoid negative impacts to their nutritional status brought on by periods of ill health. A study by Gaire et al. in 2016 which examined 2011 DHS data through bivariate and multivariate analysis found that children who had ever received an immunization were 62% less likely to be stunted than those who never had an immunization, with an AOR = 0.38 (95% CI: 0.19, 0.76, p=0.006) for immunized children (17). Regression modeling by Shively and Sununtnasuk in 2015 found that each additional immunization a child received was correlated with a 0.02 and 0.03 standard deviation increase in HAZ in children ≥24 months and <24 months, respectively (18). Furthermore, a study of 443 children aged 6 to 36 months in the rural Terai of Eastern Nepal by Singh et al. in 2009 found that while more than 83.1% of the children had received 3 or more doses of polio and DPT vaccines, among those stunted children it was only 34.5% (71).

Though Nepal has had a National Immunization Program in place since 1979, a comparison of DHS data from 1996, 2001, 2006 and 2011 by Cunningham et al in 2016 found that that the number of children being fully vaccinated in Nepal rose from 25.9% in 1996 to 51.2% in 2011, potentially linked to an increase in access to care. This study also found through regression analysis that children receiving all their vaccinations showed a relative contribution of 0.18 standard deviations to HAZ scores (24). It is important to note that vaccination levels in Nepal continued to increase despite the decade long Maoist insurgency between 1996 and 2006. A descriptive analysis of child health indicators during the insurgency by Partap and Hill in 2012 found that tetanus vaccination coverage increased from 35% in 1990 to 81% coverage in 2008, while measles vaccine coverage at 12 months increased from 57% in 1990 to 79% in 2008. DPT3 increased to 90.7% coverage during the entire period from 1990 to 2008, and the Infant Mortality Rate declined from 99/1000 live births to 41 deaths/1000 live births of this same period (27).

*Child Characteristics*

Low birthweight has also been shown to be a risk factor for stunting in a number of studies. For example, in a cross-sectional study of children in a hill community in Nepal by Gaurav et al. in 2014, 45% of children of low birthweight were found to be stunted, compared to 39% for children with a birthweight in the normal range (49).

The impacts of antenatal micronutrient supplementation on low birthweight require further analysis. In 2008, Vaidya et al. conducted a 2-3 years’ follow up on children born to mothers included in a 2005 double-blind randomized control trial where 1200 Nepalese women received either iron and folic acid or a supplement that provided the recommended daily allowance of 15 vitamins and minerals, both during their second and third trimesters of pregnancy. Of the 917 children included (455 controls, 462 intervention), mean birthweight was only slightly greater in the micronutrient group compared to the control group, with a difference of 77 g (2810 g in the intervention group versus 2733 g in the control group; 95% CI: 24, 130). At 2.5 years of age, children in the micronutrient group weighed a mean of 10.9 kg, compared to a mean of 10.7 kg in the control group. Children in the micronutrient group also had greater measurements than controls in the circumference of the head by 2.4 mm (95% CI: 0.6, 4.3), chest by 3.2 mm (0.4–6.0), and mid-upper arm by 2.4 mm (1.1–3.7), as well as in triceps skinfold thickness by 2.0 mm (0.0–0.4) (83).

Birth order has also been raised in the literature as a factor associated with stunting in Nepal. In the 2009 study of 443 rural children by Singh et al., only 63 (14.2%) were the fourth child or more, but this group represented 50.8% of all stunted children included within the study.

*Maternal Characteristics*

Maternal body mass index (BMI) has been linked to stunting outcomes in a number of studies. For example, in the Singh et al. study in 2009, 38.1% of mothers of the 443 children were considered underweight with a BMI below 18.5, and these mothers represented 40.8% of the stunted children included in the study (71). Similarly, a study by Sharma in 2000 comparing the nutritional outcomes of households participating and not participating in a USAID-led vegetables, fruits, and cash crops (VFC) program in Western Nepal found that mother’s BMI had a positive impact on their children’s nutritional status, especially weight-for-age. They found that healthier mothers had babies who were more likely to be longer and weigh more at birth, as well as being more likely to grow faster. Mother’s age was also found to be statistically significant for HAZ of the child (0.023, p ≤ 0.10) (84).

The intergenerational impact of maternal height has been shown in several studies to have a potentially large effect on childhood stunting in Nepal. One study found that every centimeter of height increased a child’s HAZ by 0.05 standard deviations. A second study showed that the relative contribution of maternal height to changes in length-for-age (LAZ) z-scores between 1996 and 2011 was 0.03 standard deviations. A third study found a significant association between mother’s height and stunting in children, with an OR of 0.92 (CI 0.89-0.96), and a fourth found AOR = 2.52, 95% CI (1.96, 3.25), for mothers considered short (<145 cm) versus not short (≥145 cm). Lastly, a fifth study also found short maternal stature to be a significant correlate of stunting in Nepal (OR: 5.58, 95% CI: 2.52, 12.40) (24,33,36,37,40). Despite the potentially large impact of maternal height on child stunting outcomes, however, data shows that the change in maternal height within Nepal over the last several decades has been minimal, increasing from 150.5 cm in 1996 to 151.1 cm in 2011 (24).

Finally, birth spacing was also seen as an important risk factor for stunting within the literature, with an aforementioned study by Singh et al. in 2009 showing that while only 8.8% of children in their study population were born less than 24 months after their closest-in-age older sibling, this group represented 46.2% of stunted children within the study (71).

# **Supplementary Appendix 3:** Multivariable Analyses Methods

We undertook two sets of hierarchical multivariable analyses that are discussed in detail below. Using complementary approaches, each of these analyses attempts to answer the same research question i.e. what are the main predictors of change in child linear growth in Nepal during 2001-2016? The linear regression based on panel datasets uses a difference-in-difference analysis framework where time*covariable interactions are used to assess factors impacting HAZ decline. This allows the analysis of multiple years of survey data and adjusts for baseline levels of covariables and varying hypothesized growth trajectories through the interaction term. The Oaxaca-Blinder decomposition is based on the same set of individual/household level data (with ecological variables). However, by design, the decomposition only uses two survey time points in a given analysis and thus “ignores” in-between survey rounds and any intermittent fluctuations in the predictors. As has been suggested in previous decomposition analyses, we operationalize child HAZ as the linear growth outcome due to its greater statistical efficiency relative to the dichotomous child stunting variable. Each of the two multivariable regression-based analysis methods pose their own strengths and limitations – however, as sensitivity analyses, study inferences should be anchored in both and congruent findings between the methods strengthen the key messages.

Linear Multivariable Regression (Difference-in-Difference Analysis)

We undertook linear multivariable regression analyses, and included all covariables and adjustment factors as fixed effects. We added interaction terms between each potential determinant and time (i.e. time*covariable interaction terms), which signify whether a change in a proposed predictor of HAZ leads to a change in HAZ over the studied time period. The four cross-sectional surveys used in this analysis were assembled into panel datasets, and difference-in-difference (DID) analyses were used. Univariate statistics were estimated using means/standard deviations and frequencies/proportions as appropriate. We used the interaction estimators in unadjusted and adjusted regression methods to estimate the DID effect. The general model specification included an interaction term between time and the various indicators. The multivariable regression models were adjusted for child age, sex and region. Effect estimates were reported with 95% confidence intervals. All statistical analyses were performed using Stata version 14.0. The complex sampling design of DHS surveys was taken into account by using the STATA's svyset function. Standard errors were estimated using the Taylor series linearization method, which incorporates sampling weight, primary sampling unit, and stratum appropriate to the DHS sample design. 

To examine the association between HAZ and various indicators, we conducted a series of step-wise linear regression models. A hierarchical modelling approach using distal, intermediate and proximal level variables was executed as suggested by Victora 1997 (85) to generate the final multivariable models. Variables within each level were selected from our general conceptual framework as defined in Figure 9. Step 1 was a series of bivariate regressions to determine crude associations between indicators in our conceptual framework and HAZ outcome. Step 2 was to use all candidate variables for multivariable model building (i.e. with p-value ≤0.20) irrespective of their direction to move forward for multivariable modeling. Selected variables are entered into backward stepwise elimination modeling within their respective levels and those with p-values <0.15 are retained. At each step, the crude and adjusted associations between the indicator and HAZ was analyzed for statistical significance. Multicollinearity among adjustment variables was evaluated using variance inflation factors (VIF) where VIF>3 were considered suspect for collinearity.

Oaxaca-Blinder Decomposition

We also undertook the commonly used Oaxaca-Blinder decomposition methods (41,86) to assess determinants of nutritional change over time in Nepal. These methods based on individual-level data have high statistical power and have been widely used to assess nutrition determinants in low and middle income settings (41,42,87,88).

We analyzed individual-level data from four rounds of Nepal’s DHSs: 2001, 2006, 2011, and 2016. Our analysis focused on the index mother-child pair from each DHS round. Defined as the youngest child of the youngest mother in each household, selection of an index pair simplifies the model and interpretation, and is common practice in advanced analysis of DHS datasets. The total number of index pairs available from each survey were n=4143, n=3640, n=1770, and n=1815 for DHS 2001, 2006, 2011, and 2016, respectively. A flow chart outlining sample size breakdown during the index pair selection process is presented in the main paper. Given that the dietary needs/practices and growth trajectories of children in the first 1000 days of life vary notably from children beyond 2 years of age, it has been suggested that these two cohorts be analyzed separately to unmask true effects of environmental conditions and other factors on undernutrition. We conducted analyses for the entire under-5 year child population, and the <6 month, 6-23 month, and the 24-59 month child populations (89).

We used the continuous formulation of HAZ (as opposed to categorical stunting) as the dependent outcome to strengthen statistical power of the analyses. Linear least square regression models -accounting for survey design and weights - were used to assess associations between $\boldsymbol{y}_{\boldsymbol{i,t}}$, our outcome variable measured for a child *i* at time *t*, a vector of time-varying determinants (**X**), time-invariant child age and sex control variables (**C**), and a survey round time variable (**T**) to capture any trend effects. Collectively, with the standard error term, the model is expressed in Equation 1.

$\boldsymbol{Y}_{\boldsymbol{i,t}}=\boldsymbol{\beta}\boldsymbol{X}_{i,k}+\boldsymbol{C}_{i}+\boldsymbol{T} + \varepsilon_{i,t}$ [Equation 1]

Relevant individual and household level determinants in the DHS dataset were supplemented with district-level variables from the ecological dataset. When similar variables were available in both the ecological and DHS datasets, the latter were selected for analysis. The conceptual framework and corresponding list of covariables, their data sources, and definitions used in decomposition assessment are included in Supplementary Appendix 5. Applying the conceptual framework, we used a similar hierarchical modelling approach (as described for DID analysis) whereby we examined the distal, intermediate, and proximal level determinants of HAZ.

Equation 1 was applied to derive β coefficients for determinants (DHS 2001 – DHS 2016). To explain the relative contribution of each covariable over time to HAZ change, we used the Oaxaca- Blinder decomposition under the assumption that the β coefficients are the same across the two populations and the error term has the mean zero. Using the estimated parameters from Equation 1 and the (weighted) means of explanatory variables in the two time points, we applied Equation 2 (e.g. for years 2001 to 2016) to obtain the predicted change in HAZ due to the change in each determinant (90).

$$\Delta\bar{Y}_{i,t}=\beta\left( \bar{X}_{2016}-\bar{X}_{2001} \right)$$

The product coefficients for individual determinants were subsequently ranked to identify the relative contribution of each factor to HAZ change. Like determinants were also grouped into broader domains for interpretation. We examined variance inflation factors (VIF) to assess multicollinearity between variables whereby a VIF > 3 was considered suspect of high inter-variable correlation. For model building, a p-value <0.20 was considered statistically important and variables with p< 0.15 were retained in the final hierarchical multivariable models. All analyses were carried out in Stata version 14.0.

# **Supplementary Appendix 4:** Qualitative Data Collection and Analyses Methods

Qualitative Inquiry Processes

The qualitative component of the case study aimed to understand the drivers of stunting reduction among children in Nepal through exploring the perspectives of key national stakeholders in the development and implementation of relevant policies and programs, and the experiences of community health workers and mothers in the community. Specific qualitative research objectives included:

1. To explore nutrition-specific and –sensitive key events (policies/strategies/programs/guidelines) in Nepal that may have contributed to a reduction in child stunting;
2. To identify important contextual factors that have functioned as enablers/drivers and barriers to reduction of stunting in Nepal; and
3. To document community-level insight and experiences on the stunting transition in Nepal from community/volunteer health workers and mothers of children born.

The conceptual framework by Black et al. informed the development of an adapted framework (Figure 2 in paper), the design of the in-depth interview and FGD guides, as well as analysis and interpretation of the qualitative data. Our qualitative data collection tools were also informed by existing literature and nutrition questionnaires; for example, the International Food Policy Research Institute’s nutrition-focused qualitative data collection toolkit was consulted and relevant tooks were adapted to our research objectives as appropriate. Data was analyzed using key themes including: basic causes, underlying causes, and immediate causes of reduction in stunting and malnutrition.

Qualitative Research Design

We undertook three independent research activities to inform study objectives. At the first stage, national stakeholders were interviewed to provide insight and expertise on objectives 1 and 2. This top-down approach aimed to solicit macro-level perspectives and experiences in health and nutrition in Nepal. To understand how individuals in the community received and implemented major nutrition-specific and –sensitive policy/program events and their experiences in the nutritional transition as a whole, we consulted childcare workers in the community (e.g. at schools, health facilities, etc.) and the mothers of these children. These latter two research activities largely informed objectives 2 and 3, but also shed light on objective 1.

*Sampling and Recruitment Strategy*

Participants were identified and selected using purposive sampling strategies (91), including snowballing sampling (92). National stakeholders were purposively selected due to their involvement in the design, implementation, monitoring or evaluation of nutrition-specific or –sensitive policies and programs (Table 6). Key informants were asked to identify and refer the research team to other individuals with knowledge and expertise in the area of nutrition, policy, and stunting reduction. Participants were recruited by phone and a follow-up email was sent to request their participation in the study. Community health workers were purposively selected based on their experiences of working in communities for over five years, delivering primary health services, including nutrition-related services for communities. Community health workers also identified mothers of children born in 1995-2000 and between 2010-2015, using health facility records to participate in the FGDs. These sampling strategies helped to ensure that a range of diverse perspectives at national and community levels were captured.

**Supplementary** **Table 1:** Inclusion Criteria

| **Type of Stakeholder** | **Inclusion Criteria** |
| --- | --- |
| National Stakeholders | - Key informants with extensive experience in and knowledge of the design, implementation and evaluation of nutrition-specific and –sensitive policies and programs in Nepal. Examples include: national policymakers (e.g., Ministry of Health, National Planning Commission, etc.), bilateral/multilateral organizations (e.g., WHO, UNICEF, World Bank), international/local NGOs (e.g., Hellen Keller International, Project HOPE, etc.) and researchers (e.g., Patan Academic of Health Sciences, Nepal Agriculture Research Council). |
| Community Health Workers | - Unpaid/voluntary work as a community health worker in Techo or Dukuchhap for a minimum of five years. Examples include: Health workers (HW) and Female Community Health Volunteers (FCHV). |
| Mothers in Communities | - Mothers of children born in 1995-2000; - Mothers of children born between 2010-2015; and - Currently living in Dukuchhap or Techno communities, Godawari municipality, Lalitpur district. |

Lalitpur district and specific communities were purposively selected, using convenience sampling, as these represented different geographic settings and had dramatic decrease in stunting prevalence. Thecho is a semi-urban community, and rural perspectives are captured in Dukuchhap (Figures 10, 11). In addition, Province 3, where Lalitpur is located, experienced drastic declines in stunting and chronic malnutrition, as the prevalence of stunting decreased from 60% in 2001 to 31% in 2016.

## **Supplementary Figure 3A:** Location of Lalitpur district in Nepal


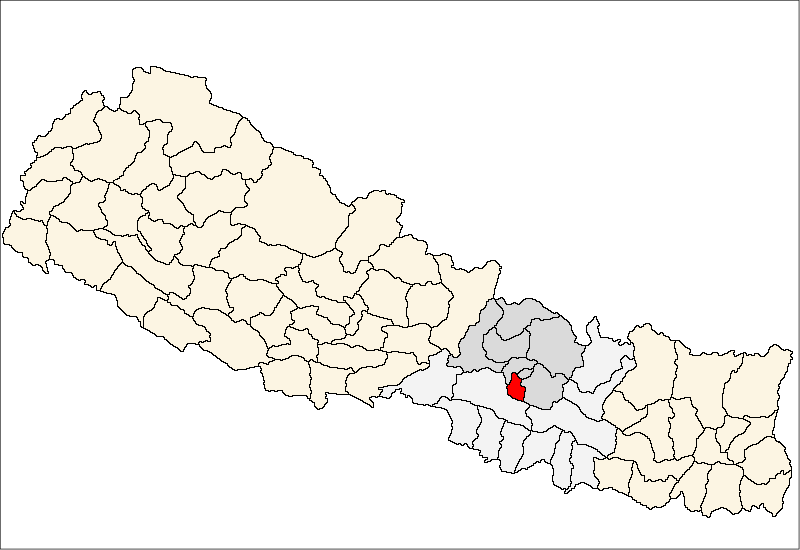


## **Supplementary Figure 3B:** Lalitpur Map displaying the study sites for FGDs and in-depth interviews


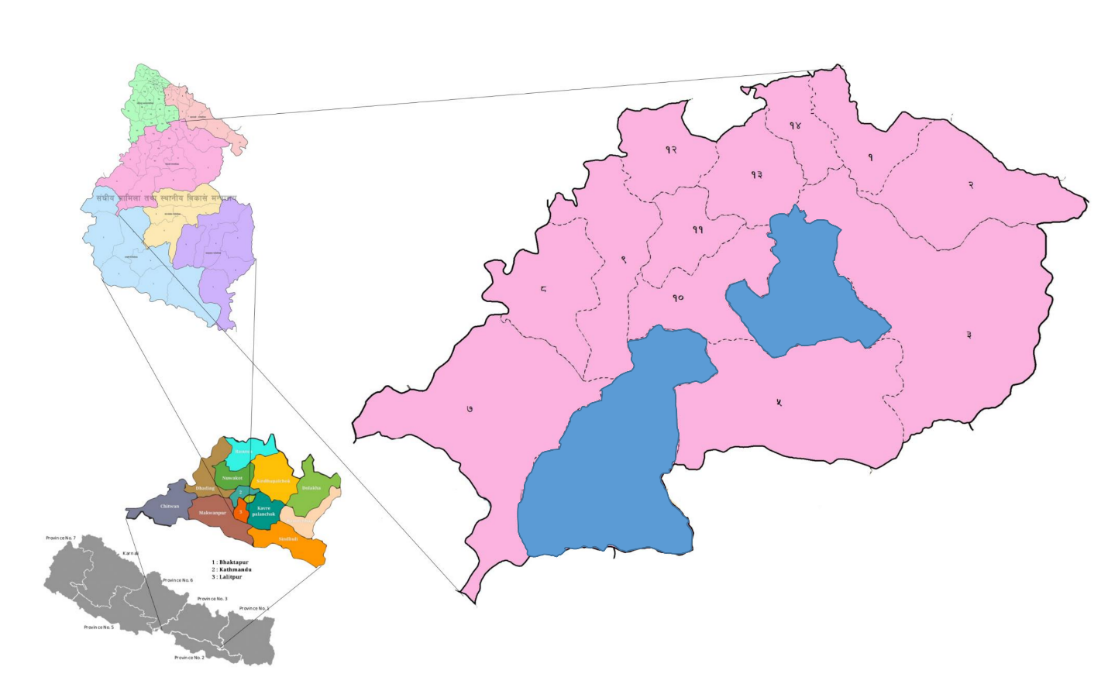


Thecho

Dukuchhap

Research Methods

Primary qualitative data collection methods involved conducting in-depth interviews (IDI) with key informants at national and community levels, and focus group discussions (FGDs) with mothers in communities. Data collection was conducted from December 2017-May 2018. Separate FGD and IDI guides for national health/nutrition experts, national policy implementers, community health workers, and mothers were developed (Appendix 6-9). Interview and FGD guides were piloted with NPHF staff with experience in nutrition and health, and changes regarding wording and prompts were made. Pilot interviews were excluded from analysis.

*In-depth interviews*

In-depth interviews were conducted with 18 key informants, including nutrition/health experts and stakeholders, to gain insight into success stories behind the stunting decline. IDIs were also conducted with community health workers to understand local perspectives regarding the improvement in nutritional status among children under-five years over time. Semi-structured interview guides were created to elicit responses detailing changes and challenges to improvements in childhood nutrition over the last 20 years at both national and community levels. All participants gave their signed consent before the interview began. An interview guide was sent to each respondent by email prior to the interview date. The interviews lasted between 40 and 90 minutes, with an average interview time of 40 minutes.

In addition, ten IDIs were conducted with community representations, including HIs/FCHVs. These were conducted in Thecho and Dukucchap communities and lasted on average 25 minutes. Community-level IDIs focused on the implementation of policies and programs that may have contributed to the reduction in chronic malnutrition and stunting. All interviews were conducted by bilingual interviewers in either English or Nepali, as preferred by the respondent. All interviews were digitally recorded and later translated and transcribed into English by the bilingual interviewer, with oversight from the research supervisor.

*Focus Group Discussions*

Two FGDs were conducted with mothers of children born between 1995-2000 and between 2010-2015. The FGDs were conducted first, with members of the research team acting as moderator and note-taker, and the IDIs were conducted second, allowing us to explore in further detail among Community Health Workers and Health-in-Charge (FCHVs/HIs) key aspects that were highlighted during the group discussions in a respective community. These were conducted in a neutral setting and lasted approximately 30 minutes with ten mothers in one and 12 in the other group. Mothers were asked to speak in their language of preference, i.e. Nepali. A moderator and note taker, each fluent in Nepali, attended both FGDs. Sessions were audiotaped and hand-written notes were taken.

*Data Analysis*

Data analysis was conducted in through an iterative process during data collection. Interviews and FGDs were transcribed and translated into English. Data was analyzed using a thematic analysis approach, which began with a careful reading and re-reading of transcripts. Once the data was reviewed and understood for context, the interview transcripts were exported manually, and the data was both deductively and inductively coded. Codes were labeled and assigned to segments of the transcripts, which provided a formal system to organize the data (93). Data coding continued until theoretical saturation was reached. All the themes identified were assembled, and grouped into broader categories and themes. A thematic chart was developed to reflect basic themes, organizing themes, and global themes and were categorized into four major levels (basic, intermediate I and II, and immediate) corresponding to our conceptual framework. From there, the global theme was developed. Review of recent literature on key nutrition-specific and -sensitive policies and programs in Nepal, and trends in nutrition and health indicators/outcomes were also synthesized to provide context and background on potential drivers of childhood stunting.

# **Supplementary Appendix 5:** Quantitative Results

## **Supplementary Figure 4A**: Spline analysis of inflection points of change in the slope of HAZ, 1996


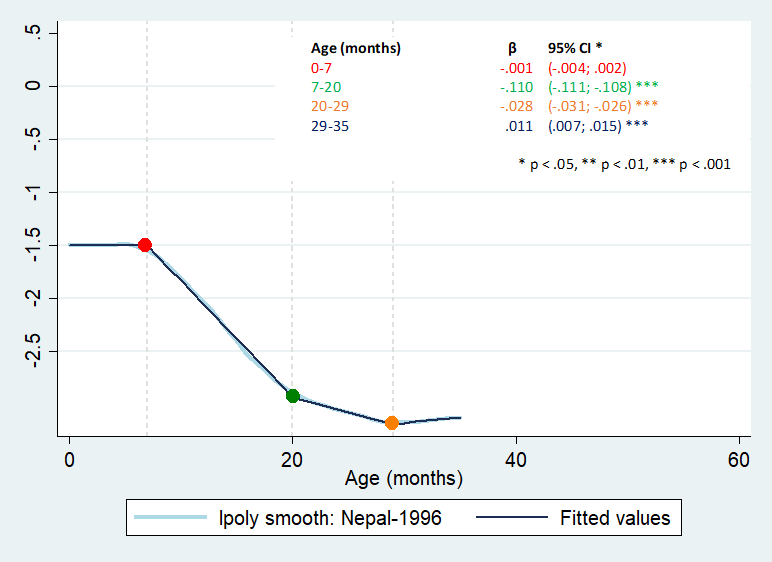


## **Supplementary Figure 4B**: Spline analysis of inflection points of change in the slope of HAZ, 2001


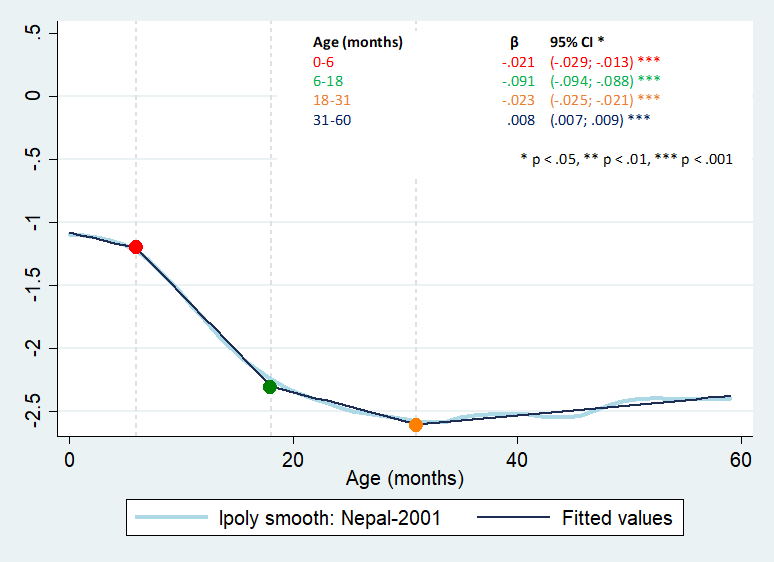


## **Supplementary Figure 4C**: Spline analysis of inflection points of change in the slope of HAZ, 2006


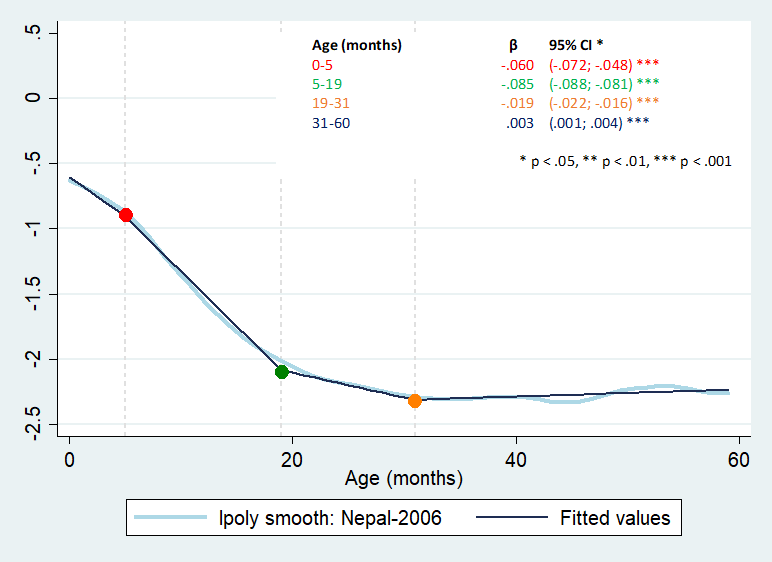


## **Supplementary Figure 4D**: Spline analysis of inflection points of change in the slope of HAZ, 2011


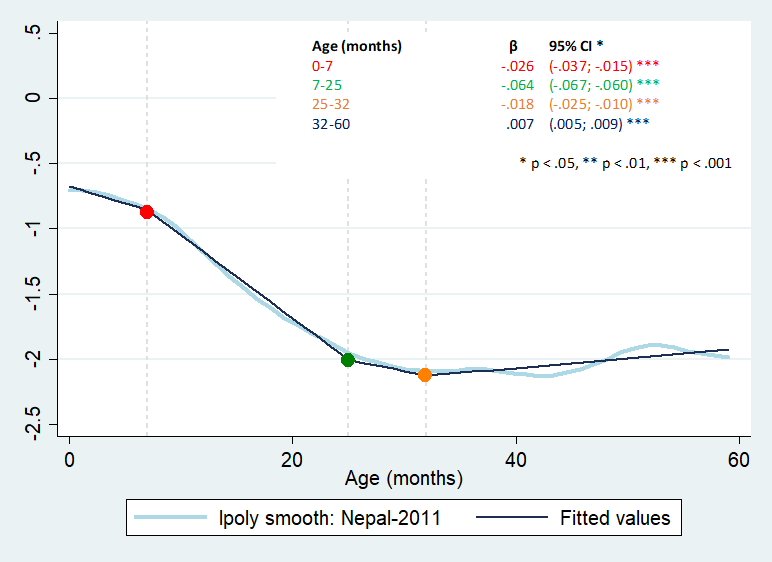


## **Supplementary Figure 4E**: Spline analysis of inflection points of change in the slope of HAZ, 2016


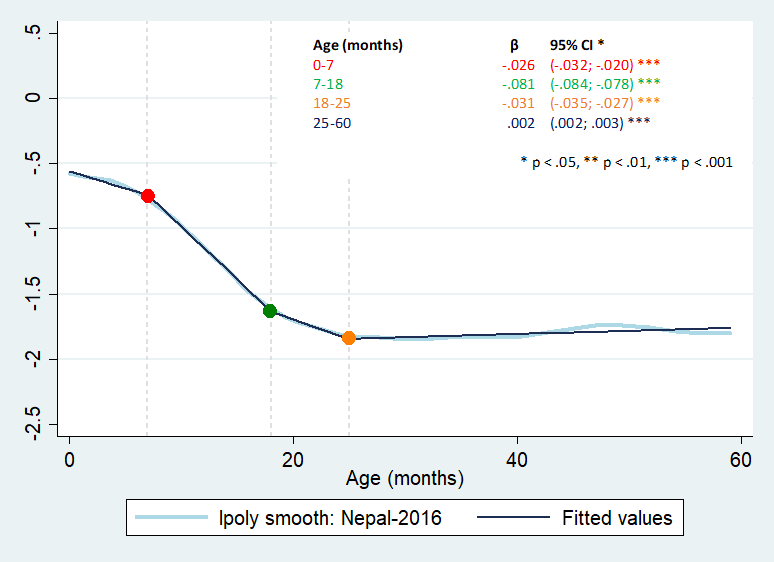


| 20-25 |  |
| --- | --- |
| 26-30 |  |
| 31-35 |  |
| 36-40 |  |
| 41-45 |  |
| 46-50 |  |
| 51-55 |  |
| 56-60 |  |
| 60+ |  |

## **Supplementary Figure 5A**: 1996 stunting prevalence by region

**Far-western (67.7)**

**Mid-western (63.3)**

**Western (66.7)**

**Central (63.9)**

**Eastern (54.4)**

## **Supplementary Figure 5B**: 2001 stunting prevalence by state

**State 1 (49.7%)**

**State 3 (59.7%)**

**State 2 (56.8%)**

**State 4 (55.8%)**

**State 5 (55.7%)**

**State 6 (70.6%)**

**State 7 (59.8%)**

## **Supplementary Figure 5C**: 2006 stunting prevalence by state

**State 1 (37.9%)**

**State 3 (46.5%)**

**State 2 (52.4%)**

**State 4 (47.3%)**

**State 5 (53.3%)**

**State 6 (62.7%)**

**State 7 (52.1%)**

## **Supplementary Figure 5D**: 2011 stunting prevalence by state

**State 1 (37.0%)**

**State 3 (33.2%)**

**State 2 (39.6%)**

**State 4 (36.4%)**

**State 5 (42.1%)**

**State 6 (55.2%)**

**State 7 (46.0%)**

## **Supplementary Figure 6A**: Change in absolute SII by year in Nepal


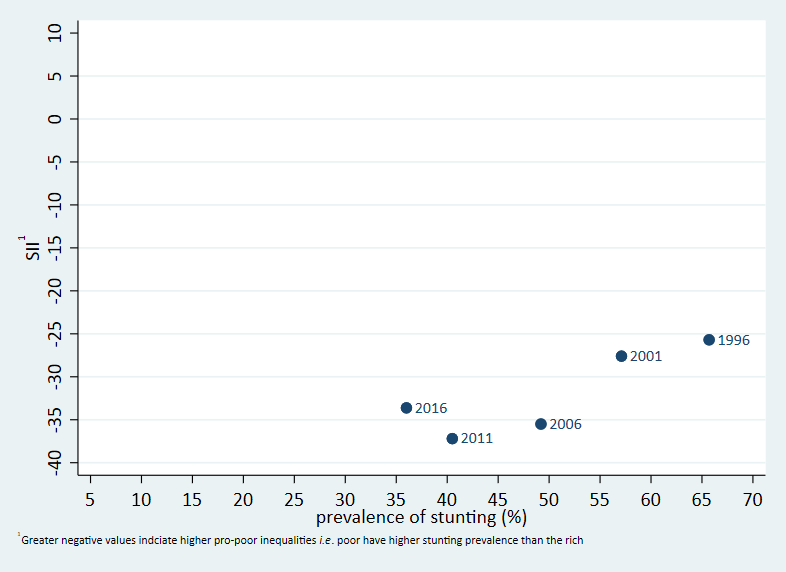


## **Supplementary Figure 6B**: Change in relative CIX by year in Nepal


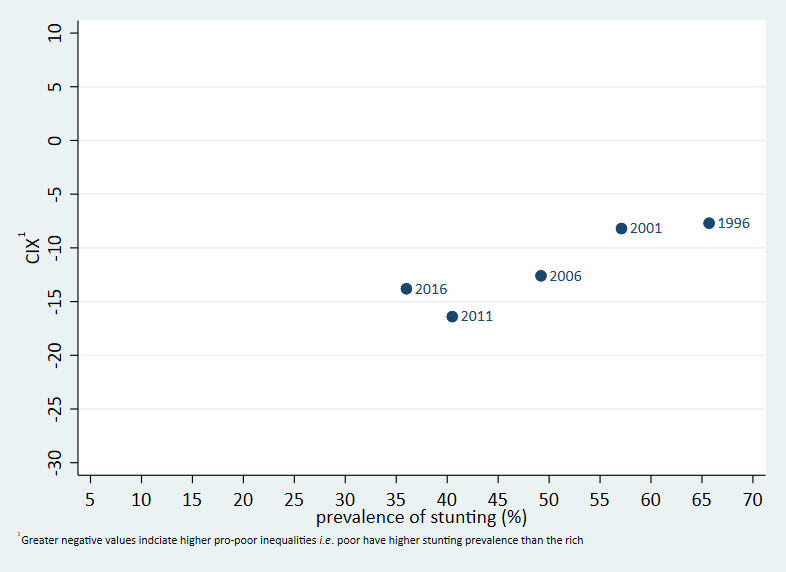


**Supplementary Figure 7:** Stunting prevalence disaggregated by child sex, 1996 – 2016


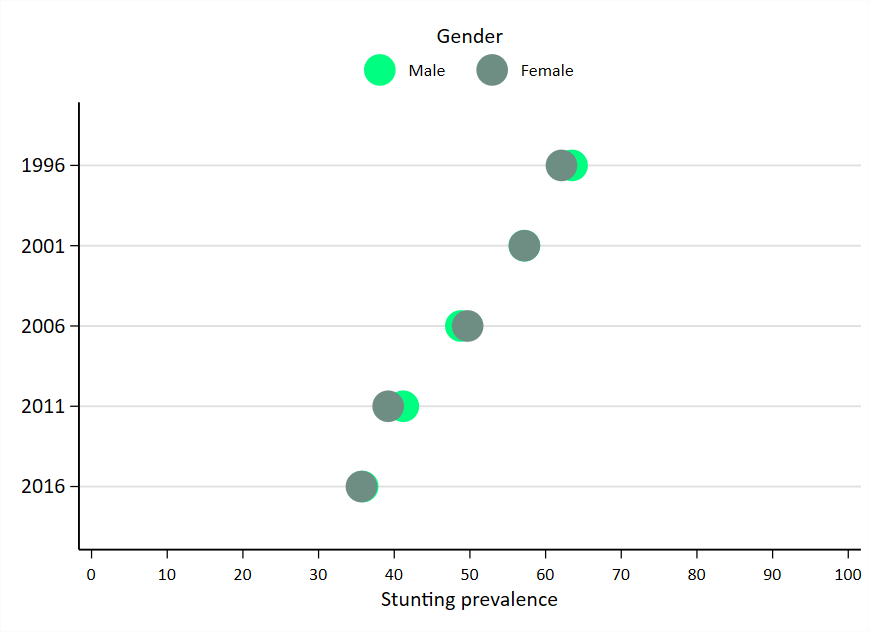


## **Supplementary Table 2**: Descriptive trends in stunting determinants in 2001-2016 in children <5 years

| **Domain /Indicator** | **DHS Survey Year** | | | | | |
| --- | --- | --- | --- | --- | --- | --- |
|  | **2001** | **2006** | **2011** | **2016** | **(2016 - 2001)** | |
|  | **(n = 4143)** | **(n = 3640)** | **(n = 1770)** | **(n = 1815)** | **Change** | **p-value** |
| **Outcome** | | | | | | |
| Height for age z-score | -2.05 | -1.78 | -1.55 | -1.41 | 0.63 | <0.001 |
|  |  |  |  |  |  |  |
|  |  |  |  |  |  |  |
| Stunting | 52.56 | 44.29 | 36.93 | 33.05 | -19.51 | <0.001 |
| % of children below -2 SD |  |  |  |  |  |  |
|  |  |  |  |  |  |  |
| **Child Demographic** | | | | | | |
|  | | | | | | |
| Child sex | 50.00 | 52.50 | 51.36 | 53.86 | 3.86 | 0.011 |
| (% males, index child) |  |  |  |  |  |  |
|  |  |  |  |  |  |  |
| Child age | 23.34 | 25.37 | 25.98 | 26.24 | 2.91 | <0.001 |
| (months, index child) |  |  |  |  |  |  |
|  |  |  |  |  |  |  |
| **Distal level** | | | | | | |
| **Basic causes & Income poverty** | | | | | | |
| Wealth Index | 4.99 | 5.32 | 5.47 | 5.43 | 0.44 | 0.020 |
| (Score 0 – 10, household) |  |  |  |  |  |  |
|  |  |  |  |  |  |  |
| Wealth Index (six components using PCA) | 3.96 | 4.87 | 4.85 | 5.07 | 1.11 | <0.001 |
| (Score 0 – 10, household) |  |  |  |  |  |  |
|  |  |  |  |  |  |  |
| Maternal education  (years of schooling) | 1.54 | 2.69 | 3.84 | 5.17 | 3.63 | <0.001 |
|  |  |  |  |  |  |  |
|  |  |  |  |  |  |  |
| Paternal education  (years of schooling) | 4.39 | 5.35 | 5.77 | 6.74 | 2.34 | <0.001 |
|  |  |  |  |  |  |  |
|  |  |  |  |  |  |  |
| **Intermediate level** | | | | | | |
| **Inadequate feeding practices and food insecurity** | | | | | | |
| Duration of breastfeeding | 20.84 | 22.14 | 19.69 | 19.39 | -1.45 | 0.002 |
| (months, index child) |  |  |  |  |  |  |
|  |  |  |  |  |  |  |
| Cluster altitude* | - | 785.78 | 633.46 | 680.55 | - | - |
| (m, cluster) |  |  |  |  |  |  |
|  |  |  |  |  |  |  |
| **Inadequate care and health services** | | | | | | |
| Live births attended by Skilled birth attendants | 13.68 | 20.82 | 42.17 | 60.23 | 46.55 | <0.001 |
| (% women) |  |  |  |  |  |  |
|  |  |  |  |  |  |  |
| Antenatal care | 14.55 | 30.46 | 48.99 | 69.32 | 54.78 | <0.001 |
| (% women with at least 4 visits) |  |  |  |  |  |  |
|  |  |  |  |  |  |  |
| New cases of diarrhea treated with Oral rehydration solution  (%) | 89.40 | 90.30 | 98.50 | 87.06 | -2.34 | 0.001 |
|  |  |  |  |  |  |  |
|  |  |  |  |  |  |  |
| Number of government hospitals  (Total, per 10,000) | 0.03 | 0.04 | 0.03 | 0.04 | 0.01 | <0.001 |
|  |  |  |  |  |  |  |
|  |  |  |  |  |  |  |
| Number of primary health care centers  (Total, per 10,000) | 0.07 | 0.07 | 0.07 | 0.07 | 0.00 | 0.429 |
|  |  |  |  |  |  |  |
|  |  |  |  |  |  |  |
| Number of health posts or lower level health facilities  (Total, per 10,000) | 1.73 | 1.58 | 1.34 | 1.50 | -0.24 | <0.001 |
|  |  |  |  |  |  |  |
|  |  |  |  |  |  |  |
| All health facilities  population | 1.84 | 1.69 | 1.45 | 1.61 | -0.23 | <0.001 |
| (HP, PHCC,  hospital) |  |  |  |  |  |  |
| (Total, per 10,000) |  |  |  |  |  |  |
| Outreach clinics  (Total, per 10,000) | 12.68 | 13.62 | 41.32 | 17.33 | 4.65 | <0.001 |
|  |  |  |  |  |  |  |
|  |  |  |  |  |  |  |
| Number of mother  group meetings  (Total, per 10,000) | 56.68 | 83.83 | 151.89 | 176.82 | 120.14 | <0.001 |
| **Unhealthy household environment** | | | | | | |
| Urban locality | 7.18 | 13.39 | 9.57 | 54.69 | 47.51 | <0.001 |
| (%, households) |  |  |  |  |  |  |
|  |  |  |  |  |  |  |
| Open defecation | 75.32 | 56.85 | 46.31 | 20.66 | -54.66 | <0.001 |
| (% population) |  |  |  |  |  |  |
|  |  |  |  |  |  |  |
| Water source -  piped | 33.51 | 36.87 | 41.43 | 47.07 | 13.56 | <0.001 |
| (% population) |  |  |  |  |  |  |
|  |  |  |  |  |  |  |
| Number of  household members  (Total, household) | 6.59 | 6.30 | 5.75 | 5.68 | -0.91 | <0.001 |
| **Proximal level** | | | | | | |
| **Disease** | | | | | | |
| ARI infection in last 2 weeks | 46.16 | 20.87 | 26.69 | 22.71 | -23.44 | <0.001 |
| (%, index child) |  |  |  |  |  |  |
|  |  |  |  |  |  |  |
| Diarrhea infection in last 2 weeks | 23.90 | 14.08 | 16.99 | 8.65 | -15.25 | <0.001 |
| (%, index child) |  |  |  |  |  |  |
|  |  |  |  |  |  |  |
| **Child characteristics** | | | | | | |
| Low birthweight* | - | 12.00 | 10.22 | 12.59 | - | - |
| (%, index child) |  |  |  |  |  |  |
|  |  |  |  |  |  |  |
| Vitamin A supplementation | - | 86.52 | 85.93 | 83.01 | - | - |
| **Maternal characteristics** | | | | | | |
| Maternal age | 27.57 | 26.85 | 26.70 | 26.37 | -1.20 | <0.001 |
| (years, mothers) |  |  |  |  |  |  |
|  |  |  |  |  |  |  |
| Adolescent birth (<18 years of age) | 8.40 | 8.86 | 10.44 | 9.90 | 1.50 | 0.090 |
| (%, mothers for index child birth) |  |  |  |  |  |  |
|  |  |  |  |  |  |  |
| Older mother birth (≥35 years) | 9.64 | 7.96 | 6.40 | 4.66 | -4.99 | <0.001 |
| (%, mothers for index child birth) |  |  |  |  |  |  |
|  |  |  |  |  |  |  |
| Anemia during pregnancy  (%, mothers) | 22.00 | 20.06 | 14.80 | 10.14 | -11.86 | <0.001 |
| Body mass index | 20.24 | 20.46 | 20.97 | 21.74 | 1.50 | <0.001 |
| (kg/m^2,^ mothers) |  |  |  |  |  |  |
| Height | 150.26 | 150.70 | 151.19 | 151.34 | 1.09 | <0.001 |
| (cm, mothers) |  |  |  |  |  |  |
|  |  |  |  |  |  |  |
| Parity | 3.36 | 2.89 | 2.59 | 2.31 | -1.05 | <0.001 |
| (Total children, mother) |  |  |  |  |  |  |
|  |  |  |  |  |  |  |
| Interpregnancy interval | 46.50 | 50.82 | 55.75 | 57.95 | 11.45 | <0.001 |
| (months, index mother) |  |  |  |  |  |  |
|  |  |  |  |  |  |  |

**Supplementary Table 3:** Decomposition analysis for children among 6 – 23 months from 2001 – 2016

| **Factors** | **Estimated coefficient** | **Mean difference (2016 - 2001)** | **Predicted change in HAZ** | **Share of predicted change in (%)** |
| --- | --- | --- | --- | --- |
| HAZ Score | - | 0.660 | 0.480 | 72.7% |
| Wealth index | 0.060 | 1.084 | 0.065 | 13.5% |
| Mother education | 0.026 | 3.722 | 0.096 | 20.1% |
| Father education | 0.043 | 2.221 | 0.095 | 19.7% |
| Skilled birth attendants | 0.189 | 0.489 | 0.093 | 19.3% |
| Use of Grains, roots, and tubers | 0.208 | 0.001 | 0.000 | 0.1% |
| Use of fruits and vegetables | 0.219 | 0.186 | 0.041 | 8.5% |
| Early age pregnancy | -0.274 | 0.027 | -0.007 | -1.5% |
| Maternal BMI (kg/m2) | 0.041 | 1.059 | 0.043 | 9.0% |
| Maternal height (cm) | 0.053 | 0.950 | 0.051 | 10.6% |
| Inter-pregnancy interval (in months) | 0.003 | 10.957 | 0.033 | 6.9% |
| Others | - | - | -0.029 | -6.1% |

## **Supplementary Figure 8:** Decomposing predicted changes in HAZ among children between 6-23 months (i.e. relative ranking of product coefficients for determinant domains) from 2001-2016

**Supplementary Table 4:** Decomposition analysis for children 24-59 months from 2001 – 2016

| **Factors** | **Estimated coefficient** | **Mean difference (2016 - 2001)** | **Predicted change in HAZ** | **Share of predicted change in (%)** |
| --- | --- | --- | --- | --- |
| HAZ Score | - | 0.746 | 0.519 | 69.6% |
| Wealth index | 0.047 | 1.177 | 0.055 | 10.6% |
| Mother education | 0.029 | 3.455 | 0.101 | 19.5% |
| Father education | 0.021 | 2.465 | 0.053 | 10.1% |
| Number of health facilities | -0.073 | -0.253 | 0.019 | 3.6% |
| Reduced open defecation | -0.159 | -0.519 | 0.083 | 15.9% |
| Water source - piped | -0.124 | 0.130 | -0.016 | -3.1% |
| Diarrhea | -0.144 | -0.111 | 0.016 | 3.1% |
| Maternal BMI (kg/m2) | 0.041 | 1.732 | 0.070 | 13.5% |
| Maternal height (cm) | 0.053 | 1.204 | 0.063 | 12.2% |
| Number of children | -0.041 | -1.159 | 0.047 | 9.1% |
| Others | - | - | 0.028 | 5.5% |

## **Supplementary Figure 9:** Decomposing predicted changes in HAZ among children between 24-59 months (i.e. relative ranking of product coefficients for determinant domains) from 2001-2016

**Supplementary Table 5:** Decomposition analysis for under-5 children from 2001-2016

| **Factors** | **Estimated coefficient** | **Mean difference (2016 - 2001)** | **Predicted change in HAZ** | **Share of predicted change in (%)** |
| --- | --- | --- | --- | --- |
| HAZ Score | - | 0.632 | 0.575 | 90.9% |
| Wealth index | 0.052 | 1.106 | 0.057 | 9.9% |
| Mother education | 0.021 | 3.625 | 0.078 | 13.5% |
| Father education | 0.034 | 2.344 | 0.079 | 13.7% |
| Duration of breastfeed | -0.035 | -1.453 | 0.051 | 8.9% |
| Antenatal care visits 4+ | 0.133 | 0.548 | 0.073 | 12.6% |
| Number of health facilities | -0.051 | -0.235 | 0.012 | 2.1% |
| Reduced open defecation | -0.142 | -0.547 | 0.078 | 13.5% |
| Maternal BMI (kg/m2) | 0.042 | 1.501 | 0.064 | 11.1% |
| Maternal height (cm) | 0.054 | 1.085 | 0.058 | 10.1% |
| Inter-pregnancy interval (in months) | 0.002 | 11.445 | 0.021 | 3.7% |
| Others | - | - | 0.005 | 0.9% |

## **Supplementary Figure 10:** Decomposing predicted changes in HAZ among children under-5 (i.e. relative ranking of product coefficients for determinant domains) from 2001-2016

**]**

**Supplementary Table 6:** Difference-in-differences multivariable regression for children under-5 years from 2001 - 2016

| **Domain/Indicator** | **Outcome = HAZ** | | | |
| --- | --- | --- | --- | --- |
|  | **(Height for age z-score for under-5 children)** | | | |
|  | **Period 2001 to 2016** | | | |
|  | **Bivariate regression coefficient** | | **Final multivariable regression coefficient*** | |
|  |  | b estimate (95% CI) |  | b estimate (95% CI) |
|  |  | *p*-value |  | *p*-value |
|  |  |  |  |  |
| **Distal level** | | | | |
| **Basic causes & Income poverty** | | | | |
| Wealth Index (six components using PCA) |  | 0.084 (0.074; 0.093) |  | 0.056 (0.046; 0.066) |
| (0 - 10)  (score 0-10, household) |  | <0.001 |  | <0.001 |
|  |  |  |  |  |
| Wealth Index # Year |  | 0.001 (-0.001; 0.002) |  |  |
|  |  | 0.579 |  | -- |
|  |  |  |  |  |
| Maternal education |  | 0.083 (0.074; 0.091) |  | 0.037 (0.028; 0.047) |
| (years of schooling) |  | <0.001 |  | <0.001 |
|  |  |  |  |  |
| Maternal education # Year |  | -0.002 (-0.004; -0.001) |  |  |
|  |  | 0.005 |  | -- |
|  |  |  |  |  |
| Paternal education |  | 0.063 (0.055; 0.071) |  | 0.024 (0.015; 0.034) |
| (years of schooling) |  | <0.001 |  | <0.001 |
|  |  |  |  |  |
| Paternal education # Year |  | -0.001 (-0.002; 0.001) |  |  |
|  |  | 0.271 |  | -- |
|  |  |  |  |  |
| **% of residual variance explained by covariates** | | |  | **18.8%** |
| **Intermediate level** | | | | |
| **Inadequate feeding practices and food insecurity** | | | | |
| Duration of breast feeding |  | -0.036 (-0.038; -0.034) |  | -0.033 (-0.038; -0.027) |
| (months, index child) |  | <0.001 |  | <0.001 |
|  |  |  |  |  |
| Duration of breast feeding # Year |  | -0.0003 (-0.0007; 0.0001) |  |  |
|  |  | 0.172 |  | -- |
|  |  |  |  |  |
| **Inadequate care and health services** | | | | |
| Skilled attendant at birth |  | 0.569 (0.493; 0.645) |  | 0.139 (0.049; 0.23) |
| (%, mothers) |  | <0.001 |  | 0.003 |
|  |  |  |  |  |
| Skilled attendant at birth # Year |  | -0.018 (-0.03; -0.005) |  |  |
|  |  | 0.006 |  | -- |
|  |  |  |  |  |
| 4+ antenatal care visits |  | 0.499 (0.422; 0.576) |  | 0.11 (0.022; 0.197) |
| (%, mothers) |  | <0.001 |  | 0.014 |
|  |  |  |  |  |
| 4+ antenatal care visits # Year |  | -0.019 (-0.033; -0.006) |  |  |
|  |  | 0.005 |  | -- |
|  |  |  |  |  |
| Number of government hospitals |  | -3.416 (-4.527; -2.306) |  |  |
| (Total, per 10,000) |  | <0.001 |  | -- |
|  |  |  |  |  |
| Number of government hospitals # Year |  | 0.0786 (-0.1582; 0.3154) |  |  |
|  |  | 0.515 |  | -- |
|  |  |  |  |  |
| Number of primary health care centers |  | -0.243 (-1.839; 1.354) |  |  |
| (Total, per 10,000) |  | 0.766 |  | -- |
|  |  |  |  |  |
| Number of primary health care centers # Year |  | -0.0277 (-0.2993; 0.2439) |  |  |
|  |  | 0.841 |  | -- |
|  |  |  |  |  |
| Number of health posts or lower level health facilities |  | -0.198 (-0.241; -0.156) |  | -0.086 (-0.135; -0.038) |
| (Total, per 10,000) |  | <0.001 |  | <0.001 |
|  |  |  |  |  |
| Number of health posts or lower level health facilities # Year |  | 0.0124 (0.0051; 0.0198) |  |  |
|  |  | 0.001 |  |  |
|  |  |  |  |  |
| Outreach clinics |  | -0.0021 (-0.0042; -0.0001) |  |  |
| (Total, per 10,000) |  | 0.044 |  | -- |
|  |  |  |  |  |
| Outreach clinics # Year |  | 0.00004 (-0.00044; 0.00053) |  |  |
|  |  | 0.86 |  | -- |
|  |  |  |  |  |
| Number of mother’s group meetings |  | -0.0005 (-0.0007; -0.0002) |  |  |
| (Total, per 10,000) |  | <0.001 |  | -- |
|  |  |  |  |  |
| Number of mother group meetings # Year |  | -0.00002 (-0.00011; 0.00008) |  |  |
|  |  | 0.728 |  | -- |
|  |  |  |  |  |
| **Unhealthy household environment** | | | | |
| Urban locality |  | 0.319 (0.228; 0.41) |  |  |
| (%, households) |  | <0.001 |  | -- |
|  |  |  |  |  |
| Urban locality # Year |  | -0.0274 (-0.0422; -0.0125) |  |  |
|  |  | <0.001 |  | -- |
|  |  |  |  |  |
| Open defecation |  | -0.411 (-0.481; -0.341) |  | -0.112 (-0.188; -0.036) |
| (%, households ) |  | <0.001 |  | 0.004 |
|  |  |  |  |  |
| Open defecation # Year |  | 0.0006 (-0.0125; 0.0136) |  |  |
|  |  | 0.934 |  | -- |
|  |  |  |  |  |
| Piped water source |  | -0.069 (-0.146; 0.009) |  | -34.985 (-60.007; -9.963) |
| (%, households) |  | 0.082 |  | 0.006 |
|  |  |  |  |  |
| Piped water source # Year |  | 0.0138 (0.0008; 0.0268) |  | 0.017 (0.005; 0.03) |
|  |  | 0.037 |  | 0.006 |
|  |  |  |  |  |
| Number of household members |  | 0.016 (0.004; 0.028) |  |  |
|  |  | 0.011 |  | -- |
|  |  |  |  |  |
| Number of household members # Year |  | -0.0026 (-0.0048; -0.0004) |  |  |
|  |  | 0.02 |  | -- |
|  |  |  |  |  |
| **% of residual variance explained by covariates** | | |  | **22.1%** |
| **Proximal level** | | | | |
| **Disease** | | | | |
| ARI infection in last 2 weeks |  | 0.153 (0.086; 0.221) |  |  |
| (%, index child) |  | <0.001 |  | -- |
|  |  |  |  |  |
| ARI infection # Year |  | -0.005 (-0.017; 0.006) |  |  |
|  |  | 0.38 |  | -- |
|  |  |  |  |  |
| Diarrhea infection in last 2 weeks |  | -0.0411 (-0.1198; 0.0376) |  |  |
| (%, index child) |  | 0.306 |  | -- |
|  |  |  |  |  |
| Diarrhea infection # Year |  | 0.004 (-0.011; 0.019) |  |  |
|  |  | 0.597 |  | -- |
|  |  |  |  |  |
| **Maternal characteristics** | | | | |
| Age |  | -0.034 (-0.039; -0.03) |  | 0.007 (-0.001; 0.014) |
| (Mean, mothers 15-49) |  | <0.001 |  | 0.069 |
|  |  |  |  |  |
| Age # Year |  | 0.00029 (-0.00056; 0.00115) |  |  |
|  |  | 0.499 |  | -- |
|  |  |  |  |  |
| Adolescent birth (<18 years of age) |  | -0.012 (-0.113; 0.089) |  |  |
| (% mothers for index child within last 5 years) |  | 0.822 |  | -- |
|  |  |  |  |  |
| Adolescent birth # Year |  | -0.009 (-0.027; 0.009) |  |  |
|  |  | 0.321 |  | -- |
|  |  |  |  |  |
| Older mother birth (≥35 years) |  | -0.414 (-0.53; -0.298) |  |  |
| (% mothers for index child within last 5 years) |  | <0.001 |  | -- |
|  |  |  |  |  |
| Older mother birth # Year |  | -0.012 (-0.036; 0.011) |  |  |
|  |  | 0.301 |  | -- |
|  |  |  |  |  |
| Body mass index |  | 0.057 (0.047; 0.068) |  | 0.046 (0.035; 0.057) |
| (kg/m2, mothers) |  | <0.001 |  | <0.001 |
|  |  |  |  |  |
| Body mass index # Year |  | 0.057 (0.047; 0.068) |  |  |
|  |  | <0.001 |  | -- |
|  |  |  |  |  |
| Height |  | 0.059 (0.053; 0.065) |  | 0.056 (0.05; 0.061) |
| (cm, mothers) |  | <0.001 |  | <0.001 |
|  |  |  |  |  |
| Height # Year |  | 0 (-0.001; 0.001) |  |  |
|  |  | 0.998 |  | -- |
|  |  |  |  |  |
| Parity |  | -0.118 (-0.133; -0.103) |  |  |
| (Total children, mother) |  | <0.001 |  | -- |
|  |  |  |  |  |
| Parity # Year |  | -0.004 (-0.007; -0.001) |  |  |
|  |  | 0.02 |  | -- |
|  |  |  |  |  |
| Interpregnancy interval |  | 0.006 (0.005; 0.007) |  |  |
| (months, index mother) |  | <0.001 |  | -- |
|  |  |  |  |  |
| Interpregnancy interval # Year |  | 0.00006 (-0.00016; 0.00028) |  |  |
|  |  | 0.606 |  | -- |
|  |  |  |  |  |
| **% of residual variance explained by covariates** | | |  | **27.2%** |
| **Time** | | | | |
| **Time** | | | | |
| Year |  | 0.044 (0.037; 0.051) |  | 0.01 (0.001; 0.018) |
|  |  | <0.001 |  | 0.024 |
|  |  |  |  |  |
| * Adjusted for child age, sex, and state |  |  |  |  |

**Supplementary Table 7:** Difference-in-differences multivariable regression for children 24-59 months from 2001 – 2016

| **Domain/Indicator** | **Outcome = HAZ** | | | |
| --- | --- | --- | --- | --- |
|  | **(Height for age z-score for 24 and above children)** | | | |
|  | **Period 2001 to 2016** | | | |
|  | **Bivariate regression coefficient** | | **Final multivariable regression coefficient*** | |
|  |  | b estimate (95% CI) |  | b estimate (95% CI) |
|  |  | *p*-value |  | *p*-value |
|  |  |  |  |  |
| **Distal level** | | | | |
| **Basic causes & Income poverty** | | | | |
| Wealth Index (six components using PCA) |  | 0.079 (0.067; 0.091) |  | 0.052 (0.038; 0.066) |
| (0 - 10)  (score 0-10, household) |  | <0.001 |  | <0.001 |
|  |  |  |  |  |
| Wealth Index # Year |  | 0.001 (-0.001; 0.003) |  |  |
| (0 - 10) |  | 0.471 |  | -- |
|  |  |  |  |  |
| Maternal education |  | 0.001 (-0.001; 0.003) |  | 0.037 (0.024; 0.051) |
| (years of schooling) |  | 0.471 |  | <0.001 |
|  |  |  |  |  |
| Maternal education # year |  | -0.0023 (-0.0041; -0.0005) |  |  |
|  |  | 0.014 |  | -- |
|  |  |  |  |  |
| Paternal education |  | 0.052 (0.043; 0.062) |  | 0.015 (0.003; 0.026) |
| (years of schooling) |  | <0.001 |  | 0.01 |
|  |  |  |  |  |
| Paternal education # Year |  | -0.001 (-0.003; 0.001) |  |  |
|  |  | 0.261 |  | -- |
|  |  |  |  |  |
| **% of residual variance explained by covariates** | | |  | **12.2%** |
| **Intermediate level** | | | | |
| **Inadequate feeding practices and food insecurity** | | | | |
| Duration of breast feeding |  | -0.012 (-0.016; -0.007) |  |  |
| (months, index child) |  | <0.001 |  | -- |
|  |  |  |  |  |
| Duration of breast feeding # Year |  | -0.0004 (-0.0013; 0.0005) |  |  |
|  |  | 0.369 |  | -- |
|  |  |  |  |  |
| **Inadequate care and health services** | | | | |
| Skilled attendant at birth |  | 0.464 (0.367; 0.56) |  | 0.099 (-0.005; 0.203) |
| (%, mothers) |  | <0.001 |  | 0.063 |
|  |  |  |  |  |
| Skilled attendant at birth # Year |  | -0.018 (-0.034; -0.003) |  |  |
|  |  | 0.023 |  | -- |
|  |  |  |  |  |
| 4+ antenatal care visits |  | 0.411 (0.312; 0.51) |  |  |
| (%, mothers) |  | <0.001 |  |  |
|  |  |  |  |  |
| 4+ antenatal care visits # Year |  | -0.023 (-0.039; -0.006) |  |  |
|  |  | 0.007 |  | -- |
|  |  |  |  |  |
| Number of government hospitals |  | -2.84 (-4.14; -1.54) |  |  |
| (Total, per 10,000) |  | <0.001 |  | -- |
|  |  |  |  |  |
| Number of government hospitals # Year |  | -0.1468 (-0.4123; 0.1186) |  |  |
|  |  | 0.278 |  | -- |
|  |  |  |  |  |
| Number of primary health care centers |  | -1.043 (-2.772; 0.685) |  |  |
| (Total, per 10,000) |  | 0.236 |  | -- |
|  |  |  |  |  |
| Number of primary health care centers # Year |  | -0.1879 (-0.481; 0.1052) |  |  |
|  |  | 0.209 |  | -- |
|  |  |  |  |  |
| Number of health posts or lower level health facilities |  | -0.222 (-0.273; -0.171) |  | -15.169 (-29.266; -1.071) |
| (Total, per 10,000) |  | <0.001 |  | 0.035 |
|  |  |  |  |  |
| Number of health posts or lower level health facilities # Year |  | 0.0091 (0.0012; 0.017) |  | 0.0075 (0.0005; 0.0145) |
|  |  | 0.024 |  | 0.036 |
|  |  |  |  |  |
| Outreach clinics |  | -0.003 (-0.0055; -0.0004) |  |  |
| (Total, per 10,000) |  | 0.023 |  | -- |
|  |  |  |  |  |
| Outreach clinics # Year |  | -0.00026 (-0.00086; 0.00034) |  |  |
|  |  | 0.394 |  | -- |
|  |  |  |  |  |
| Number of mother group meetings |  | -0.0006 (-0.0009; -0.0002) |  |  |
| (Total, per 10,000) |  | 0.002 |  | -- |
|  |  |  |  |  |
| Number of mother group meetings # Year |  | -0.00004 (-0.00014; 0.00007) |  |  |
|  |  | 0.489 |  | -- |
|  |  |  |  |  |
| **Unhealthy household environment** | | | | |
| Urban locality |  | 0.304 (0.201; 0.408) |  |  |
| (%, households) |  | <0.001 |  | -- |
|  |  |  |  |  |
| Urban locality # Year |  | -0.0241 (-0.0412; -0.0071) |  |  |
|  |  | 0.006 |  | -- |
|  |  |  |  |  |
| Open defecation |  | -0.417 (-0.502; -0.333) |  | -0.122 (-0.213; -0.031) |
| (%, households ) |  | <0.001 |  | 0.009 |
|  |  |  |  |  |
| Open defecation # Year |  | 0.015 (-0.0009; 0.0308) |  |  |
|  |  | 0.064 |  | -- |
|  |  |  |  |  |
| Piped water source |  | -0.065 (-0.161; 0.03) |  | -0.11 (-0.209; -0.011) |
| (%, households) |  | 0.18 |  | 0.029 |
|  |  |  |  |  |
| Piped water source # Year |  | 0.0075 (-0.0082; 0.0233) |  |  |
|  |  | 0.346 |  | -- |
|  |  |  |  |  |
| Number of household members |  | -0.013 (-0.036; 0.009) |  |  |
|  |  | 0.236 |  | -- |
|  |  |  |  |  |
| Number of household members # Year |  | -0.0035 (-0.0067; -0.0004) |  |  |
|  |  | 0.028 |  | -- |
|  |  |  |  |  |
| **% of residual variance explained by covariates** | | |  | **13.7%** |
| **Proximal level** | | | | |
| **Disease** | | | | |
| ARI infection in last 2 weeks |  | 0.111 (0.024; 0.198) |  |  |
| (%, index child) |  | 0.013 |  | -- |
|  |  |  |  |  |
| ARI infection # Year |  | -0.001 (-0.016; 0.014) |  |  |
|  |  | 0.895 |  | -- |
|  |  |  |  |  |
| Diarrhea infection in last 2 weeks |  | -0.1844 (-0.2898; -0.079) |  | -0.147 (-0.249; -0.045) |
| (%, index child) |  | 0.001 |  | 0.005 |
|  |  |  |  |  |
| Diarrhea infection # Year |  | -0.001 (-0.021; 0.019) |  |  |
|  |  | 0.959 |  | -- |
|  |  |  |  |  |
| **Maternal characteristics** | | | | |
| Maternal age |  | -0.017 (-0.024; -0.01) |  | 0.012 (0.003; 0.021) |
| (years, mothers) |  | <0.001 |  | 0.011 |
|  |  |  |  |  |
| Age# Year |  | 0.00046 (-0.00075; 0.00167) |  |  |
|  |  | 0.455 |  | -- |
|  |  |  |  |  |
| Adolescent birth (<18 years of age) |  | 0.019 (-0.14; 0.178) |  |  |
| (% mothers for index child within last 5 years) |  | 0.814 |  | -- |
|  |  |  |  |  |
| Adolescent birth # Year |  | -0.011 (-0.035; 0.013) |  |  |
|  |  | 0.35 |  | -- |
|  |  |  |  |  |
| Older mother birth (≥35 years) |  | -0.314 (-0.474; -0.154) |  |  |
| (% mothers for index child within last 5 years) |  | <0.001 |  | -- |
|  |  |  |  |  |
| Older mother birth # Year |  | 0.001 (-0.031; 0.034) |  |  |
|  |  | 0.939 |  | -- |
|  |  |  |  |  |
| Body mass index |  | 0.061 (0.048; 0.075) |  | 0.043 (0.031; 0.056) |
| (kg/m2, mothers) |  | <0.001 |  | <0.001 |
|  |  |  |  |  |
| Body mass index # Year |  | 0.001 (-0.001; 0.003) |  |  |
|  |  | 0.327 |  | -- |
|  |  |  |  |  |
| Height |  | 0.059 (0.051; 0.066) |  | 0.056 (0.049; 0.063) |
| (cm, mothers) |  | <0.001 |  | <0.001 |
|  |  |  |  |  |
| Height # Year |  | 0.0001 (-0.0012; 0.0014) |  |  |
|  |  | 0.853 |  | -- |
|  |  |  |  |  |
| Parity |  | -0.096 (-0.117; -0.075) |  | -0.061 (-0.09; -0.032) |
| (Total children, mother) |  | <0.001 |  | <0.001 |
|  |  |  |  |  |
| Parity # Year |  | -0.002 (-0.006; 0.002) |  |  |
|  |  | 0.275 |  | -- |
|  |  |  |  |  |
| Interpregnancy interval |  | 0.005 (0.004; 0.007) |  | 0.0017 (0.0002; 0.0032) |
| (months, index mother) |  | <0.001 |  | 0.026 |
|  |  |  |  |  |
| Interpregnancy interval # Year |  | -0.00005 (-0.00032; 0.00022) |  |  |
|  |  | 0.733 |  | -- |
|  |  |  |  |  |
| **% of residual variance explained by covariates** | | |  | **21.0%** |
| **Time** | | | | |
| **Time** | | | | |
| Year |  | 0.051 (0.043; 0.058) |  | 0.005 (-0.008; 0.019) |
|  |  | <0.001 |  | 0.432 |
| * Adjusted for child age, sex, and state |  |  |  |  |

*# indicates time*covariate interaction terms*

*Notes: Variables significant at p<0.20 in bivariate analysis were entered into linear models.*

*^a^Level 3 multivariable model includes all statistically significant (p<0.15) distal variables as listed.*

*^b^Level 2 multivariable model includes level 3 model all statistically significant (p<0.15) intermediate variables as listed.*

*^c^Level 1 multivariable model includes level 3 model + 2 model + all statistically significant (p<0.15) proximal variables as listed*

## **Supplementary Table 8:** Difference-in-differences multivariable regression for children 6-23 months old using DHS surveys from 2001 – 2016

| **Domain/Indicator** | **Outcome = HAZ** | | | |
| --- | --- | --- | --- | --- |
|  | **(Height for age z-score among 6 to 23 months children)** | | | |
|  | **Period 2001 to 2016** | | | |
|  | **Bivariate regression coefficient** | | **Final multivariable regression coefficient*** | |
|  |  | b estimate (95% CI) |  | b estimate (95% CI) |
|  |  | *p*-value |  | *p*-value |
|  |  |  |  |  |
| **Distal level** | | | | |
| **Basic causes & Income poverty** | | | | |
| Wealth Index (six components using PCA) |  | 0.1 (0.084; 0.116) |  | 0.062 (0.047; 0.077) |
| (0 - 10)  (score 0-10, household) |  | <0.001 |  | <0.001 |
|  |  |  |  |  |
| Wealth Index # Year |  | 0.0004 (-0.0026; 0.0034) |  |  |
|  |  | 0.802 |  | -- |
|  |  |  |  |  |
| Maternal education |  | 0.088 (0.074; 0.101) |  | 0.039 (0.024; 0.054) |
| (years of schooling) |  | <0.001 |  | <0.001 |
|  |  |  |  |  |
| Maternal education # Year |  | -0.0025 (-0.0048; -0.0003) |  |  |
|  |  | 0.03 |  | -- |
|  |  |  |  |  |
| Paternal education |  | 0.076 (0.064; 0.089) |  | 0.034 (0.02; 0.048) |
| (years of schooling) |  | <0.001 |  | <0.001 |
|  |  |  |  |  |
| Paternal education # Year |  | -0.001 (-0.004; 0.001) |  |  |
|  |  | 0.302 |  | -- |
|  |  |  |  |  |
| **% of residual variance explained by covariates** | | |  | **22.3%** |
| **Intermediate level** | | | | |
| **Inadequate feeding practices and food insecurity** | | | | |
| Duration of breast feeding |  | -0.08 (-0.088; -0.071) |  |  |
| (months, index child) |  | <0.001 |  | -- |
|  |  |  |  |  |
| Duration of breast feeding # Year |  | 0.0001 (-0.0015; 0.0016) |  |  |
|  |  | 0.946 |  | -- |
|  |  |  |  |  |
| Complementary feeding |  | -0.102 (-0.25; 0.046) |  | 0.166 (0.03; 0.302) |
| (% children) |  | 0.176 |  | 0.017 |
|  |  |  |  |  |
| Complementary feeding # Year |  | 0.03 (0.003; 0.056) |  |  |
|  |  | 0.028 |  | -- |
|  |  |  |  |  |
| **Inadequate care and health services** | | | | |
| DPT vaccine [3 doses] |  | 0.4 (0.231; 0.568) |  |  |
| (%, index child) |  | <0.001 |  | -- |
|  |  |  |  |  |
| DPT vaccine # Year |  | -0.001 (-0.038; 0.036) |  |  |
|  |  | 0.956 |  | -- |
|  |  |  |  |  |
| Skilled attendant at birth |  | 0.635 (0.513; 0.758) |  | 0.13 (0.01; 0.25) |
| (%, mothers) |  | <0.001 |  | 0.034 |
|  |  |  |  |  |
| Skilled attendant at birth # Year |  | -0.018 (-0.039; 0.003) |  |  |
|  |  | 0.089 |  | -- |
|  |  |  |  |  |
| 4+ antenatal care visits |  | 0.598 (0.482; 0.713) |  | 0.162 (0.047; 0.277) |
| (%, mothers) |  | <0.001 |  | 0.006 |
|  |  |  |  |  |
| 4+ antenatal care visits # Year |  | -0.026 (-0.046; -0.006) |  |  |
|  |  | 0.01 |  | -- |
|  |  |  |  |  |
| Number of government hospitals |  | -3.43 (-5.116; -1.743) |  |  |
| (Total, per 10,000) |  | <0.001 |  | -- |
|  |  |  |  |  |
| Number of government hospitals # Year |  | 0.1756 (-0.2055; 0.5567) |  |  |
|  |  | 0.366 |  | -- |
|  |  |  |  |  |
| Number of primary health care centers |  | 0.418 (-1.703; 2.539) |  |  |
| (Total, per 10,000) |  | 0.699 |  | -- |
|  |  |  |  |  |
| Number of primary health care centers # Year |  | -0.0357 (-0.4498; 0.3784) |  |  |
|  |  | 0.866 |  | -- |
|  |  |  |  |  |
| Number of health posts or lower level health facilities |  | -0.198 (-0.262; -0.134) |  | -0.074 (-0.135; -0.013) |
| (Total, per 10,000) |  | <0.001 |  | 0.017 |
|  |  |  |  |  |
| Number of health posts or lower level health facilities # Year |  | 0.0114 (-0.0005; 0.0232) |  | -- |
|  |  | 0.06 |  |  |
|  |  |  |  |  |
| Outreach clinics |  | -0.0009 (-0.0043; 0.0024) |  |  |
| (Total, per 10,000) |  | 0.582 |  | -- |
|  |  |  |  |  |
| Outreach clinics # Year |  | 0.00023 (-0.0005; 0.00096) |  |  |
|  |  | 0.534 |  | -- |
|  |  |  |  |  |
| Number of mother group meetings |  | -0.0006 (-0.001; -0.0003) |  |  |
| (Total, per 10,000) |  | 0.001 |  | -- |
|  |  |  |  |  |
| Number of mother group meetings # Year |  | -0.00004 (-0.00017; 0.00009) |  |  |
|  |  | 0.551 |  | -- |
|  |  |  |  |  |
| **Unhealthy household environment** | | | | |
| Urban locality |  | 0.425 (0.268; 0.582) |  |  |
| (%, households) |  | <0.001 |  | -- |
|  |  |  |  |  |
| Urban locality # Year |  | -0.0495 (-0.0739; -0.0252) |  |  |
|  |  | <0.001 |  | -- |
|  |  |  |  |  |
| Open defecation |  | -0.51 (-0.63; -0.391) |  |  |
| (%, households ) |  | <0.001 |  | -- |
|  |  |  |  |  |
| Open defecation # Year |  | 0.003 (-0.0187; 0.0246) |  |  |
|  |  | 0.787 |  | -- |
|  |  |  |  |  |
| Piped water source |  | -0.032 (-0.154; 0.089) |  |  |
| (%, households) |  | 0.6 |  | -- |
|  |  |  |  |  |
| Piped water source # Year |  | 0.0085 (-0.0124; 0.0294) |  |  |
|  |  | 0.426 |  | -- |
|  |  |  |  |  |
| Number of household members |  | 0.0085 (-0.0124; 0.0294) |  | -0.013 (-0.028; 0.001) |
|  |  | 0.426 |  | 0.068 |
|  |  |  |  |  |
| Number of household members # Year |  | -0.002 (-0.0049; 0.0009) |  |  |
|  |  | 0.176 |  | -- |
|  |  |  |  |  |
| **% of residual variance explained by covariates** | | |  | **23.1%** |
| **Proximal level** | | | | |
| **Disease** | | | | |
| ARI infection in last 2 weeks |  | 0.165 (0.063; 0.268) |  |  |
| (%, index child) |  | 0.002 |  | -- |
|  |  |  |  |  |
| ARI infection # Year |  | 0.009 (-0.01; 0.028) |  |  |
|  |  | 0.364 |  | -- |
|  |  |  |  |  |
| Diarrhea infection in last 2 weeks |  | -0.059 (-0.173; 0.055) |  |  |
| (%, index child) |  | 0.309 |  | -- |
|  |  |  |  |  |
| Diarrhea infection # Year |  | -0.0003 (-0.0231; 0.0225) |  |  |
|  |  | 0.979 |  | -- |
|  |  |  |  |  |
| **Inadequate dietary intake** | | | | |
| Infant and young child minimum dietary diversity |  | 0.265 (0.164; 0.366) |  | 0.087 (-0.014; 0.188) |
| (% children took at least 4 food groups) |  | <0.001 |  | 0.092 |
|  |  |  |  |  |
| Infant and young child minimum dietary diversity # Year |  | -0.014 (-0.033; 0.004) |  |  |
|  |  | 0.136 |  | -- |
|  |  |  |  |  |
| Intake of grains, roots, and tubers |  | -0.372 (-0.511; -0.233) |  |  |
| (% children) |  | <0.001 |  | -- |
|  |  |  |  |  |
| Intake of grains, roots, and tubers # Year |  | 0.018 (-0.01; 0.046) |  |  |
|  |  | 0.217 |  | -- |
|  |  |  |  |  |
| Intake of legumes and nuts |  | 0.047 (-0.05; 0.145) |  |  |
| (% children) |  | 0.34 |  | -- |
|  |  |  |  |  |
| Intake of legumes and nuts # Year |  | -0.017 (-0.034; 0.001) |  |  |
|  |  | 0.063 |  | -- |
|  |  |  |  |  |
| Intake of dairy products |  | 0.209 (0.099; 0.32) |  |  |
| (% children) |  | <0.001 |  | -- |
|  |  |  |  |  |
| Intake of dairy products # Year |  | -0.0001 (-0.0197; 0.0194) |  |  |
|  |  | 0.988 |  | -- |
|  |  |  |  |  |
| Intake of flesh foods and eggs |  | -0.023 (-0.139; 0.092) |  |  |
| (% children) |  | 0.692 |  | -- |
|  |  |  |  |  |
| Intake of flesh foods and eggs # Year |  | -0.005 (-0.026; 0.015) |  |  |
|  |  | 0.605 |  | -- |
|  |  |  |  |  |
| Intake of vitamin-A rich fruits and vegetables |  | -0.066 (-0.17; 0.039) |  |  |
| (% children) |  | 0.217 |  | -- |
|  |  |  |  |  |
| Intake of vitamin-A rich fruits and vegetables # Year |  | -0.011 (-0.03; 0.008) |  |  |
|  |  | 0.255 |  | -- |
|  |  |  |  |  |
| Intake of other fruits and vegetables |  | 0.304 (0.19; 0.418) |  | 0.145 (0.033; 0.256) |
| (% children) |  | <0.001 |  | 0.011 |
|  |  |  |  |  |
| Intake of other fruits and vegetables # Year |  | -0.007 (-0.028; 0.013) |  |  |
|  |  | 0.479 |  | -- |
|  |  |  |  |  |
| **Maternal characteristics** | | | | |
| Maternal age |  | -0.023 (-0.03; -0.015) |  |  |
| (years, mothers) |  | <0.001 |  | -- |
|  |  |  |  |  |
| Age # Year |  | -0.00021 (-0.00169; 0.00127) |  |  |
|  |  | 0.78 |  | -- |
|  |  |  |  |  |
| Adolescent birth (<18 years of age) |  | -0.114 (-0.264; 0.037) |  | -0.223 (-0.365; -0.081) |
| (% mothers for index child within last 5 years) |  | 0.138 |  | 0.002 |
|  |  |  |  |  |
| Adolescent birth # Year |  | 0.005 (-0.023; 0.032) |  |  |
|  |  | 0.723 |  | -- |
|  |  |  |  |  |
| Older mother birth (≥35 years) |  | -0.376 (-0.559; -0.194) |  |  |
| (% mothers for index child within last 5 years) |  | <0.001 |  | -- |
|  |  |  |  |  |
| Older mother birth # Year |  | -0.023 (-0.059; 0.013) |  |  |
|  |  | 0.213 |  | -- |
|  |  |  |  |  |
| Body mass index |  | 0.068 (0.047; 0.09) |  | 0.046 (0.027; 0.065) |
| (kg/m2, mothers) |  | <0.001 |  | <0.001 |
|  |  |  |  |  |
| Body mass index # Year |  | 0.001 (-0.003; 0.005) |  |  |
|  |  | 0.608 |  | -- |
|  |  |  |  |  |
| Height |  | 0.065 (0.055; 0.075) |  | 0.055 (0.046; 0.064) |
| (cm, mothers) |  | <0.001 |  | <0.001 |
|  |  |  |  |  |
| Height # Year |  | 0 (-0.0018; 0.0018) |  |  |
|  |  | 0.977 |  | -- |
|  |  |  |  |  |
| Parity |  | -0.118 (-0.142; -0.093) |  | -0.0269 (-0.0535; -0.0003) |
| (Total children, mother) |  | <0.001 |  | 0.047 |
|  |  |  |  |  |
| Parity # Year |  | -0.0049 (-0.0106; 0.0007) |  |  |
|  |  | 0.087 |  | -- |
|  |  |  |  |  |
| Interpregnancy interval |  | 0.007 (0.005; 0.008) |  | 0.0017 (-0.0001; 0.0036) |
| (months, index mother) |  | <0.001 |  | 0.066 |
|  |  |  |  |  |
| Interpregnancy interval # Year |  | 0.00033 (-0.00002; 0.00067) |  |  |
|  |  | 0.063 |  | -- |
|  |  |  |  |  |
| **% of residual variance explained by covariates** | | |  | **28.6%** |
| **Time** | | | | |
| **Time** | | | | |
| Year |  | 0.05 (0.038; 0.061) |  | 0.019 (0.009; 0.029) |
|  |  | <0.001 |  | <0.001 |
|  |  |  |  |  |
| * Adjusted for child age, sex, and state | | | | |

*Notes: Variables significant at p<0.20 in bivariate analysis were entered into linear models.*

*^a^Level 3 multivariable model includes all statistically significant (p<0.15) distal variables as listed.*

*^b^Level 2 multivariable model includes level 3 model all statistically significant (p<0.15) intermediate variables as listed.*

*^c^Level 1 multivariable model includes level 3 model + 2 model + all statistically significant (p<0.15) proximal variables as listed*

## **Supplementary Table 9:** Difference-in-differences multivariable regression for children under 6 months from 2001 – 2016

| **Domain/Indicator** | **Outcome = HAZ** | | | |
| --- | --- | --- | --- | --- |
|  | **(Height for age z-score for under 6 months children)** | | | |
|  | **Period 2001 to 2016** | | | |
|  | **Bivariate regression coefficient** | | **Final multivariable regression coefficient*** | |
|  |  | b estimate (95% CI) |  | b estimate (95% CI) |
|  |  | *p*-value |  | *p*-value |
|  |  |  |  |  |
| **Distal level** | | | | |
| **Basic causes & Income poverty** | | | | |
| Wealth Index (six components using PCA) |  | 0.062 (0.039; 0.085) |  | 0.039 (0.011; 0.066) |
| (0 - 10)  (score 0-10, household) |  | <0.001 |  | 0.006 |
|  |  |  |  |  |
| Wealth Index # Year |  | 0.002 (-0.003; 0.006) |  |  |
|  |  | 0.417 |  | -- |
|  |  |  |  |  |
| Maternal education |  | 0.062 (0.041; 0.083) |  | 0.05 (0.027; 0.073) |
| (years of schooling) |  | <0.001 |  | <0.001 |
|  |  |  |  |  |
| Maternal education # Year |  | -0.002 (-0.005; 0.002) |  |  |
|  |  | 0.394 |  | -- |
|  |  |  |  |  |
| Paternal education |  | 0.049 (0.028; 0.071) |  |  |
| (years of schooling) |  | <0.001 |  | -- |
|  |  |  |  |  |
| Paternal education # Year |  | 0.002 (-0.002; 0.005) |  |  |
|  |  | 0.423 |  | -- |
|  |  |  |  |  |
| **% of residual variance explained by covariates** | | |  | **5.8%** |
| **Intermediate level** | | | | |
| **Inadequate feeding practices and food insecurity** | | | | |
| Duration of breast feeding |  | -0.011 (-0.059; 0.037) |  |  |
| (months, index child) |  | 0.649 |  | -- |
|  |  |  |  |  |
| Duration of breast feeding # Year |  | -0.0046 (-0.014; 0.0047) |  |  |
|  |  | 0.331 |  | -- |
|  |  |  |  |  |
| **Inadequate care and health services** | | | | |
| DPT vaccine [3 doses] |  | 0.187 (0.038; 0.337) |  | 0.226 (0.044; 0.408) |
| (%, index child) |  | 0.014 |  | 0.015 |
|  |  |  |  |  |
| DPT vaccine # Year |  | -0.019 (-0.048; 0.011) |  |  |
|  |  | 0.217 |  | -- |
|  |  |  |  |  |
| Skilled attendant at birth |  | 0.473 (0.271; 0.675) |  | 71.306 (3.601; 139.01) |
| (%, mothers) |  | <0.001 |  | 0.039 |
|  |  |  |  |  |
| Skilled attendant at birth # Year |  | -0.042 (-0.077; -0.008) |  | -0.035 (-0.069; -0.002) |
|  |  | 0.015 |  | 0.04 |
|  |  |  |  |  |
| 4+ antenatal care visits |  | 0.39 (0.206; 0.573) |  |  |
| (%, mothers) |  | <0.001 |  | -- |
|  |  |  |  |  |
| 4+ antenatal care visits # Year |  | -0.012 (-0.045; 0.02) |  |  |
|  |  | 0.452 |  | -- |
|  |  |  |  |  |
| Number of government hospitals |  | -2.232 (-4.615; 0.151) |  |  |
| (Total, per 10,000) |  | 0.066 |  | -- |
|  |  |  |  |  |
| Number of government hospitals # Year |  | 0.5094 (-0.0596; 1.0785) |  |  |
|  |  | 0.079 |  | -- |
|  |  |  |  |  |
| Number of primary health care centers |  | 1.365 (-1.723; 4.454) |  | -1268.033 (-2311.313; -224.754) |
| (Total, per 10,000) |  | 0.385 |  | 0.017 |
|  |  |  |  |  |
| Number of primary health care centers # Year |  | 0.7689 (0.2371; 1.3006) |  | 0.633 (0.113; 1.153) |
|  |  | 0.005 |  | 0.017 |
|  |  |  |  |  |
| Number of health posts or lower level health facilities |  | -0.081 (-0.168; 0.006) |  |  |
| (Total, per 10,000) |  | 0.067 |  | -- |
|  |  |  |  |  |
| Number of health posts or lower level health facilities # Year |  | 0.0281 (0.0113; 0.0449) |  |  |
|  |  | 0.001 |  | -- |
|  |  |  |  |  |
| Outreach clinics |  | -0.0026 (-0.0075; 0.0023) |  |  |
| (Total, per 10,000) |  | 0.297 |  | -- |
|  |  |  |  |  |
| Outreach clinics # Year |  | 0.00063 (-0.00037; 0.00163) |  |  |
|  |  | 0.214 |  | -- |
|  |  |  |  |  |
| Number of mother group meetings |  | -0.0001 (-0.0006; 0.0003) |  |  |
| (Total, per 10,000) |  | 0.577 |  | -- |
|  |  |  |  |  |
| Number of mother group meetings # Year |  | 0.00006 (-0.00013; 0.00025) |  |  |
|  |  | 0.543 |  | -- |
|  |  |  |  |  |
| **Unhealthy household environment** | | | | |
| Urban locality |  | 0.382 (0.162; 0.603) |  |  |
| (%, households) |  | 0.001 |  | -- |
|  |  |  |  |  |
| Urban locality # Year |  | -0.0283 (-0.0641; 0.0075) |  |  |
|  |  | 0.121 |  | -- |
|  |  |  |  |  |
| Open defecation |  | -0.427 (-0.607; -0.246) |  |  |
| (%, households ) |  | <0.001 |  | -- |
|  |  |  |  |  |
| Open defecation # Year |  | -0.0094 (-0.042; 0.0231) |  |  |
|  |  | 0.569 |  | -- |
|  |  |  |  |  |
| Piped water source |  | 0.123 (-0.034; 0.28) |  | -54.918 (-111.709; 1.874) |
| (%, households) |  | 0.124 |  | 0.058 |
|  |  |  |  |  |
| Piped water source # Year |  | 0.0408 (0.0126; 0.0689) |  | 0.027 (-0.001; 0.056) |
|  |  | 0.005 |  | 0.057 |
|  |  |  |  |  |
| Number of household members |  | 0.023 (-0.001; 0.047) |  | 0.023 (-0.001; 0.046) |
|  |  | 0.06 |  | 0.056 |
|  |  |  |  |  |
| Number of household members # Year |  | 0.0006 (-0.0043; 0.0054) |  |  |
|  |  | 0.823 |  | -- |
|  |  |  |  |  |
| **% of residual variance explained by covariates** | | |  | **8.6%** |
| **Proximal level** | | | | |
| **Disease** | | | | |
| ARI infection in last 2 weeks |  | -0.074 (-0.263; 0.116) |  |  |
| (%, index child) |  | 0.446 |  | -- |
|  |  |  |  |  |
| ARI infection # Year |  | -0.023 (-0.062; 0.015) |  |  |
|  |  | 0.233 |  | -- |
|  |  |  |  |  |
| Diarrhea infection in last 2 weeks |  | -0.0804 (-0.2806; 0.1197) |  | -80.422 (-155.91; -4.933) |
| (%, index child) |  | 0.43 |  | 0.037 |
|  |  |  |  |  |
| Diarrhea infection # Year |  | 0.035 (-0.005; 0.074) |  | 0.04 (0.002; 0.078) |
|  |  | 0.084 |  | 0.037 |
|  |  |  |  |  |
| **Maternal characteristics** | | | | |
| Maternal age |  | -0.011 (-0.025; 0.002) |  |  |
| (years, mothers) |  | 0.107 |  | -- |
|  |  |  |  |  |
| Age # Year |  | 0.00184 (-0.00096; 0.00464) |  |  |
|  |  | 0.196 |  | -- |
|  |  |  |  |  |
| Adolescent birth (<18 years of age) |  | -0.257 (-0.509; -0.005) |  | -0.318 (-0.564; -0.073) |
| (% mothers for index child within last 5 years) |  | 0.046 |  | 0.011 |
|  |  |  |  |  |
| Adolescent birth # Year |  | -0.052 (-0.097; -0.006) |  |  |
|  |  | 0.025 |  | -- |
|  |  |  |  |  |
| Older mother birth (≥35 years) |  | -0.175 (-0.51; 0.16) |  |  |
| (% mothers for index child within last 5 years) |  | 0.305 |  | -- |
|  |  |  |  |  |
| Older mother birth # Year |  | -0.067 (-0.154; 0.02) |  |  |
|  |  | 0.129 |  | -- |
| Body mass index |  | 0.063 (0.034; 0.092) |  | 0.038 (0.006; 0.07) |
| (kg/m2, mothers) |  | <0.001 |  | 0.02 |
|  |  |  |  |  |
| Body mass index # Year |  | 0.001 (-0.004; 0.005) |  |  |
|  |  | 0.777 |  | -- |
|  |  |  |  |  |
| Height |  | 0.051 (0.037; 0.066) |  | 0.049 (0.034; 0.063) |
| (cm, mothers) |  | <0.001 |  | <0.001 |
|  |  |  |  |  |
| Height # Year |  | 0 (-0.003; 0.002) |  |  |
|  |  | 0.773 |  | -- |
|  |  |  |  |  |
| Parity |  | -0.049 (-0.09; -0.008) |  |  |
| (Total children, mother) |  | 0.018 |  | -- |
|  |  |  |  |  |
| Parity # Year |  | 0.0002 (-0.0097; 0.0101) |  |  |
|  |  | 0.969 |  | -- |
|  |  |  |  |  |
| Interpregnancy interval |  | 0.001 (-0.002; 0.004) |  |  |
| (months, index mother) |  | 0.434 |  | -- |
|  |  |  |  |  |
| Interpregnancy interval # Year |  | -0.00035 (-0.00092; 0.00023) |  |  |
|  |  | 0.238 |  | -- |
|  |  |  |  |  |
| **% of residual variance explained by covariates** | | |  | **13.3%** |
| **Time** | | | | |
| **Time** | | | | |
| Year |  | 0.044 (0.037; 0.051) |  | -0.04 (-0.081; 0.001) |
|  |  | <0.001 |  | 0.058 |
|  |  |  |  |  |
| * Adjusted for child age, sex, and state |  |  |  |  |

*Notes: Variables significant at p<0.20 in bivariate analysis were entered into linear models.*

*^a^Level 3 multivariable model includes all statistically significant (p<0.15) distal variables as listed.*

*^b^Level 2 multivariable model includes level 3 model all statistically significant (p<0.15) intermediate variables as listed.*

*^c^Level 1 multivariable model includes level 3 model + 2 model + all statistically significant (p<0.15) proximal variables as listed*

# **Supplementary Appendix 6:** Programs and Policies

**Supplementary Table 10**: Detailed timeline of nutrition-specific and -sensitive laws, policies and programs in Nepal

| **ACTS/LAWS/REGULATIONS** | | |
| --- | --- | --- |
| 1. Nepal Food Act 1967 | Description | The Nepal Food Act, 1967 was created as the primary legislation overseeing food safety in Nepal. With a nationwide reach, the Act banned the production, sale or distribution of contaminated, unsafe or inferior foods. It also created the requirement for food establishments to have a license, regulates packaging and labeling requirements, established government bodies responsible for enforcing food safety rules and regulations, established the process for formulating food standards and quality and outlined prosecution for misbranding of food items as well as a detention process for foods under suspicion of contamination (94). Regulations resulting from this Act are funded by the Government of Nepal, and monitoring of these regulations is carried out by The Department of Food Technology and Quality Control under the Ministry of Agriculture Development. |
|  | Importance | Insufficient evidence on the impact on stunting, but a historical moment in relation to nutrition in the country |
|  | Theme(s) | Health |
|  |  | Nutrition |
| 1. Mothers Milk Substitute (Control of Sale and Distribution) Act & Regulation   (1992 – present) | Description | The Mothers Milk Substitute Act, 1992 was created with the aim of providing safe and adequate nutrition to infants by protecting and promoting breastfeeding and regulating mothers’ milk substitutes as well as the sale and distribution of foods for infants. It called for the creation of a Breastfeeding Protection and Promotion Committee to supervise compliance with the Act; review and approve labels submitted by infant food manufacturers and distributors to ensure compliance with the provisions of the Act; and formulating a national policy for the protection and promotion of breastfeeding. The Nepal Breast Milk Substitute Regulation, 1994 was created by the Government of Nepal to implement and achieve the objectives of the Mothers Milk Substitute (Control of Sale and Distribution) Act 1992. It provides detailed rules to be followed by inspectors appointed to monitor the provisions of the Act; by health workers accepting donations of equipment, products and accepting scholarships/grants; by manufacturers and distributors donating goods and equipment, marketing and/or labeling (95,96). |
|  | Importance | This was likely important to improvements in breastfeeding indicators and control on formula feeding, including ensuring the quality of formula. |
|  | Theme(s) | Health |
|  |  | Nutrition |
| 1. Iodized Salt (Production, Sale and Distribution) Act   (1998 – present) | Description | The Iodized Salt (Production, Sale and Distribution) Act was established to provide for the production, import, supply, sale and distribution of iodized salt in the appropriate quantity to prevent and eradicate health complications related to iodine deficiency. Along with the Act, awareness raising activities were initiated regarding the need for intake of iodized salt, including the establishment of committees to promote iodized salt, and home visits and school programs (97). According to the 2016 Nepal Demographic and Health Survey, iodized salt intake has reached 95% coverage in households across the country (98). |
|  | Importance | Likely not important for reducing child stunting |
|  | Theme(s) | Health |
|  |  | Nutrition |
| 1. Local Self Governance Act (1999-present) | Description | The Local Self Governance Act was legislated nationwide to decentralize and democratize governance within Nepal, including building up participation by all people and the institutional development of local bodies who would bear the responsibility for carrying out plans at the local level. The Act constituted the development of the self-governance system for local bodies to develop leadership and make decisions on matters affecting their institutions and the lives of their constituents. The main object of the Act is to support the decentralization process in general and the capacity building of the districts, in particular with respect to the rural infrastructure development, and improving rural accessibility. Monitoring and evaluation of this act is done by the Decentralization Implementation and Monitoring Committee at the central level, which consists of representatives from different ministries and the National Planning Commission. Funding is allocated to the local level by the Government of Nepal each year and local bodies have the authority to use funding to meet their needs (99). |
|  | Importance | Very important |
|  | Theme(s) | Health |

| **POLICIES/STRATEGIES/PLANS** | | |
| --- | --- | --- |
| 1. National Health Policy, 1991   (1991-2014) | Description | Created following the establishment of a constitutional monarchy and multi-party democracy in 1990, the National Health Policy 1991 aimed to improve the health standards of Nepal’s majority rural population by extending Basic Primary Health Services to the village level and making health facilities more accessible to rural people. The National Health Policy specifically targeted reductions in the infant mortality rate, under-five mortality rate, fertility rate and maternal mortality rate, as well as increasing the country’s overall life expectancy. Along with outlining a package of Basic Primary Health Services, key components of the National Health Policy included outlining a package of preventative health services, promotive health services, and curative health services, including at what level of the health system these services would be available, i.e. central, district or village. Following a review of the 1991 National Health Policy in 2012, a revised version of the policy was formulated in 2014 (100,101). |
|  | Importance | Very important |
|  | Theme(s) | Health |
|  |  | Nutrition |
| 1. Second Long Term Health Plan   (1997-2017) | Description | Following from the First Long Term Health Plan (1975-1990), which sought to improve the delivery of basic health services throughout the country, the Second Long Term Health Plan aimed to further address disparities in healthcare, promoting gender sensitivity and equitable community access to good quality healthcare services (102). The aims of the SLTHP included: providing a guiding framework to build successive periodic and annual health plans that improve the health status of the population; developing appropriate strategies, programs, and action plans that reflect national health priorities that are affordable and consistent with available resources; establishing co-ordination among public, private and NGO sectors and development partners. Key areas of focus of this plan included: Population control and family planning; Control and reduction of disease burden of communicable and non-communicable diseases; Health service delivery in an efficient way; Preventive and community health services; Curative and rehabilitative services; Ancillary Services and Information, Education and Communication; and Health Care Financing and Expenditure (103). |
|  | Importance | Very important |
|  | Theme(s) | Health |
|  |  | Nutrition |
| 1. Education for All   (2000-2015) | Description | Resulting from the 2000 World Education Forum on Education for All in Dakar, Senegal, Nepal’s own Education for All program aimed to better the education system in the country through a focus on ensuring access and equity in primary education; enhancing quality and relevance of primary education; and improving efficiency and institutional capacity. There were seven main areas of improvement for this program: early childhood care and education; universal primary education; youth and adult skills; adult literacy; gender equality; quality of education; and rights of indigenous people and minorities. Over the course of the program, a 77.7% gross enrollment rate was achieved and early childhood development centers were established. The intake rate of female children was also increased significantly and the overall literacy rate among marginalized communities was improved, as well as the adult literacy rate (104,105). |
|  | Importance | Very important |
|  | Theme(s) | Education |
|  |  | Poverty Reduction |
| 1. Poverty Reduction Strategy   (2002-2007) | Description | Resulting from the Government’s Tenth Five Year Plan (2002-2007), the Poverty Reduction Strategy focused on four key pillars: promoting faster and pro-poor economic growth; equitable access to social and economic infrastructure and resources for the poor and marginalized groups; social inclusion and targeted programs; and improved governance. The strategy was directed mainly at rural areas and focused on rural/agricultural growth. Examples of activities developed under the Poverty Reduction Strategy included: establishment of agriculture resource centers; promotion of cooperative and contract farming; integration of irrigation and micro-irrigation with agricultural intensification for commercializing agriculture; empowerment of women and disadvantaged groups through targeted programs; strengthening institutional capacity to meet World Trade Organization requirements; development of market centers (106). |
|  | Importance | Very important |
|  | Theme(s) | Poverty Reduction |
|  |  | Agriculture |
| 1. Nepal Health Sector Program (NHSP I)   2004-2009 | Description | The Nepal Health Sector Program I presented a nationwide roadmap for implementation of the health system, running from July 2004 - July 2009. The Program had seven key aims: increased access to and utilization of Essential Health Care Services; decentralized management of health facilities; public-private partnerships; sector management; sustainable financing; sector physical assets management and procurement of goods; human resources for health; and Health Management Information System improvements. Under the umbrella of this broad program, many other health and nutrition programs were nestled. Key components carried out within health facilities/health posts/communities included:  Nutrition: National Vitamin A Supplementation Program (semi-annual); availability and use of appropriately iodized salt through behaviour change communication and market measures; expansion of the semi-annual mass deworming program; promotion and expansion of the use of iron-folate in pregnant women; promotion of growth monitoring and counseling, Infant and Young Child Feeding, promotion of fortified complementary blended food;  Essential Health Care Services: Family planning, Safe Motherhood Program , perinatal care, child health care, Community Based Integrated Management Of Neonatal And Childhood Illness (CB-IMNCI), Expanded Programme of Immunization (EPI), Tuberculosis, Leprosy, HIV/AIDS/STD, Malaria/VBD, Outpatient services;  Control of infectious disease and zoonosis**:** Malaria prevention, vector borne disease prevention, lymphatic filariasis, soil-transmitted helminthes, and trachoma (107). |
|  | Importance | Likely important |
|  | Theme(s) | Health |
|  |  | Nutrition |
| 1. National Nutritional Policy and Strategy   (2004 – present) | Description | The National Nutrition Policy and Strategy aims to improve the nutritional wellbeing of all people in Nepal. It is the policy document that guides nutrition-related activities in the country and has led to the formulation of various nutrition plans and program such as the Multi-Sector Nutrition Plan (MSNP). The National Nutritional Policy and Strategy has 13 overarching objectives. These include reducing protein-energy malnutrition, anemia and infestation of intestinal worms among women and children; and reducing the prevalence of low birth weight and critical risk of malnutrition during exceptionally difficult circumstances. They also include commitments to virtually eliminate iodine deficiency disorders and vitamin A deficiency; improve household food security, promote the practice of good dietary habits, prevent and control infectious diseases, control the incidence of life-style related diseases; improve the health and nutritional status of school children; and strengthen the system for analyzing, monitoring and evaluating the nutrition situation (108). |
|  | Importance | Very Important as the first coordinated nutrition plan for the country. |
|  | Theme(s) | Health |
|  |  | Nutrition |
| 1. School Health and Nutrition Strategy   (2006 – Present) | Description | The School Health and Nutrition Strategy was developed with the goals of improving the use of School Health and Nutrition Services by school children; improving healthy school environments; improving health and nutrition behaviours and habits; and improving and strengthening community support systems and policy environments. Key activities under the umbrella of this policy included physical checkups, deworming programs and first aid services in schools, a school checklist to be adopted by schools to improve their environment including the health and nutrition behaviours of students; and Child Club mobilization to support school health and nutrition activities. Activities under this strategy reached 4 districts in 2009 led by Save the Children and 22 districts by 2012 through support from the Government of Nepal (109,110). |
|  | Importance | Likely not important as it does not target the right population/time period for under-5 stunting reduction. Influence on adolescent future mothers not yet seen. |
|  | Theme(s) | Education |
|  |  | Health |
|  |  | Nutrition |
| 1. Non-Formal Education Policy   (2007-present) | Description | The Non-Formal Education Policy was created to clarify the existing activities of the broad-reaching non-formal education programs taking place throughout Nepal with clearer guidelines. Non-formal education services target various groups, including illiterate people living in remote areas and the hinterlands, those living below the poverty line and wage laborers working in factories and farms. Key components of non-formal education programming include literacy, post-literacy and awareness raising programs; programs related to life long and continuous education, and skill development and income generation. Additional activities also include implementation of a literacy campaign program; environmental conservation/community development activities; women’s groups promoting saving and credit and women’s empowerment; coordination/cooperation/networking with NGOs; general saving and credit groups; library and resource centers; agriculture groups and horticulture groups (52). In 2015/2016, 136,489 adults participated in the continuing education aspect of non-formal education, 95.4% of which were women (111). |
|  | Importance | Likely important |
|  | Theme(s) | Education |
|  |  | Poverty Reduction |
| 1. School Sector Reform Plan   (2009-2015) | Description | The School Sector Reform Plan was a continuation of other education-focused programs taking place in Nepal such as Education for All (EFA), and was created with the aim of improving the quality and relevance of school education. The School Sector Reform Plan focused on restructuring of school education, improvement in the quality of education, and institutionalization of performance accountability. Core components of the plan were divided between improvements to basic education and secondary education (including increasing access), and institutional capacity-building (112). In 2016, an evaluation of the Nepal School Sector Reform Plan was conducted by the Ministry of Education and found that the literacy rate increased from 69% to 85% during the plan’s implementation (113). |
|  | Importance | Promising recent initiative, however likely not important to stunting decline between 1990 and 2010. |
|  | Theme(s) | Education |
| 1. Nepal Health Sector Programme – II   (NHSP –II)  (2010-2015) | Description | The Nepal Health Sector Programme II was the second phase of NHSP I. NHSP-I had been unable to advance the agenda of decentralized management of health facilities and deployment and retention of human resources as it had planned. As such, the Nepal Health Sector Program-II adopted a more participatory approach in its development. It aimed to push for achievement of the health sector Millennium Development Goals by 2015, increase access to essential health care services to the total population and develop sustainable financing for the health sector. It also aimed to bring equity in the utilization of quality health services by all people in Nepal. The main objectives of NHSP II were to increase access to and utilization of quality essential health care services; to reduce cultural and economic barriers to accessing health care services and harmful cultural practices in partnership with non-state actors; and to improve the health system to achieve universal coverage of essential health services. The Essential Health Care Services package for NHSP II included: reproductive health, child health, communicable disease control, non-communicable disease control, oral health, eye care, rehabilitation of the disabled, environmental health and curative care (114). |
|  | Importance | Likely important |
|  | Theme(s) | Health |
|  |  | Nutrition |
| 1. National Communication Strategy for Maternal, Newborn and Child Health   (2011-2016) | Description | Created to support the Nepal Health Sector Programme II (2010-2015), the National Communication Strategy for Maternal, Newborn and Child Health communicated information related to existing programs for maternal and child health, including the Safe Motherhood Program, the Community Based Integrated Management of Childhood Illness/Newborn Care Program (CB‐IMCI/NCP), Expanded Programme for Immunization (EPI) and other nutrition-related activities. Examples of the key components of this communication strategy included: promoting knowledge, high self‐efficacy, supportive norms, and improved maternal and newborn health behaviours in all social groups to increased institutional delivery; promoting knowledge of parents, families and communities about time, place and date of vaccination schedules, benefits and possible adverse events following immunization; promoting knowledge on childhood illness prevention and treatment of all social groups; and promoting improved breastfeeding knowledge among mothers to breastfeed children exclusively for the first six months. No evaluation of the impact of this communication strategy on relevant health indicators has been conducted (115). |
|  | Importance | Insufficient evidence |
|  | Theme(s) | Health |
|  |  | Nutrition |
| 1. Sanitation and Hygiene Master Plan   (2011-2017) | Description | The Sanitation and Hygiene Master Plan was a nationwide initiative created to overcome challenges and barriers regarding water, sanitation and hygiene in Nepal. In order to meet national and Millennium Development Goal targets, this Master Plan allowed for mainstreaming of the efforts of concerned stakeholders at various levels. All the concerned government agencies, local bodies, donors, I/NGOs, and other WASH stakeholders were encouraged to strictly adhere to the following guiding principles when planning and implementing hygiene and sanitation programs and water supply projects: Open Defecation Free as the bottom line; universal access to sanitation in water supply and sanitation projects; technology choices for household toilets; Village Development Committee or municipality is the minimum program area for program intervention; locally managed financial support mechanisms; sanitation facilities in institutions; and hand washing with soap and other behavior change approaches (116). During implementation of the Sanitation and Hygiene Master Plan, 27 districts were declared Open Defecation Free in 2015, rising to 37 districts in 2016 and 49 in 2018 (51,117,118)**.** |
|  | Importance | Promising recent initiative, however likely not important to stunting decline between 1990 and 2010. |
|  | Theme(s) | WASH |
|  |  | Health |
| 1. Scaling up Nutrition (SUN)   (2011 – present) | Description | Scaling Up Nutrition (SUN) is a global push for action and investment in order to improve maternal and child nutrition outcomes. Key strategic objectives include bringing people together, coherent policy and legal frameworks, aligning programs around a common results framework, financial tracking and resource mobilization. The main aim of SUN is engaging civil society organizations in advocating and sustaining political will for government action in scaling up nutrition. Nepal joined SUN in 2011, and using SUN guidelines, the Government of Nepal has created programs such as the Multi-Sector Nutrition Plan (MSNP), the Community Action for Nutrition Project (Golden 1000 Days Project) and the National Nutrition and Food Security Coordination Committee (119). |
|  | Importance | Promising recent initiative, however likely not important to stunting decline between 1990 and 2010. |
|  | Theme(s) | Nutrition |
|  |  | Health |
|  |  | Multi-sectoral Collaboration |
| 1. Multi-Sector Nutrition Plan (MSNP) - I   (2012-2017) | Description | The Multi-Sector Nutrition Plan (MSNP) I was developed as a result of the 2009 Nutrition Assessment and Gap Analysis and within the leadership of the National Planning Commission (NPC) and through support from key partners. MSNP I had the five-year goal of improving maternal and child nutrition. Specific outcomes that MSNP I aimed to achieve included a one-third reduction of Maternal Infant and Young Child (MIYC) undernutrition, measured in terms of maternal BMI and child stunting. MSNP covered 28 districts, prioritizing communities where there are high levels of deprivation and/or vulnerability to undernutrition Five key ministries, including Health and Population, Agriculture Development, Education, Urban Development, Federal Affairs and Local Development are involved in MSNP under the umbrella of NPC. MSNP has 3 main outcomes, each with corresponding activities:  Outcome 1: Policies, plans and multi-sector coordination improved at national and local levels;  Outcome 2: Practices that promote optimal use of nutrition 'specific' and nutrition 'sensitive' services improved, leading to an enhanced maternal and child nutritional status;  Outcome 3: Strengthened capacity of central and local governments on nutrition to provide basic services in an inclusive and equitable manner. Multi-sectoral plans were established at district level and district level coordination committees were set up to monitor the implementation of the program. Funding was provided by the government and various development partners. In 2018, MSNP II was launched, running until 2023 and covering 30 districts (120,121). |
|  | Importance | Promising recent initiative for multi-sectoral coordination to address broad population nutrition, however likely not important to stunting decline between 1990 and 2010. |
|  | Theme(s) | Nutrition |
|  |  | Health |
|  |  | Multi-sectoral collaboration |
| 1. Health Sector Strategy for Addressing Maternal Undernutrition   (2013-2017) | Description | The Health Sector Strategy for Addressing Maternal Undernutrition was a nationwide approach developed in line with the Nepal Health Sector Program II, the National Nutrition Policy and Strategy and the Multi-Sector Nutrition Plan, with the goal of improving the nutrition and health of adolescent girls, pregnant and lactating women. The main objective of the Health Sector Strategy for Addressing Maternal Undernutrition was to accelerate and sustain reductions in chronic maternal undernutrition and micronutrient deficiencies with a specific focus on disadvantaged and vulnerable groups. Key components of this strategy included: Institutional strengthening and government capacity building; Maternal nutrition integrated into health programs, including community-based approaches; Advocacy, community mobilization and behavior change communication for improved maternal nutrition by improving knowledge, diet and care practices; Focus on maternal nutrition beyond the health sector by involving appropriate non-health sectors in maternal nutrition efforts (multi-sectoral collaboration); Nutrition surveillance, monitoring, evaluation and research contributing to information on best practices for evidence-based planning, implementation and monitoring of effective maternal nutrition programming; National Communication Strategy for Maternal, Newborn and Child Health (122). |
|  | Importance | Promising recent initiative for multi-sectoral coordination for addressing maternal undernutrition, however likely not important on impact for stunting decline between 1990 and 2010. |
|  | Theme(s) | Nutrition |
|  |  | Health |
|  |  | Multi-sectoral Collaboration |
| 1. Food and Nutrition Security Plan   (2013-2022) | Description | The Food and Nutrition Security Plan was created in order to reduce hunger, malnutrition and poverty through improvements to sustainable agriculture, and is considered a significant government document for food security interventions for vulnerable populations. The Plan focuses on Agriculture, Fisheries, Food Quality and Safety, Forestry, Gender Equity and Social Inclusion (GESI), Horticulture, Nutrition Education and Training, Legislation, Animal Health and Production. This plan has supported the Multi-Sector Nutrition Plan (MSNP) aimed at improving maternal and child nutrition status, as well as the Agriculture Development Strategy goal of ensuring national food and nutrition security through a specific focus on the agriculture sector. The Government of Nepal’s Poverty Alleviation Fund is the key source of funding for this plan (123)*.* |
|  | Importance | Promising recent initiative for improving food security through agricultural productivity, however likely not important to stunting decline between 1990 and 2010. |
|  | Theme(s) | Nutrition |
|  |  | Food Security |
|  |  | Agriculture |
|  |  | Poverty Reduction |
|  |  | Multi-sectoral Collaboration |
| 1. Strategy for Infant, Young Child Feeding: Nepal   (2014-present) | Description | The Strategy Infant Young Child Feeding (IYCF): Nepal was developed to provide a robust framework for improving feeding and care practices within the country. It aims to improve the nutritional status, growth and development, health, and survival of infants and young children through optimal infant and young child feeding practices. The specific objectives of the strategy include: improving optimal breastfeeding practices; improving age appropriate optimal complementary feeding practices; ensuring enforcement of national legislation related to infant and young child feeding (such as marketing of breast milk substitutes and maternity protection); and improving the consumption of essential micronutrients (such as vitamin A among infants 6-24 months, and deworming tablets among infants 12-24 months). The strategy is geared towards attaining a set of behaviour impact targets by 2020, with monitoring and evaluation carries out by the Government of Nepal’s Child Health Division, though this has yet to be publicized (124)**.** |
|  | Importance | Promising recent initiative for improving dietary intake and feeding practices of young children, however likely not important to stunting decline between 1990 and 2010. |
|  | Theme(s) | Nutrition |
|  |  | Health |
|  |  | Multi-sectoral Collaboration |
| 1. Agriculture Development Strategy   (2014 –2034) | Description | The Agriculture Development Strategy (ADS) is a nationwide initiative created to grow the agriculture sector in Nepal so it can contribute to economic growth, improved livelihoods and food/nutrition security. The Agriculture Development Strategy has four strategic components for acceleration of agricultural sector growth related to governance, productivity, profitable commercialization and competitiveness (125). It also promotes inclusiveness (both social and geographic); sustainability (both natural resources and economic); development of the private sector and cooperative sector; and connectivity to market infrastructure (e.g. agricultural roads, collection centers, packing houses, market centers), information infrastructure and ICT, and power infrastructure (e.g. rural electrification, renewable and alternative energy sources). |
|  | Importance | Promising recent initiative for improving encouraging agricultural productivity, however likely not important to stunting decline between 1990 and 2010. |
|  | Theme(s) | Agriculture |
|  |  | Food Security |
|  |  | Poverty Reduction |

| **PROGRAMS/PROJECTS** | | |
| --- | --- | --- |
| 1. National Immunization Program   (1979-present) | Description | The National Immunization Program (NIP) is the priority program of the Child Health Division in Nepal and is believed to be one of the most successful public health interventions in the country, covering all 77 districts. Currently, eleven antigens are provided through routine immunization under the National Immunization Program, including BCG, DPT3/Hep B/HIb, Polio vaccine, Measles and Rubella, Japanese Encephalitis, Pneumococcal Conjugate Vaccine, and Tetanus-Diphtheria for pregnant women. The National Immunization Program receives a large bulk of its funding from the Government of Nepal, with the rest being provided by the WHO, UNICEF, the GAVI Alliance and the Bill and Melinda Gates Foundation (126). Annual Reports from the Department of Health Services under the Ministry of Health indicated that NIP covers more than 90% of the target population for most of the vaccines. NIP has been very successful despite the country’s political situation and a lack of human resources. However, there are still pockets of the country/population that remain hard to reach. Nepal attained polio free status on 27th March 2014; it has sustained maternal and neonatal tetanus elimination since 2005, and Japanese encephalitis is currently under control. Measles case-based surveillance is also being conducted to meet the target of elimination by 2019 (127). |
|  | Importance | Very important |
|  | Theme(s) | Health |
| 1. Joint Nutrition Support Program   (1989 – 1992) | Description | The Joint Nutrition Support Programme (JNSP) was launched in order for the government to implement major nutrition-related activities of the participating ministries with support from WHO/UNICEF. This was the first multi-sectoral approach for addressing malnutrition in Nepal, but it was not sustained. After three years of implementation, the program was terminated due to dissatisfaction from the funding agencies regarding the actual reach of the program. The main outcome of this program was the establishment of a nutrition section under the Department of Health Services in the Ministry of Health and Population (128). |
|  | Importance | Insufficient evidence that this had any meaningful impact on stunting decline but could be important as the first effort around multi-sectoral nutrition |
|  | Theme(s) | Nutrition |
|  |  | Multi-sectoral Collaboration |
| 1. Vitamin A Supplementation Program   (1993-present) | Description | Nepal’s National Vitamin A Supplementation Program is a nationwide program, covering all districts and reaching approximately 85% of its target population. The program has the following objectives: reduce child mortality and morbidity through prophylactic supplementation of children 6–60 months with high-dose (200,000 IU) Vitamin A capsules twice yearly in 32 priority districts; treat night blindness, severe malnutrition, prolonged diarrhea, and measles in all districts; and bring about behavior change to increase dietary intake of vitamin A and improve breast-feeding in 32 priority districts. Vitamin A supplementation is carried out at the local-level by Female Community Health Volunteers, with an annual cost US$1.7 million or around $1.25 to deliver two Vitamin A capsules to each participant (129,130). |
|  | Importance | Very important |
|  | Theme(s) | Health |
|  |  | Nutrition |
| 1. Community Based Integrated Management of Neonatal And Childhood Illness   (CB-IMNCI)  (1997-present) | Description | The Community-Based Integrated Management of Neonatal and Childhood Illness (CB-IMNCI) program is an integration of the Community Based Integrated Management of Childhood Illnesses (CB-IMCI) and the Community Based Newborn Care Programs (CB-NCP). It is a package of child survival interventions that address major childhood diseases (pneumonia, diarrhea, malaria, measles, and malnutrition) and major newborn health problems (birth asphyxia, bacterial infection, jaundice, hypothermia, and low birth weight), as well as counselling for breastfeeding. The main objectives of the program include: reducing neonatal morbidity and mortality by promoting essential newborn care services;  reducing neonatal morbidity and mortality by managing major causes of illness; and reducing morbidity and mortality by managing major causes of illness among under-5 years children The program delivers a package of newborn-specific, child-specific and cross-cutting interventions. Examples of interventions include promotion of a birth preparedness plan; identification and management of preterm and low birth weight babies; case management of children aged between 2‐59 months for 5 major childhood diseases; and improved knowledge related to immunization, nutrition and care of sick children (131). |
|  | Importance | Very important |
|  | Theme(s) | Health |
|  |  | Nutrition |
| 1. Basic and Primary Education Project   (1997-2010) | Description | The Basic and Primary Education Project was created by the Government of Nepal in response ongoing low levels of education attainment in the country and as a means to use education as a key to poverty alleviation. The Basic and Primary Education Project aimed to strengthen institutional capacities at national, district, and school levels for more efficient and better quality education services. The project was carried out in four phases, included:  Phase 1 (1999 – 2002) aimed to develop institutional capacity for qualitative and quantitative improvement in the five-year primary education system;  Phase 2 (2002 – 2005) aimed to consolidate institutional capacity building at national, district and school levels to raise primary school quality;  Phase 3 (2005 – 2008) aimed to continue to support institutional capacity building, development of the teaching service, and will expand access to eight years of education;  Phase 4 (2008 – 2010) aimed that by 2009, at least two thirds of school-age children will attain grade 8 in schools that are effectively supported by institutions at the community, district and national level (50). The literacy rate in Nepal improved significantly over the program period—48.6% in 2001 to 60% in 2011 (4). Due to this project, enrollment in primary education also increased, from 66.39% in 1999 to 98.26% in 2011, though dropout rates and repetition of grades remained stagnant (132). |
|  | Importance | Likely important as it was a school system capacity building initiative at the national and district level. |
|  | Theme(s) | Education |
| 1. Safe Motherhood Program   (1997 – present) | Description | The Safe Motherhood Program aims to save mothers’ lives and ensure a healthy perinatal period for both mothers and babies, shown through a reduction in the Maternal Mortality Ratio (MMR) and neo-natal mortality. Key components of the Safe Motherhood Program include: promoting inter-sectoral coordination and collaboration at all levels; strengthening and expanding delivery by skilled birth attendants and providing basic and comprehensive obstetric care services at all levels; supporting activities that raise the status of women in society; promoting research on safe motherhood, and strengthening community-based awareness on birth preparedness and complication readiness. As a result of the Safe Motherhood Program, the Policy on Skilled Birth Attendants (2006) and the National Blood Transfusion Policy (2006) were created. The Policy on Skilled Birth Attendants (2006) highlights the importance of skilled birth attendance (SBA) at all births and embodies the government’s commitment to train and deploy doctors, nurses and Auxiliary Nurse Midwives with the required skills across the country. The endorsement of the revised National Blood Transfusion Policy (2006) was another significant step for ensuring the availability of safe blood supplies for emergency cases. The Safe Motherhood Program also led to the creation of the Safe Delivery Incentive Program/Aama Program in 2005, which provides free delivery care and financial incentives to pregnant women to utilize antenatal and postnatal care services and to deliver in a health facility (61). |
|  | Importance | Very important |
|  | Theme(s) | Health |
|  |  | Multi-sectoral Collaboration |
| 1. Micro-Enterprise Development Programme (MEDEP)   (1998-2018) | Description | The Micro-Enterprise Development Programme (MEDEP) was created through collaboration between the Government of Nepal and UNDP. It aims to address poverty in Nepal through the development of micro-enterprises among low-income families for economic empowerment. It is a multi-partnership initiative between state institutions and the private sector. Key components of the program include: sensitization, awareness building and networking; identification and selection of potential entrepreneurs; and micro-enterprise creation and development training (133). Between August 2013 and July 2018, the government focused on the creation of 73,000 new micro-entrepreneurs while also providing scale up support for the 40,000 existing micro entrepreneurs. Targeted populations were extremely poor families, poor castes, poor indigenous groups, differently abled people and disadvantaged women (134). Funding for the four phases of this program has been supported by the Government of Nepal, UNDP and various foreign governments, including the United Kingdom, Australia, New Zealand and Canada (135). |
|  | Importance | Likely important for poverty reduction for more than half the country, but insufficient evidence on its impact to national level stunting decline. |
|  | Theme(s) | Poverty Reduction |
| 1. Nepal Nutrition Assessment and Gap Analysis   (2009) | Description | In 2009, the Nepal Nutrition Assessment and Gap Analysis (NAGA) was conducted and provided evidence-based recommendations for the health, agriculture, education and welfare sectors. Major recommendations of NAGA related to stunting reduction included:  1. Improving maternal and child nutritional care services through Mother, Infant and Young Child Feeding (MIYCF) and institutional strengthening;  2. Parental education, life skills and nutritional services for adolescent girls in order to prevent intergenerational stunting;  3. Control and prevention of diarrheal diseases and acute respiratory illnesses among young mothers, adolescent girls, infants, and young children through promotional campaigns and awareness; and  4. Ensuring availability and consumption of appropriate foods in terms of quantity, quality, frequency and safety. A comprehensive report was published which acted as key document for future planning of the nutrition sector. Based on these recommendations, in 2012 the Multi-Sectoral Nutrition Plan (MSNP) 2013-2017 was formed (128,136). |
|  | Importance | Very important as a catalyst for subsequent multi-sectoral action for nutrition |
|  | Theme(s) | Nutrition |
|  |  | Multi-sectoral Collaboration |
| 1. Integrated Nutrition Project (SUAAHARA)   (2011-2016) | Description | As a part of USAID’s strategy to help strengthen Nepal’s health and nutrition programs, and in consultation with the Ministry of Health and Population and relevant partners, the Integrated Nutrition Project (SUAAHARA) was launched. SUAAHARA aimed to improve and sustain the health and well-being of the Nepali people. The specific objective of this program was to improve the nutritional status of women and children less than two years of age through a comprehensive, community-based nutrition program that combines health, nutrition, agriculture and food security activities. This project was supported by the Government of Nepal’s Multi-Sector Nutrition Plan and the Hygiene and Sanitation Master Plan, reaching 41 districts total The main activities included in this program were:   1. Providing training on a package of integrated and evidence-based essential nutrition actions for health and non-health service providers, Female Community Health Volunteers, mothers’ groups, and household decision makers, especially mothers-in-law and husband; 2. Supporting improvements in nutrition and maternal and child health services through a focus on quality improvement. 3. Promoting clean water, sanitation and hygiene practices at the household and community level in support of Government of Nepal efforts to achieve “open defecation-free” status for key districts. 4. Enhancing health workers’ capacity to provide effective counseling on healthy timing and spacing of pregnancy as critical for good health and nutrition. Mainstream homestead food production activities (vegetables and backyard poultry farming) to increase year-round access to diverse and nutritious foods at home; 5. Enhancing multi-sectoral coordination on nutrition between the Government of Nepal and others actors for the effective planning, implementation and monitoring of integrated nutrition activities via technical assistance to national working groups (137). |
|  | Importance | Promising recent initiative, however likely not important to stunting decline between 1990 and 2010. |
|  | Theme(s) | Health |
|  |  | Nutrition |
|  |  | Agriculture |
|  |  | Food Security |
| 1. Mandatory Flour Fortification   (2011-present) | Description | Beginning in 2011 the Government of Nepal, with support from the Micronutrient Initiative, initiated Mandatory Fortification of Flour processed at urban roller mills. Mandatory Flour Fortification started as an approach to tackle iron deficiency anemia and night blindness, as well as neural tube defects in newborns after other programs were not effective in tackling these issues. This program helped in producing a wide variety of fortified foods made from flour such as bread, biscuits and noodles, among other products. Fortification included wheat flour fortification with iron (60 ppm, electrolytic elemental form), folic acid (1.5 ppm) and Vitamin A (1 ppm) per kilogram of flour at roller mills in selected districts (138). The population reached by this program is largely in urban areas which accounts for approximately 18% of the total population. However, the program has run into issues with scale up beyond urban roller mills, and as a result people in rural areas may have issues accessing these fortified products (139). |
|  | Importance | Insufficient evidence |
|  | Theme(s) | Health |
|  |  | Nutrition |
| 1. Community Action for Nutrition Project (Golden 1000 Days Project)   (2012-2017) | Description | The Community Action for Nutrition Project (Golden 1000 Days Project) was launched by the Government of Nepal and the World Bank. It aimed to adopt a life cycle approach in order to improve practices that contribute to reduced undernutrition in women of reproductive age and children under the age of two and to provide emergency nutrition and sanitation response to vulnerable populations in earthquake affected areas. Activities conducted by the Golden 1000 Days Project centered around the following:  1. Promoting and supporting good maternal nutrition during pregnancy and lactation;  2. Promoting and supporting optimal infant and young child feeding and care practices;  3. Giving special focus to the 1,000-day period within USAID health, nutrition, agriculture, and humanitarian assistance programs;  4. Social and behavior change  The project worked in 15 districts and targeted 25% of the most disadvantaged Village Development Committees (VDCs), or 292 VDCs out of total 1,148 (140). |
|  | Importance | Promising recent initiative for addressing community nutritional challenges, however likely not important to stunting decline between 1990 and 2010. |
|  | Theme(s) | Nutrition |
|  |  | Health |
|  |  | WASH |
|  |  | Agriculture |
| 1. Knowledge Based Integrated Sustainable Agriculture and Nutrition (KISAN)   (2013-2017) | Description | The KISAN project, part of USAID’s global Feed the Future (FTF) initiative, was a five-year program working to advance food security objectives by increasing agricultural productivity. Objectives include:   1. To improve the availability of and access to quality agricultural inputs including seeds, plant protection chemicals, organic fertilizers, irrigation systems, and credit for farmers; 2. To improve the capacity of agriculture extension workers, service providers, and farmers to deliver services more efficiently; 3. To increase the uptake of improved and sustainable agriculture production and post-harvest practices and technologies for targeted cereals and vegetables; 4. To facilitate market efficiency and farmer access to markets; and 5. To increase the organizational, entrepreneurial, and technical capacity of local organizations, including private sector actors, to better serve farming households.   The Project focused on 300 Village Development Committees in 20 districts and reached more than 500,000 Nepalese through 100,000 rural households with major commodities like rice, lentils, maize and vegetables. It also trained 103, 835 farmers in improved agricultural practices and technologies. The recently launched KISAN II project covers four additional districts that were affected by the 2015 earthquake (141). |
|  | Importance | Promising recent initiative for improving food security through agricultural productivity, however likely not important to stunting decline between 1990 and 2010. |
|  | Theme(s) | Food security |
|  |  | Agriculture |

# **Supplementary Appendix 7:** Qualitative Results

**Supplementary Table 11:** Qualitative Inquiry – Full Results

Results from the in-depth interviews and focus group discussions are organized according to type of stakeholder, by national and community-level perspectives. Results are summarized according to key themes including basic (contextual) factors, nutrition-specific and –sensitive policies and programs, intermediate and immediate causes. Supporting evidence and quotes were selected to demonstrate a range of participants’ diverse perspectives on the determinants, and policies and programs.

**1. National Stakeholder Perspectives**

In-depth interviews were conducted with 18 national key informants working in the health and nutrition sectors. Key informants’ responses were categorized into several major drivers based on our conceptual framework including basic or distal causes, nutrition-specific or sensitive programs, intermediate causes, and immediate or proximal causes.

## **Supplementary Table 12a:** Summary of National Stakeholders

| **Participant #** | **Affiliation** |
| --- | --- |
| Participant 1 | Representative from The World Bank (former) |
| Participant 2 | Representative from The World Bank |
| Participant 3 | Representative from the Ministry of Health and Population |
| Participant 4 | Representative from the Institute of Medicine |
| Participant 5 | Representative from Hellen Keller International/SUAAHARA |
| Participant 6 | Representative from UNICEF Nepal |
| Participant 7 | Representative from the Ministry of Health and Population (former) |
| Participant 8 | Representative from Nepal Agriculture Research Council |
| Participant 9 | Representative from the Institute of Medicine (former) |
| Participant 10 | Representative from the World Health Organization |
| Participant 11 | Representative from Civil Society Alliance for Nutrition, Nepal |
| Participant 12 | Representative from Patan Academy of Health Sciences |
| Participant 13 | Representative from the National Planning Commission (former) |
| Participant 14 | Representative from New ERA |
| Participant 15 | Representative from an INGO, Project HOPE |
| Participant 16 | Representative from Child Health Division, Department of Health, MoHP |
| Participant 17 | Representative from National Planning Commission (former) (Health Section). |
| Participant 18 | Representative from Government health sector |

***Contextual factors***

Key contextual factors that facilitated gains achieved in child growth, and reduced stunting include overcoming political instability and violence, improved education (overall, and among women in particular), improved women’s empowerment, increased use of remittances to improve quality of life and nutrition, urbanization, as well as reduction of poverty and increased accumulation of wealth.

*Political instability & conflict*

After a decade of political instability, conflict and civil war in Nepal, the transition to democracy and peace represents a potential enabler for improvements in chronic malnutrition. Between 1996 and 2006 internal conflicts between the Government of Nepal and the Communist Party of Nepal (Maoist) led to 13,000 deaths, 1,200 missing people and extensive internal displacement of population. The conflict had an impact on both population health and the health system, and literature suggests that several health outcomes including stunting improved over the conflict period (142). However, improvements since the end of the conflict were noted as this may have reduced access to health services and challenges relating to human resources for health. Further, key informants highlighted that despite conflict reductions, since the restoration of democracy and overcoming violence, reductions in stunting have been significant with substantial gains due to increased political will and commitment from the national government and policymakers towards improvements of nutrition. Further, improvements in access to health services may also have strengthened nutrition-related efforts.

*"In the Maoists conflict period access to health care was very limited because we had limited human resources and the service provision was disconnected, so the stunting reduction may have been slower." -* Representative from New ERA

*Education & empowerment*

Overall, net enrolment in primary school has increased from 66% in 1999 to 95% in 2017 (143). Disparities in education by gender have decreased, as the gender parity index of primary school comparing the enrolment of girls and boys has increased gradually between 1970 and 2017, from 0.18 to 1.06, respectively. In addition, female literacy (aged 15-24 years) has increased substantially from 32.7% in 1991 to 80.2% in 2011 (144). Increased education levels represent a key driver of stunting reduction in Nepal, especially increases in women’s educational status over time and improved awareness of feeding practices and children’s nutrition. These advances have substantially impacted social norms and practices, including women’s roles in their families and communities, as well as individuals’ earning potential. Non-formal education has also been a critical driver of increased skills and knowledge relating to health and nutrition, particularly childhood illness and ensuring adequate dietary intake.

*“Education has played an important role, if the people are not well-informed there will be no improvement in policy. For long-term improvement, education has played an important role.”* - Representative from Nepal Agriculture Research Council

*“The Non-Formal Education Policy - 2007 ha[d] great impact on society as it targeted people of every age group. It helped in creating awareness and creating [a] family environment as [a] forum for discussion of issues related to eating behaviour of children, childhood illness, meal plan, etc. Since earlier times non-formal education [has] been playing [a] great role.” –* Representative from INGO, Project HOPE

*“Educated women can form groups and organizations in the villages like cooperatives that have a lot of impact. There are different groups like groups for agriculture. If you go to Makawanpur side, you may find only carrots or single type of vegetables in the field of most households. These are examples of cooperatives investing in agriculture. Where cooperative movement is strong, status of women is also strong. Where women are in groups and they can speak up and look into others’ eyes. That’s empowerment. I consider this as important because cooperatives have led to platforms for them to discuss, learn and grow.”-* Representative from Patan Academy of Health Sciences

Improved female education and literacy rates have also contributed to greater women’s empowerment, role in families and involvement in decision-making processes in communities and families.

*“In the past 20 years, there have been vast differences. Even after a lot of trying women didn’t prefer to speak up, now they have become able to speak in public for their rights. So for the welfare of family and family nutrition, women*’*s empowerment should have played an important role”-* Representative from World Health Organization

*Remittances & purchasing power*

International migration has increased substantially, with the greatest increases of migrants observed between 2001 and 2011, when the proportion of population engaged in international migration increased from 3.2% to 7.3% (145). Migrants are primarily married males aged 15-44 years, with groups 15-29 and 30-44 representing 80% of international migrants (145). According to the 2011 national census, destinations of Nepalese migrants include India (37.6%), Middle East (37.6%), ASEAN (Association of Southeast Asian Nations) (13%), Europe (3.3%), other Asian countries (2.9%), USA and Canada (2.5%), other countries (2.1%) and unknown (1.1%) (145). The greatest concentration of remittance distribution is in the Central and Eastern regions, at 26.6% and 32.5% respectively send the greatest number of international migrant and limited remittance inflow in the Mountain region (except in Eastern Mountains) (145). Most national stakeholders indicated that remittances have contributed indirectly to stunting reduction in Nepal, by helping to reduce poverty, improve food security, as well as increase quality of living standards and food, and accumulation of wealth. Key informants indicated that income from remittances has risen rapidly in Nepal over the past few decades, which has had an impact on nutrition and other health-related areas, as well as overall country development. Increased purchasing power of individuals and families has helped to improve both the availability and quality of food.

*“Remittances have contributed, but indirectly. Remittance increases education, ensures food security and increases purchasing power. These things may contribute to malnutrition reduction.”-* Representative from the World Bank

*“Regarding purchasing power/remittance, it is one of the major factor[s] to be considered for stunting reduction. When remittance comes to [a] family, they use it for consumption of varieties of food, health care and on buying other luxurious things.”* - Representative from UNICEF Nepal

*Urbanization*

Nepal is primarily a rural country, with 81% of the population living in rural communities (Table 2). It is simultaneously the least urbanized country in South Asian, and the nation experiencing the greatest growth of urban populations, doubling from 3.6% in 1991 to 6.5% in 2001 (146). Internal migration is an important driver of Nepal’s urbanization, with rural to urban migrants representing 45% of the urban population, and this continues to increase over time. In addition, changes from rural to urban spaces represents a key contributor to the increased urbanization, as between 1991 to 2001 reclassification from rural to urban represented 50% of the total urban growth (146). Potential challenges from rapid urbanization may include inadequate delivery of public services to support growing urban populations including both access to piped water and improved toilet facilities (146). Key informants felt that urbanization has also contributed to stunting reduction as urbanization impacts food security, and facilitates access to health services, and improved employment and education opportunities. However, respondents also noted that urbanization has contributed to the creation of urban slums, where access to health services, employment opportunities and education continues to represent a challenge.

*"Urbanization increases employment opportunity, revenue is generated and social status increases."* - Representative from Nepal Agriculture Research Council

*Poverty Reduction*

Poverty has reduced substantially in Nepal between 1995 and 2010, as the poverty headcount ratio at $1.90 a day decreased drastically from 62% to 15%, respectively (144). The multidimensional poverty index (MPI) in Nepal has also shown improvements as it decreased from 0.313 in 2006 to 0.126 in 2014, and the proportion of population living in poverty decreased from 59% to 29%, over the same time period (147,148). Improvements in the economic status of the Nepalese people were observed over the past 20 years, and stakeholders felt that this has helped to drive the decline in stunting. Individuals highlighted that women’s employment has increased and remittances have also improved the economy and increased individuals’ purchasing power. Efforts to address poverty (e.g., poverty alleviation funds) have helped to support employment, by increasing individuals’ economic status, and helped to increase purchasing power. Inequity in stunting reduction was also reported, with individuals in rural areas experiencing less decline in chronic malnutrition.

*“Families in the low wealth quintile do have [a] high [rate of] cases of stunting. Whereas families in the high wealth quintile have lower cases of stunting. Rural and remote area babies are more stunted than urban.”-* Representative from National Planning Commission (Former)

***Nutrition-specific and –sensitive policies and programs***

Implementation of nutrition-specific and –sensitive policies, programs and interventions was recognized as a critical driver of stunting decline by national key informants. Many significant nutrition-specific policies and programs were created and/or revised during the last 20 years in Nepal. Policy events (e.g., laws, legislation, policies and interventions) discussed during key informant interviews were ranked and ordered according to the number times highlighted or endorsed by national stakeholder. Key policies or “upper tier” are described in greater detail below.

# **Supplementary Table 12b:** Hierarchy of Nutrition-Specific and –Sensitive Policy Events

| **Level of Influence** | **Policies Events** |
| --- | --- |
| Upper tier  (endorsed by 8+ key informants) | - Safe Motherhood Program (1997) - Poverty Alleviation Strategy (2002) - National Nutrition Policy and Strategy (2004) - Education for All Policy (2004-2009) - Sanitation and Hygiene Master Plan (2011-2017) (including previous efforts in sanitation and hygiene) - Multi-Sector Nutrition Plan, 2013-2017 (triggered by Nepal Nutrition Assessment and Gap Analysis 2009) |
| Middle tier  (endorsed by  6-8 key informants) | - Expanded Program on Immunization (EPI) (1979) - National Health Policy (1991) - Vitamin A Supplementation (1993) - Breast Milk Substitute Act (1992) and Regulation (1994) - Basic and Primary Education Project (1997-2010) - Iodized Salt Act (1998) - Nepal Health Sector Program (2004-2009) - School Health and Nutrition Strategy (2006) - Nepal Health Sector Program (2010-2015) - Agriculture Development Strategy (2014) (along with Nepal Agriculture Perspective Plan) - Golden 1000 Days (2014) |
| Lower tier  (endorsed by  0-5 key informants) | - Nepal Food Act (1967) - Nepal Food Regulation (1970) - Joint Nutrition Support Program (1976) - Second Long-Term Health Plan (1997-2017) - National Nutrition Guidelines (1998) - National Plan of Action on Nutrition (1998) - Ninth Five Year Plan (1998-2002) - Local Self-Governance Act (1999) - Five Year Plan of Action for Control of Anemia among Women and Children in Nepal (2006-2011) - Non-Formal Education Policy (2007) - School Sector Reform Plan (2009-2015) - Integrated Nutrition Project (SUAAHARA) (2011-2016) - Mandatory Flour Fortification (2011) - Maternal, Newborn and Child Health Communication Strategy (2011-2016) - Health Sector Strategy for Addressing Maternal Undernutrition (2013-2017) - Knowledge-based Integrated Sustainable Agriculture in Nepal (KISAN) (2013-2018) - Infant and Young Child Feeding (2014) - Multi-Sector Nutrition and Food Security Advocacy and Communication Strategy (2015-2020) |

The *National Nutrition Policy and Strategy* represents a critical guiding document for the nutrition sector since 2004. It aims to improve the nutritional wellbeing of the population of Nepal and to support socioeconomic development. Key components include a focus on protein-energy malnutrition, anaemia, iodine deficiency disorders, worm infestation, low birth weight, malnutrition, lifestyle-related diseases, and to strengthen the health system. In addition, the use of fortification- and community/facility-based platforms to deliver vitamin A supplementation program and salt iodization were identified as key contributions to improved nutrition and stunting reduction (108).

*“The Joint Nutrition [Support] Program in 1986, National Nutrition Policy and Guideline[s], National Nutrition Policy, School Health Nutrition Strategy, Poverty Reduction Strategy, Nepal’s Three- Year Plans after the 2000s, and the Nepal Health Sector Programs are key policies and strategies that may have played a dynamic role in stunting reduction.” -* Representative from UNICEF Nepal

More recently, the *Multi-Sector Nutrition Plan (MSNP) 2013-2017* was implemented, and developed based on recommendations from the *2009 Nepal Nutrition Assessment and Gap Analysis (NAGA)*. Conceptualizing nutrition and stunting as a multi-sectoral problem was identified as critical driver of stunting reduction.

*“I think the main strength for success was the multi-sectoral approach. MSNP is designed with a multi-sectoral approach for the prevention of malnutrition. As we know that different factors are involved, so we should take care of it like Agriculture; we should use new technology and new methods there.”* - Representative from the World Bank

*“NAGA [Nepal Nutrition Assessment and Gap Analysis] is actually a break through document. It has contributed in bringing about the Multi-Sector Nutrition Plan in the country. The major NAGA policies have recommended that nutrition is not only the business of the MoH, it alone can’t improve nutritional status. So, instead we should move with a multi-sector approach.”* - Representative from Ministry of Health and Population

The Safe Motherhood Program was identified as a potential initiative that influenced stunting reduction through improvements to reproductive/maternal/newborn health outcomes, access to health services, as well as largely due to decreases in total fertility rate. Since its introduction in 1997, this program has aimed to improve maternal and newborn health, through promotion of intersectoral coordination, strengthening and facilitating access to skilled attendance at birth, and comprehensive obstetrical care.

*“I think TFR reduction might have definitely contributed to stunting reduction. It is a major reproductive health issue under [the] Safe Motherhood Program while analyzing DHS data. I can see it has reduced over the years but I cannot say how much it has contributed to stunting reduction but then improvement in fertility rate might have definitely contributed in stunting reduction (directly or indirectly) in various ways.” -* Representative from New Era

*“The reproductive health needs of women, including safe motherhood, are central to human development and must be central to the provision of health services. The promotion of motherhood health encompasses safe delivery of healthy and wanted babied, birth spacing, voluntary informed choice, prenatal and post-natal care. Fertility regulation is a preventive health measure and is also a way of achieving gender equality. Though there is no evidence depicting the correlation between stunting and fertility. But I do feel they are correlated (smiling….). Because the major interventions of nutrition are: birth spacing. As there is a modern trend of having smart couple who thinks about the small family size and proper space in between the children. If women gives birth to a child frequently, then along with women the child gets affected severely with low birth weight, poor physical and cognitive development and yes this result to stunting. There is a vicious cycle of malnutrition and poor health of mothers.” -* Representative from Ministry of Health and Population

Key policies/interventions in nutrition-sensitive sectors, such as *Poverty Alleviation Strategy,* as *WASH* policies and programs (e.g. *Sanitation and Hygiene Master Plan*), and the *Education for All* policy, were also mentioned by national key informants as contributing to stunting decline through providing a conducive environments for nutritional improvement.

*“Policies like [the] Poverty Alleviation Strategy have helped to reduce the poverty level of our country to a great extent. When people are poor, they can’t have enough food to eat, place to reside and they are deprived of health care also. This strategy helped the people by advocating in central level for creating employment opportunities, enhancing the income generation of the people by giving them skill-related training for earning money. Earning money increased their purchasing power which means good feeding practices and proper health facilities also. So it has impact on decreasing the stunted children.” –* Representative from Civil Society Nepal

*“Poverty alleviation fund has supported in livelihood generation, which helped in increasing purchasing power and ultimately should have contributed in reduction of malnutrition.”-* Representative from World Bank

Water, sanitation and hygiene (WASH) policies and programs were identified as critical to ensuring adequate access to improved and safe sources of water and achieving reductions in open defecation rates in rural communities. These efforts have decreased infections and diseases during childhood, and improved nutritional status among children.

*“WASH-related policies were also quite useful in reducing infection. Campaigns on ODF (Open Defecation Free) were instrumental in reducing infections among children, thereby improving nutritional status including stunting.”* - Representative from the Ministry of Health and Population (former)

*“Sufficient and safe water is needed for growth and development. This plan [Hygiene and Sanitation Master Plan] was focused on infection prevention and disease prevention like diarrhea, dysentery, typhoid and jaundice. Undernourished children are at high risk of falling in a vicious cycle of infection and undernutrition, one leading to another.”-* Representative from National Planning Commission (Former)

Education policies that emphasized increased formal and non-formal educational opportunities, offered through the national *Education for All* policy, were highlighted by several national stakeholders as possible interventions that have improved nutrition. The *Education for All Policy* was implemented from 2000-2015 and aimed to improve the education system in Nepal. Its primary components included: i) early childhood care and education; ii) universal primary education (particularly among girls); iii) youth and adult skills; iv) adult literacy; v) gender equality; vi) quality of education; and vii) rights of indigenous people and minorities (149). The *Non-Formal Education Policy* aimed to promote literacy, training and vocational skills for increased livelihood with groups in remote areas, out-of-school youths, and adults (150).

*“Definitely “Education for all” may have helped to improve nutrition status. In education main priority was given for female education and also for deprived and marginalized population. Many females are now educated in comparison to past. This strategy helped to educate the Nepalese people and also raised the awareness level.”* - Representative from Hellen Keller International/SUAAHARA

*“I have also helped to some extent in Education for all program and non-formal education program. Education is the process of empowerment. If she can read, speak up, and can access various things. It has multiple effects. Education is very important. I am not just talking about formal education, it also includes non-formal education. Educated women can form groups and organization in the villages like cooperatives that have a lot of impact. Yes “Education for all” program may have helped for the stunting reduction” -* Representative from Academia, Patan Academy of Health Sciences

*“The Non-Formal Education Policy-2007 ha[d] great impact on society as it targeted people of every age group. It helped in creating awareness and creating [a] family environment as [a] forum for discussion of issues related to eating behavior of children, childhood illness, meal plan, etc. Since earlier times non-formal education [has] been playing [a] great role.” –* Representative from INGO, Project HOPE

Other ‘middle-’ and ‘lower-tier’ identified by key informants that may have contributed to declines in stunting included the Infant and Young Child Feeding (IYCF) initiatives implemented due to the Mothers Milk Substitute Act, the Golden 1000 Days Project, the Community-Based Integrated Management of Neonatal and Childhood Illness, the School Health and Nutrition Program, iron and folic acid supplementation, Vitamin A Supplementation Program and projects. In particular, efforts to improve feeding practices through encouraging exclusive breastfeeding and limiting the promotion and use of breast milk alternatives.

*“Okay, the main role of these policies in declining the stunting rate were: IYCF via [the] BMS act, which is one of the proven preventive interventions. It has the single greatest potential impact on child survival. The Government of Nepal and Child Health Division has highly prioritized the IYCF program and its strategies.” -* Representative from Ministry of Health and Population

*Stakeholder Involvement and Coordination*

The contributions and coordination of diverse stakeholders, including development partners and civil society, have represented substantial drivers of improved nutrition and reduction of stunting in Nepal. The establishment of the *Multi-Sector Nutrition Program* was the result of ongoing and long-term commitments of individual sectors including health, education and agriculture to address malnutrition. Further, increased coordination of key stakeholders in the MSNP through the National Planning Committee has helped to align government, non-governmental organizations and donors in nutrition-related efforts across multiple sectors. Further, focusing on current needs and priorities in the population has helped to provide strategic direction for donor funding and prioritization of issues.

*“Stunting reduction has been [the] output of the great efforts from governmental and as well as efforts from [the] non-governmental side. The data of today’s stunting reflects the combined effect of both sectors.” -* Representative from Civil Society Alliance for Nutrition, Nepal

*“There is much more improvement in co-ordination between [the] past and present situation. First, we were working in isolated situation[s]. But after MSNP started, [a] co-ordination mechanism was formed. Every nutrition program goes through this mechanism in order to avoid duplication and to conduct it in [an] informed way. Due to MSNP there have been great improvement[s] in co-ordination. Still we have to do a lot in co-ordination part. Overall, we should co-ordinate all the high level related ministries to create a specific objective in nutrition i.e. [a] multisectoral approach. But it has greatly improved than [the] past, there is frequently gathering of developmental partners, sharing of work is done there. There is establishment of [a] forum in [the] National Planning Commission. It is improved now.”* - Representative from Civil Society Alliance for Nutrition, Nepal

*Challenges to implementation of policies/programs*

Key informants highlighted several potential challenges relating to the implementation of policies and programs that may continue to limit efforts and progress in stunting reduction. These included:

- Access to health care due to a lack of infrastructure (e.g., roads and transport) continue to limit access to health facilities;
- Limited skilled human resources in many rural and hard-to-reach communities ;
- Inadequate access to improved toilet facilities in both urban and rural areas and continued concerns regarding the safety of piped water sources and communicable diseases;
- Further efforts and coverage of behaviour change and communication regarding nutrition and dietary intake are needed particularly in hard-to-reach communities;
- The extensive role of donors, a lack of ownership and capacity in the government, and challenges relating to the sustainability of nutrition policies and programs were also identified as barriers that may limit progress in stunting reductions; and
- Limited national coverage of cash transfer programs, and concerns regarding its sustainability were challenges identified by national stakeholders. Cash provided may not be spent to improve health or nutrition, and efforts to change behaviours and encourage the investment of funds on nutritious foods are needed.

***Underlying Causes***

Improved sources of water and an increase in adequate sanitation facilities have led to reductions in infections, and helped to disrupt the cycle of disease and malnutrition among children. Increased accessibility to universal health services in communities and an emphasis on local health promotion and prevention of diseases have increased the availability and utilization of services. In addition, improvements in food security and agricultural practices have helped to improve diet diversity and the availability of quality foods.

*Improved household environment*

Substantial efforts and gains to improve sanitation and toilet facilities^[[1]](#footnote-1)^ are evident, as the proportion of population that engages in open defecation has decreased rom 75.3% of the population in 2001 to 20.7% in 2016. In addition, access to improved water sources^[[2]](#footnote-2)^ have led to greater access and availability of piped water, increasing from 33.5% in 2001 to 47.1% in 2016. National key informants felt that overall the household environment has improved substantially. Widespread efforts for community sensitization and introducing low-cost toilets were highlighted a key contributions to these achievements. National key informants felt that improved sanitation and safe drinking water was associated with decreased childhood infections and diseases, as well as overall nutritional status and child health.

*“WASH is an effective and easy way to control infestation, worms, microbes and pathogens. It helps in the control of diarrhea and other various infectious diseases. This is also a very effective plan for reducing child stunting.”* - Representative from Nepal Agriculture Research Council

*Increased access to health services*

The adoption of the 1991 *National Health Policy* (100) aimed to increase access to health services particularly for rural, vulnerable and marginalized populations. This was the first stage of Nepal’s comprehensive framework and subsequent introduction of long-term health strategic plans. As part of the *Long-Term Health Plan*, the Government of Nepal introduced the Essential Health Care Services Package in 1999 (151) and this has helped to catalyze efforts towards achieving universal health coverage (152). Further, despite long periods of conflict, Nepal continued to provide health services despite instability, and targeted/reached particularly vulnerable and marginalized populations through the use of community-based approaches, including the use of Female Community Health Volunteers and women’s groups (153).

National stakeholders felt that these commitments increased the accessibility and utilization of health services, particularly for maternal, newborn and child health. Provision of free maternal and child health services and free essential drugs at all community health centres, health posts and primary health care centres have been instrumental to increased access and utilization of health services. Community health workers and female community health volunteers have been recognized as an integral part of the community-based health programs in Nepal, and their critical role in health promotion and prevention of disease in communities was recognized by national key informants. In addition, introduction of the Community-Based Integrated Management of Childhood Illnesses (CB-IMCI) has increased the availability and accessibility of primary care (e.g., malnutrition, measles, malaria, etc.) using health facilities and community outreach clinics. Several respondents highlighted that improved access of health services represents an important factor that has helped to break the infection-malnutrition cycle, through the prevention and management of diseases among children at-risk for and experiencing chronic malnutrition.

*“…Access to healthcare was limited in [the] past and it has been improved now. Institutional delivery has increased over the years, there have been a lot of improvements in maternal and child health (newborn care, IMCI etc.) and the prevalence of communicable disease has also reduced and immunization coverage is also increased.” -* Representative from New ERA

*“Outreach clinics, health promotion activities, etc. through the health system have also helped in changes. Health promotion related activities through FCHVs, extension of health services through Community Health Units are major changes….”-* Representative from World Health Organization

However, several respondents identified inequities in access to health services as potential factors that may limit improvements in stunting. Low maternal education, living in rural and hard-to-reach areas and the lowest wealth quintile represent barriers to accessing health services. Challenges relating to infrastructure including roads and transport may also limit access to health facilities, particularly in rural communities. Key informants felt that these inequities have contributed to child health indicators, including nutritional status for children.

*Improved Food Security*

Improved food security has contributed both directly and indirectly to improvements in overall nutritional status, including stunting. Since 1995/96, an increase in agricultural production of staples (e.g., paddy/rice, wheat and potato) is observed, with potato emerging more recently as an important crop in the mountain areas. Geographic inequities in arable land are evident in Nepal with agricultural households operating land primarily in the Hills (58%), Terai (43%) and limited participation in agriculture in the Mountains (9%) (59). Key trends that may threaten food security in Nepal include an increasing food balance deficit, feminization of agriculture and the increased importance of non-agriculture income generation activities (e.g., remittances), and changes in land ownership and use of land (59). Key informants felt that advances in agricultural practices (e.g., mechanization of agriculture and hybridization of food crops), have improved food production, helped to diversify diets, and increased the population’s consumption of nutrient-rich food. Indirectly, the use of more mechanized farming and agricultural practices have helped to decrease women’s workloads and enabled them to provide greater time and care for families, and children.

*“Agriculture is very nutrition-sensitive. It contributes to malnutrition both directly and indirectly. Mechanized agriculture has reduced workload in women, increased productivity, and increased time for care of children. This mechanization has increased over time. For example, irrigation…..[the] Agriculture Development Strategy has also focused on mechanization. Beside this, [the] focus is now being given [to] consumption along with sell[ing] after increased production. Directly, it is related to food security, which ultimately influence nutrition status.”* – Representative from the World Bank

***Immediate Causes***

Key proximal factors that have supported stunting reduction include a decrease in infectious/communicable diseases, improved dietary intake and decreased fertility.

*Reduced burden of child diseases*

The *Community-Based Integrated Management of Childhood Illness* (CB-IMCI) aims to improve the capacity of health providers, strengthen the health system, and improve family and community practices (154). This initiative is an integrated package of interventions that supports the diagnosis, treatment and management of childhood disease (e.g., pneumonia, diarrhea, malaria, measles and malnutrition), newborn health challenges, and for health promotion (e.g., exclusive breastfeeding). Introduced in 1997, key components of the IMCI program include distribution of iron, zinc, oral rehydration solution (ORS), Vitamin A, immunization, child nutrition, breastfeeding promotion and referral in case of danger signs, by female community health volunteers (23,154). Implementation of the *National Immunization Program* (NIP) represents a long-standing public health priority in Nepal. Since its introduction in 1979, the program has expanded to cover all districts, and has reached 90% of the population to provide 11 antigens through routine, free childhood immunizations at health facilities, community health centres, private clinics and mobile and outreach efforts (127). National stakeholders indicated that the burden of child diseases in Nepal has declined over time, including both mortality and morbidity. In particular, reductions of intestinal/parasitic worms and vaccine-preventable diseases were observed. These improvements were seen as a result of improved access to health facilities and public health efforts, including coverage of immunizations, deworming initiatives and iron supplementation of women. Further, key informants indicated that poverty reduction and efforts to improve WASH and local access to key interventions (e.g., immunization) have also likely contributed to the declines in infections.

*“Factors within health sectors like service coverage improvement, like immunization of children, deworming, iron supplementation among mothers, etc. All have contributed to some extent for reduction in morbidity and mortality in children.”-* Representative from Patan Academy of Health Sciences

*Improved dietary intake*

Most national stakeholders reported significant improvements in the dietary intake of both women/mothers and children over time, in terms of both macro- and micronutrients. Increased access to information and health promotion by FCHVs and mass media (e.g., radio/TV) have contributed to lifestyle and health behaviour changes. The promotion of early initiation and exclusive breastfeeding for six months, and continued breastfeeding until at least 2 years of age, were highlighted as key factors that have improved dietary intake among children in Nepal. In addition, improvements in local food production and increased availability of sources of protein (e.g., fish) were observed. In addition, national key informants highlighted that improvement in local food production, complementary feeding of enriched (“super flour”) porridge, increased intake of green leafy vegetables, and increased intake of fish and fish production, as factors that have supported improved dietary intake among women and children.

*“…Another one is infant and child feeding practices, food recommendations on dietary diversity, exclusive breastfeeding until six months.”* - Representative from the Institute of Medicine (former)

*“Access to information has increased, which may have contributed to changes in lifestyle, dietary practices and the living standard of people. While these changes may also have some negative impacts, it may have played a role in stunting reduction. Use of mass media has increased but its impact in the health sector still needs to be measured. Direct health-related information through FCHVs and health workers for behaviour change have arguably played a greater role. Radio and TV programs have help to sensitize people on health matters.” -* Representative from New ERA

*“Good nutrition and breastfeeding promotes and strengthens optimum growth and development of the babies. Infant feeding is not only providing nutrition to an infant but also is a psychological, social and educational interaction between parent and infant. Breast milk provides all the essential nutrients fostering growth and development of children which is impossible to mimic or replace with any other kind of food. It is priceless food for children under-5 years*”. - Representative from Ministry of Health and Population”

Proper infant and young child feeding (ICYF) practices can substantially improve child health outcomes, including nutrition and stunting. These efforts include early initiation of breastfeeding, exclusive breastfeeding until six months, and the introduction of appropriate complementary foods and breastfeeding (until 2 years). In Nepal, breastfeeding indicators have demonstrated substantial improvements over time. Early initiation of breastfeeding within one hour of birth increased from 17.8% in 1995 to 54.9% in 2016 and prelacteal feeding practices decreased from 40.9% in 2001 to 28.6% in 2016, highlighting potential improvements in breastfeeding promotion and addressing cultural taboos and concerns regarding colostrum. However, exclusive breastfeeding for infants aged 0-5 months decreased from 74.8% in 1996 to 66.1% in 2016 (70). Most national respondents felt that improved ICYF was a critical contributor to improved child nutrition, and increased campaigns and awareness on exclusive breastfeeding supported increased rates.

*“It is [a] misconception that [a] child should only be fed with liquid foods. They need energy dense food and more foods [or] else they get malnourished by the age of two years. This type of information is well spread in rural villages as well and has positively changed their feeding practices”* - Representative from the Institute of Medicine (former)

*“Good nutrition and breastfeeding promotes and strengthens optimum growth and development of the babies. Infant feeding is not only providing nutrition to an infant but also is a psychological, social and educational interaction between parent and infant. Breast milk provides all the essential nutrients fostering growth and development of children which is impossible to mimic or replace with any other kind of food. It is priceless food for children under-5 years*”. - Representative from Ministry of Health and Population”

*Maternal Characteristics*

Drastic declines in adolescent fertility and increased age of marriage have been observed. Adolescent fertility rates have nearly halved from 113.9 per 1,000 girls aged 15-19 years in 2000 to 62.1 in 2016 and the age at first marriage has increased from 17.0 years in 2001 to 18.1 years in 2016 (Table 2). Maternal characteristics, including decreased fertility, increased maternal age and increased spacing between pregnancies, were identified by national key informants as influencing under-five stunting decline. These factors were linked to increased contraceptive use, improved food security, addressing issues of maternal depletion, as well as indirectly through increased care and food security in families.

*“The fewer number of children you have, the more attention you can give. The more the gaping is increased; the better it is. Decrease in TFR [Total Fertility Rate] increases care practices and food security in the family.”* - Representative from New ERA

*“The total fertility rate has been decreased over the years and it contributed indirectly in increased food security and increased care. If there are less children in a family the care practice increases and the food security also increases. Now the people like to have a smart family with one or two children only. The use of family planning methods has also increased in comparison to the past this may have contributed in decreased TFR [Total Fertility Rate]”* - Representative from Ministry of Health and Population

*“It is because changes are seen in different things like fertility, increase in age of marriage and another thing is that what will happen if mother[s] give frequent birth to children? It is known as maternal depletion where total nutrition including total iron, micronutrients lowers. In that state, if she gives birth to another child, the child won’t be healthy. This cycle of change that is increase[s] in age of marriage, decrease in number of times of pregnancy, increase in women literacy has contributed a lot.” -* Representative from Patan Academy of Health Science

*Child characteristics*

Low birth weight was outlined by national key informants as a possible factor contributing to stunting, and decreases in low birth weight were associated with decreased adolescent fertility.

*“Children whose birth size is small genetically will always be stunted but if birth size is small due to malnutrition in children or their mother, then that can be recovered through adequate food later on.”* *-* Representative from WHO

**2. Mothers in Communities Perspectives**

A comparison of older and younger mothers was conducted to understand the household-level nutrition transition in Nepal. Mothers with under-5 children born in the 1995-2000 period were selected as this was a period characterized by political instability, armed conflict and largely vertical approaches and delivery of health initiated. It also predated significant coordinated and cross-sectoral approaches to address underlying determinants of chronic undernutrition. Mothers with children born in recent 5 years (2010-2015), contrarily, had the opportunity to benefit from several, focused and streamlined nutrition-relevant initiatives in Nepal, particularly those implemented in communities. Key basic, underlying and immediate drivers of stunting reduction were analyzed and compared by community and timing of the birth of children. During FGDs in both community settings, mothers recognized multiple driving factors and determinants of improvements to the health and nutritional status of their children, affirming the need for a multifaceted, multilevel approach to target nutrition in Nepal.

***Contextual factors***

Increased socioeconomic status, poverty reduction and increased education and women’s empowerment were identified by mothers in communities as key enablers to gains in child growth and nutrition.

*Socioeconomic status*

Improved socioeconomic status and poverty reduction over the last 20 years were described by all mothers in communities as key enabling factors. This improvement was attributed to increased availability and use of remittances, as well as growth of household agricultural production.

*“…compared to early days, it has improved a lot. We had nothing. We were very much poor. The major source of income was only agriculture. We struggled for minimal day-to-day survival. But now the times have changed. We are now economically strong. Our sons/daughters are abroad. They send us money. We do have our own agricultural land. We consume food for ourselves from there and also sell seasonally.” (Smiling*) - P1: Mothers of children born in 1995-2000, Dukuchhap

*Education & Empowerment*

Transformation of gender norms and roles was recognized as possible facilitators to stunting reduction. Women with children born in the 2000s and between 2010-2015 felt that female education has increased substantially and that this has helped to expand traditional domestic and family contributions. This contributed to increased independence, employment opportunities, access to financial resources and an increased ability to make decisions regarding their families. In addition, women from both communities felt that education has improved over time and that this represents a critical enabling factor to improving the quality of life among families and communities.

*“Twenty years back solely male were only the breadwinner of the family and female used to fully depend on male. But now the time has changed. Male and female are almost in the same level. They are equal. Female are also educated now and they are employed. They also earn, look after the family. If we (female) have money we can spend more on child care, buying varieties of nutritious food like meat, fruits, legumes etc. on our own. We should not wait our husband to buy anything (smiling)”-* P6: Mother of children born in 1995-2000, Dukuchhap

*“…Education is the key to any progress and improvement. It enhances the behaviours and improves our standard of living and the quality of our lives.” -* P6: Mother of children born between 2010-2015, Thecho

***Underlying Factors***

Improvements in food security, increased access to improved sources of water and sanitation, and increased accessibility and availability of health services in communities were identified as critical underlying factors to stunting reduction.

*Increased food security & feeding practices*

Women indicated that there were extensive periods of food insecurity, which may have influenced nutrition, from 1996 to 2006 due to armed conflict. In addition, geographic inaccessibility and lack of road and transportation infrastructure contributed to inadequate availability of food. After 2006, and the end of the civil war food security reportedly improved, and communities were able to access higher quality and nutritious foods.

*“…The year[s] between 1996-2006 was a stark reminder that the armed conflicts worsened the food and nutrition insecurity. Because of food and nutrition insecurity the major problem of chronic undernutrition was amplified. But also, a number of programs and policies in [the] nutrition and agriculture sector were formed and implemented in order to reduce the insecurity problem.”-* P10: Mother of children born between 2010-2015, Thecho

Some women indicated that infant feeding practices have improved over the last 20 years. Previously, family and work commitments prevented women from being able to exclusively breastfeed children for up to six months and that these commitments were both time consuming and exhausting. Changes in gender roles and norms, and increased prioritization of breastfeeding were outlined as important contributions to improved child nutrition.

*“ […] twenty years back there used to be heavy household chores and solely the woman was responsible for managing all the work at home along with caring for their family. I personally used to get exhaustion working all the day. Could not manage the time for breastfeeding to my first child. I used to leave him at home all the day and go to the paddy field early morning and come back late evening. But now the time has changed. Every woman provides the quality time and care to their children. They properly breastfeed their child until 6 months like I did to my last baby daughter (laughing).”* - P5: Mother of children born in 1995-2000, Dukuchhap

*Improved water and sanitation*

Women that gave birth between 1995-2000 and those that gave birth in 2010-2016 all noted that significant progress and achievements have been made to improve water, sanitation and hygiene over the last 20 years. These improvements were attributed to support from local municipalities and health posts to increase local access to toilets in communities and households. Community sensitization and increased promotion and awareness of hygiene and sanitation (e.g., handwashing and use of toilets) by community health workers and volunteers were key activities. Improved infrastructure, knowledge and changes in behaviour and social norms of hygiene and sanitation practices were reported to have contributed to declines in waterborne and infectious diseases.

*“…. people used to defecate in bushes and open land 20 years back. But now even the children are more alert and aware about hygiene and sanitation. They ask us to visit the toilet (smiling). All houses in our community have managed to have a toilet now via the help of the municipality and health post. Because of this also rarely do we suffer from waterborne disease like diarrhea (laughing).” -* P6: Mother of children born in 1995-2000, Dukuchhap

*“The FCHV and health in-charge ha[ve] spread the knowledge on [the] importance of maintaining hygiene and sanitation, hand washing and [the] use of toilets in our community, every household do have toilets in their house. The hygiene practice is very much improved now.”-* P8: Mother of children born between 2010-2015, Thecho

*Improved access to health services*

Improvements in the availability, accessibility and quality of health services delivered by skilled health providers were acknowledged by women in both communities and settings. More Government and private health facilities are now available in communities and efforts to improve quality of roads and transport (e.g., buses) have helped to facilitate local access and use of services. Women with children born in 2000s from the more rural community Dukucchap, indicated that despite substantial improvements in health services, continued progress is still needed to establish infrastructure and to ensure equitable access and use of health services for hard-to-reach communities, with geographically rough terrains.

*“The health facilities in the past used to have no medical equipment and professional personnel used to be very far, which is not the case today. Now the transports reach to the door of health institutions. Everything has improved with time. Availability of drugs and other services is noted to be continually provided to every sick patient with no age bar” -* P5: Mother of children born in 1995-2000, Dukuchhap

*“In [the] early days it was very difficult for us to reach the health facility. There was no mode of transport, no roads. We used to walk 4-6 hours a day for a health check-up. Though the bus has reached here the problem still exists. Only one bus used to do to-and-fro in our village but should say services has improved a lot compared to 20 years.”-* P9: Mother of children born in 1995-2000, Dukuchhap

***Immediate Causes***

Improved dietary intake and diversity, decreased prevalence of communicable diseases, decrease in low birthweight, and decreased fertility rates represented proximal factors that may have resulted in stunting reduction.

*Improved dietary intake*

Improved diet diversity and increased efforts to eat more nutritious foods were highlighted as trends observed by women in FGDs in both communities over the last two decades. Women in both groups felt that exclusive breastfeeding has increased, and that cultural and traditional infant feeding practices (e.g., discarding colostrum milk and feeding baby alcoholic beverages to sleep) are no longer employed. In addition, providing more diverse options for infants and making specific food for infants.

*“In past we used to discard the colostrum milk as it was considered to be unhealthy due to its yellow color. But now everyone in the community knows its importance and every mother feeds colostrum milk to her child.”-* P6: Mother of children born in 1995-2000, Dukuchhap

*“ […] twenty years back there used to be heavy household chores and solely the woman was responsible for managing all the work at home along with caring for their family. I personally used to get exhaustion working all the day. Could not manage the time for breastfeeding to my first child. I used to leave him at home all the day and go to the paddy field early morning and come back late evening. But now the time has changed. Every woman provides the quality time and care to their children. They properly breastfeed their child until 6 months like I did to my last baby daughter (laughing).”* - P5: Mother of children born in 1995-2000, Dukuchhap

*Decreased infection and diseases*

Medical and technological advances, including good coverage of childhood immunizations have helped to improve infectious/communicable diseases and resulted in dramatic improvements in overall health outcomes, including mortality and morbidity. Women from both communities indicated declines in infections, malaria, anaemia, and diarrhea, and that these diseases previously contributed to the poor nutritional status of children. Women explicitly outlined the importance of addressing infections among children, and that changes in these areas have contributed to gains in child growth.

*“…when children become unwell, it led to poor feeding habits, an infection directly reduced the benefits derived from the food, and children used to be untreated for a long time leading to their weight loss and ultimately lean and thin body. But now nutritious food is available to children, routine vaccination is done which prevents infectious diseases to them [more than] earlier.”* - P5: Mothers of children born in 1995-2000, Dukuchhap

*Maternal characteristics*

Greater spacing between pregnancies and births and decreased fertility rate and family sizes were associated with reductions in stunting among children, as accumulation of household wealth and ability to spend greater resources on fewer children were emphasized. Further, closely spaced births reportedly led to early cessation of breastfeeding for older children.

*“…giving birth every year leads to [a] family economic crisis as well. Poor families cannot afford quality food, shelter and health services. They need to struggle for their existence which has impacts on their health directly or indirectly.”* - P1: Mother of children born between 2010-2015, Thecho

*Child characteristics*

Increases in infants’ weight at birth was outlined by women in FGDs, and respondents felt that improved intrauterine growth were associated with improved access and use of health services by pregnant women, dietary intake during pregnancy, as well as improved respect and treatment by families.

*“The prevalence LBW babies were higher in early days, predominantly because of poor nutrition of the mother prior to and during pregnancy. Pregnant women were ignored and mistreated by their respective families in the past. They were often ignored with quality food, health care and love by their own husband and families which affected the child in the womb and born babies were mostly below normal weight. But now pregnant women are do provided with quality and timely health services, care and love from families and husband. Proper nutritious diet are well consumed by pregnant women these days.” -* P1: Mothers of children born in 1995-2000, Dukuchhap

**3. Community Health Worker Perspectives**

The Government of Nepal introduced Female Community Health Volunteers (FCHVs) in 1988, and this cadre initially focused on promotion and distribution of contraception. By 1991, 20,000 FCHVs had been trained (155) and as of 2017 there were an estimated 52,000 health workers providing critical community-based health services for at least 500 people or each ward of the village development committee (smallest administrative body) (156). FHCVs must be married and live in the community that they will serve, they are selected by community mother’s groups and receive training on reproductive, maternal, newborn and child health and nutrition (156). Their scope of practice has expanded over three decades and includes health promotion and distribution of resources and supplies (e.g., iron folate tablets, contraceptive methods, zinc tablets/oral rehydration solution), conduct mothers’ group meetings and lead counselling on birth preparedness. In addition, FCHVs support national public health campaigns (e.g., vitamin A distribution to under-fives) and potential diagnosis and treatment childhood diarrhea and referral of pneumonia cases (156). Some evidence is available demonstrating their importance in reducing maternal and newborn maternity (156), early pregnancy detection (157), as well as access to health services including antenatal services and immunization (155). Due to their critical role in providing community-based health services, a total of 10 health care workers, including Health In-Charges and Female Community Health Volunteers from Dukuchhap and Thecho in Godawari Municipality were recruited to participate in-depth interviews. These interviews helped to elicit key insights on local developments, enablers and challenges to progress in nutrition and stunting reduction in communities.

***Contextual Factors***

Women’s education and empowerment and increased remittances were identified by community health workers (e.g., health-in charge and FCHVs) were identified as critical enablers to the substantial reductions in stunting over time and improvements in chronic malnutrition among children.

*Women’s education and empowerment*

Community health workers in both communities indicated that female education at community level has increased substantially over time. Educating women has helped to improve their knowledge and practices regarding children’s nutrition, as well as understanding and utilization of local health services. Improvements in gender inequality were outlined with increased educational attainment, decreased child marriages and greater responsibilities and decision-making by women in families and households due to males traveling for employment.

*“...Education, especially among women, has improved over the years and has led to self-understanding about the availability and utilization of health services. Women’s education is a proven and important means of achieving gender equality, the effects of which are felt throughout families and communities if compared with the past.” -* Health Worker, Thecho

“*Now the adult males are in foreign employment so there are females leading the houses. Time has also changed and awareness has also increased. Now, females are also ahead in decision-making. In [the] past most of the female[s] were uneducated and men used to dominate them. But now females are also educated and they are doing well”*- Female Community Health Volunteer, Dukuchhap

*Remittances*

Poverty reduction and increased household income were largely attributed to remittances and labour migration. Remittances were seen as a critical livelihood strategy particularly in rural communities, and that these have substantially improved the socioeconomic status, accumulation of wealth and purchasing power of households. However, appropriate and effective use of remittances on food and to improve nutrition continues to represent an area of improvement.

*“Many people are abroad in this area. I think one from each house are far away from home. Because of this, it has increased the purchasing power of the family [and] has also improved the living standard here which was very pitiable 20 years back. But utilization of remittances only for food purpose[s] is not practiced much in our Nepali context. When it comes to food buying and consumption the money is not used properly.”* - Health Worker, Thecho HP

***Nutrition-specific and -sensitive programs/policies***

A substantial increase in nutrition-specific and –sensitive programs and policies was highlighted by community health workers in both communities. Financial and technical support from donors and development partners increased the number and capacity of skilled health providers, and substantially improved the availability of primary health services, including MNCH services (e.g., antenatal care, skilled delivery at birth, and postnatal care). Initiatives are now available to address chronic malnutrition among infants and children, immunization, weight monitoring, micronutrient supplementation (Baal Vita) and targeted feeding programs.

*“In [the] past there was no program on nutrition. Now we have treatment of malnourished babies. Immunization coverage has increased now. Vaccines has increased. In [the] past people used to ignore immunization program[s]. There was no facility for abortion in [the] past but there is [a] facility of abortion now. Weight monitoring is done here nowadays. In [the] past there [was] no[t] any supplement given to child. Nowadays the child is fed with Baal Vita supplement. Our health post house has become good with more equipment*.”- Female Community Health Volunteer from Thecho

*“Yes, many things have changed. Now the incentive given by [the] health post has increased for promotion of all ANC, PNC visit[s] and hospital delivery. In [the] past there was no birthing in this hospital but in [the] present here is [a] birthing facility. Now the pregnant women are given good counseling on nutrition and other health problems. Today there is provision of giving Baal Vita to the child up to 2 years. Now the staff of [the] health post are increased and are experienced. Now, education and training is given from health post, many NGOs/INGOs are working in nutrition. Recently Social Development and Promotion Center (SDPC) started treatment program for malnourished thin baby.”-* Female Community Health Volunteer from Dukuchap

***Underlying Causes***

Underlying factors at community level helped to address chronic malnutrition include increased access to health services and improved sources of water and increased sanitation.

*Increased access to health services*

Similarly to national stakeholders and mothers in communities, community health workers also indicated that the accessibility of health services has significant improved over the last two decades. Shorter distances to facilities, improved road infrastructure, and increased skilled health providers (including FCHVs) has improved the quality and quantity of health services available. This was associated with increased immunization coverage of children, improved MNCH services, micronutrient supplementation through community sensitization and reach by FCHVs.

*“In comparison to the past 20 years, everything has improved and developed. There has been an immense improvement in health service delivery. The health facilities are built within 30 minutes walking distance. Road accessibility has also improved and made it easier to reach the health institutions of even urban places (smiling).”* - Health worker, Dukuchhap

*Improved water and sanitation*

Implementation of WASH initiatives implemented by municipalities and donors in both communities have contributed to improvement in individual and community hygiene, as well as the surrounding environment. Building latrines, improved sources of water, and increased knowledge of sanitation and hygiene (e.g., handwashing) have helped to reduce stunting among children in Nepal.

*“WASH practices have improved over the period. The effect of WASH on nutrition was given a sort of comparatively low priority. An increment in the number of latrines has been happening over time. People had no access to hygienic water sources, improved latrines. They had no knowledge on the importance of hand washing. Municipalities, Gaunpalika [rural municipalities], health posts and different organizations helped us in building latrines, drinking water taps etc., so, our place is in the process of being declared as an open defecation free area (smiling).”* - Health Worker, Dukuchhap

***Immediate Causes***

Decreased burden of disease among children and improved dietary intake for both women and children (e.g., increased exclusive breastfeeding) were observed by community health workers.

*Increased dietary intake*

Improvements in exclusive breastfeeding and increased knowledge of nutrition were observed by community health workers as cultural and social norms and taboos regarding breastfeeding (e.g., avoidance of feeding colostrum to newborns due to colour) have been increasingly dispelled. These improvements were attributed to improved education, mass media and substantial breastfeeding promotion efforts by community health workers. The active involvement and dissemination of health messages by FCHV and community health workers has increased awareness of the importance of breastfeeding, timely initiative of complementary feeding, discouraging prelacteal feeding (feeding alternatives prior to breastfeeding) and sharing breastfeeding techniques. Further, Infant and Young Child Feeding (IYCF) programs reportedly had a substantial impact on changing health behaviours and practices in communities and community health workers indicated that efforts to ensure greater diversity and balance of diets has also contributed to improved nutrition at local levels.

*“In comparison to [the] past, now mothers are aware about their child’s nutrition and care practice[s]. We counsel them to feed the nutritious food, Baal Vita. Now the feeding pattern is also improved. They have started to give varieties of food to children. In [the] past they used to feed milk for [a] long time and they used to initiate the complementary feeding late. In past they used to feed same food which is consumed by adult[s]. Now we have trained them to prepare baby food like sarbottam pitho and they prepare it and feed to their baby. Now they prepare food including four group[s] of foods i.e. pulses, cereals, green vegetables, and food from animal origin.”-* Health Worker, Dukuchhap

*“In [the] past they didn’t know the techniques of breastfeeding. The babies were held haphazardly, not all [of the] black part of [the] nipple got into the baby[‘s] mouth, and they used to say that the milk produced by [the] mother is not sufficient. Now from the health post and FCHV[s] all mothers are trained on breastfeeding technique[s] and practices. At present they know how to breastf[e]ed their child. There was more child morbidity and mortality at that time. In [the] past, colostrum milk was not fed to the child but now they started to feed it. In [the] past there was no exclusive breastfeeding. [In the] past the mother used to go to work leaving their child at that time.”-* Female Community Health Volunteer, Dukuchhap

*“In past pregnant mother[s] were not given enough food to eat. There were cultural taboos like pregnant women should not eat papaya, green leafy vegetables, mother in law did not give much food to eat. They have to do heavy work during pregnancy. But now time[s] have changed; now [a] pregnant mother gets enough food to eat.”-* Female Community Health Worker, Thecho

***Conclusion***

Nepal’s decline in under-five stunting has been attributed to driving factors across multiple levels. Contextual factors are important to consider, such as political instability, the consolidation of democracy, improvements in education and women’s empowerment, the country's economic improvement, increased remittance income, and progress in national indicators, such as under-five mortality. An enabling political and policy environment was also necessary for a rapid and sustainable reduction in stunting. The role of nutrition-specific (e.g. food security, dietary diversity, breastfeeding, nutrition education) and nutrition-sensitive drivers (e.g. health sector reform, community-based health services, WASH, remittances, migration, urbanization) was highlighted by national stakeholders and community health workers and female community health volunteers. National stakeholders emphasized the critical role of multi-sectoral approach to nutrition and the prioritization of strengthening ground level capacity, including provision of health services in communities. These programs were coordinated and led in a systematic manner by respected national policymakers and champions with the power to advocate for nutrition at all levels of government, creating space for leadership at all levels of governance. There was also a highly synergistic environment, with the impetus for political action occurring at both the central and state level. The successful experiences carried out by government, ministries, external development partners, local and international NGOs, a vibrant civil society and nutrition stakeholders laid the foundation for a decline in under-five stunting.

# **Acknowledgments**

The authors would like to sincerely thank their Technical Advisory Group for oversight throughout the research process, including Dr. Zulfiqar Bhutta, (The Hospital for Sick Children), Shawn Baker (former Bill and Melinda Gates Foundation), Ellen Piwoz (Bill and Melinda Gates Foundation), Dr. Robert Black (Johns Hopkins University), Dr. Sue Horton (University of Waterloo), Dr. Joanne Katz (Johns Hopkins University), Dr. Purnima Menon (International Food Policy Research Institute), Dr. Meera Shekar (The World Bank), and Dr. Cesar Victora (Federal University of Pelotas). Special thanks to Kevin Ho, Dr. Oliver Rothschild and Dr. Niranjan Bose from Gates Ventures for funding support and overall technical/research support to the project. Acknowledgement also goes to Tyler Vaivada and Aviva Rappaport for research support throughout the case study. A heartfelt acknowledgement goes to all participants of our research including the national stakeholders and community members that we interviewed.

The authors’ responsibilities were as follows: NA, MI, JW, SB, HT, KC: analysis; All authors contributed to interpretation of the data; KC, NA, JW: drafting of the manuscript; NA and ZAB: study design; NA, KC, RKS, MM, and ZAB: critical revision of the manuscript; ZAB: primary responsibility for the final content; and all authors: read and approved the final manuscript. None of the authors have any conflicts of interest to declare.

## **Supplementary Appendices References**

1. World Bank. GDP per capita, PPP (constant 2011 international $) [Internet]. Open Data. 2018. Available from: https://data.worldbank.org/indicator/NY.GDP.PCAP.PP.CD?locations=NP

2. The World Bank. Poverty headcount ratio at $1.90 a day (2011 PPP) (% of population) | Data [Internet]. 2019 [cited 2019 Feb 19]. Available from: https://data.worldbank.org/indicator/SI.POV.DDAY?locations=NP

3. The World Bank. Urban population (% of total) | Data [Internet]. 2019 [cited 2019 Mar 7]. Available from: https://data.worldbank.org/indicator/SP.URB.TOTL.IN.ZS?locations=NP

4. The World Bank. Literacy rate, adult total (% of people ages 15 and above) | Data [Internet]. Open Data . 2018 [cited 2018 Sep 6]. Available from: https://data.worldbank.org/indicator/SE.ADT.LITR.ZS?locations=NP&view=chart

5. World Bank. Literacy rate, adult female (% of females ages 15 and above) | Data [Internet]. 2019 [cited 2019 Feb 25]. Available from: https://data.worldbank.org/indicator/SE.ADT.LITR.FE.ZS?locations=NP

6. UNDP. Human Development Reports: Gender Development Index (GDI) [Internet]. 2018 [cited 2019 Feb 25]. Available from: http://hdr.undp.org/en/indicators/137906#

7. WHO UNICEF. JMP [Internet]. 2019 [cited 2019 Feb 25]. Available from: https://washdata.org/data/household#!/npl

8. World Bank. People practicing open defecation (% of population) | Data [Internet]. 2019 [cited 2019 Feb 25]. Available from: https://data.worldbank.org/indicator/SH.STA.ODFC.ZS?locations=NP

9. Black RE, Allen LH, qar Bhutta ZA, Caulfi eld LE, de Onis M, Ezzati M, Mathers C, Rivera J. Maternal and Child Undernutrition 1 Maternal and child undernutrition: global and regional exposures and health consequences. Lancet [Internet]. 2008 [cited 2018 Aug 21];371:243–60. Available from: www.thelancet.com

10. Central Bureau of Statistics. National Population and Housing Census 2011 (National Report) [Internet]. 2012. Available from: http://cbs.gov.np/image/data/Population/National Report/National Report.pdf

11. UNFPA Nepal. Population Situation Analysis of Nepal (With Respect to Sustainable Development) [Internet]. 2017. Available from: https://www.unfpa.org/

12. Devkota MD, Adhikari RK, Upreti SR. Stunting in Nepal: Looking back, looking ahead. Matern Child Nutr. 2016;12:257–9.

13. Shively G, Gars J, Sununtnasuk C. A review of food security and human nutrition issues in Nepal [Internet]. West Lafayette, Indiana; 2011. Available from: http://ageconsearch.umn.edu/bitstream/116190/2/11-5.pdf

14. Gurung G. Child health status of Nepal: Social exclusion perspective. J Nepal Paediatr Soc. 2009;29:79–84.

15. Nisar Y Bin, Dibley MJ, Aguayo VM. Iron-folic acid supplementation during pregnancy reduces the risk of stunting in children less than 2 years of age: A retrospective cohort study from Nepal. Nutrients. 2016;8.

16. Thapa M, Neopane AK, Singh UK, Aryal N, Agrawal K, Shrestha B. Nutritional status of children in two districts of the mountain region of Nepal. J Nepal Health Res Counc [Internet]. 2013;11:235–9. Available from: http://libaccess.mcmaster.ca/login?url=http://ovidsp.ovid.com/ovidweb.cgi?T=JS&CSC=Y&NEWS=N&PAGE=fulltext&D=emed15&AN=604670963

17. Gaire S, Delbiso TD, Pandey S, Guha-Sapir D. Impact of disasters on child stunting in Nepal. Risk Manag Healthc Policy [Internet]. 2016;9:113–27. Available from: http://libaccess.mcmaster.ca/login?url=http://ovidsp.ovid.com/ovidweb.cgi?T=JS&CSC=Y&NEWS=N&PAGE=fulltext&D=prem&AN=27354834

18. Shively G, Sununtnasuk C. Agricultural diversity and child stunting in Nepal. (Special Issue: Farm-level pathways to improved nutritional status.). J Dev Stud [Internet]. Department of Agricultural Economics, Purdue University, West Lafayette, Indiana, USA. shivelyg@purdue.edu; 2015;51:1078–96. Available from: http://www.tandfonline.com/loi/fjds20 http://ovidsp.ovid.com/ovidweb.cgi?T=JS&CSC=Y&NEWS=N&PAGE=fulltext&D=caba6&AN=20153324888 http://bf4dv7zn3u.search.serialssolutions.com.myaccess.library.utoronto.ca/?url_ver=Z39.88-2004&rft_val_fmt=info:ofi/fmt:kev:mt

19. Fang C, Sharma R, Favre R, Hollema S. Special Report: FAO/WFP Food Security Assessment Mission in Nepal [Internet]. 2007. Available from: www.fao.orgatthefollowingurladdress:http://www.fao.org/giews/

20. Shively GE. Infrastructure mitigates the sensitivity of child growth to local agriculture and rainfall in Nepal and Uganda. Proc Natl Acad Sci [Internet]. 2017;114:903–8. Available from: http://www.pnas.org/lookup/doi/10.1073/pnas.1524482114

21. Tiwari S, Jacoby HG, Skoufias E. Monsoon Babies: Rainfall Shocks and Child Nutrition in Nepal. Econ Dev Cult Change [Internet]. 2017;65:167–88. Available from: http://www.journals.uchicago.edu/doi/10.1086/689308

22. Brainerd E, Menon N. Religion and health in early childhood: evidence from South Asia. Popul Dev Rev. 2015;41:439–63.

23. Ministry of Health and Population Nepal, Partnership for Maternal, Newborn & Child Health, WHO WB and A for HP and SR. Success Factors for Women’s and Children’s Health, NEPAL. [Internet]. Bulletin of the World Health Organization. Geneva; 2014. Available from: http://www.who.int/pmnch/knowledge/publications/nepal_country_report.pdf

24. Cunningham K, Headey D, Singh A, Karmacharya C, Rana PP. Maternal and Child Nutrition in Nepal: Examining drivers of progress from the mid-1990s to 2010s. Glob Food Sec [Internet]. Elsevier; 2016;0–1. Available from: http://dx.doi.org/10.1016/j.gfs.2017.02.001

25. Headey DD, Hoddinott J. Understanding the Rapid Reduction of Undernutrition in Nepal, 2001–2011. Baud O, editor. PLoS One [Internet]. 2015 [cited 2018 Mar 19];10:1–13. Available from: http://dx.plos.org/10.1371/journal.pone.0145738

26. UNICEF. Situation of children and women in Nepal [Internet]. Kathmandu, Nepal; 2006. Available from: https://www.unicef.org/Nepal_SitAn_2006.pdf

27. Partap U, Hill DR. The Maoist insurgency (1996-2006) and child health indicators in Nepal. Int Health [Internet]. Royal Society of Tropical Medicine and Hygiene; 2012;4:135–42. Available from: http://dx.doi.org/10.1016/j.inhe.2011.12.004

28. World Health Organization. Health financing profile 2017 [Internet]. 2017. Available from: https://data.worldbank.org/indicator/,

29. The World Bank. Current health expenditure per capita (current US$) | Data [Internet]. Open Data . 2018 [cited 2018 Aug 29]. Available from: https://data.worldbank.org/indicator/SH.XPD.CHEX.PC.CD?locations=NP

30. Uematsu H, Shidiq RA, Tiwari S. Trends and Drivers of Poverty Reduction in Nepal A Historical Perspective [Internet]. 2016. Available from: http://econ.worldbank.org.

31. Kansakar VBS. International Migration and Citizenship in Nepal. Population Monograph of Nepal [Internet]. Kathmandu, Nepal; 2003 [cited 2018 Aug 21]. p. 85–119. Available from: http://cbs.gov.np/image/data/Population/Monograph_vol_1_2(1-10,11-21)/Chapter 14 International Migration and Citizenship in Nepal.pdf

32. Central Bureau of Statistics. Nepal Living Standards Survey 2010/2011 [Internet]. 2011. Available from: http://cbs.gov.np/image/data/Surveys/Nepal Living Standard Survey 2010_11-English/Statistical_Report_Vol1.pdf

33. Osei A, Pandey P, Spiro D, Nielson J, Shrestha R, Talukder Z, Quinn V, Haselow N. Household food insecurity and nutritional status of children aged 6 to 23 months in Kailali District of Nepal. Food Nutr Bull [Internet]. 2010;31:483–94. Available from: http://nsinf.publisher.ingentaconnect.com/content/nsinf/fnb/2010/00000031/00000004/art00002

34. Niraula SR, Barnwal SP, Paudel S, Mishra S, Dahal S, Das S, Pradhan S, Ghimire S, Khanal S, Sharma S, et al. Prevalence and associated risk factors with malnutrition among under-five Nepalese children of Borbote village, Ilam. Heal Renaiss [Internet]. 2013;11:111–8. Available from: http://www.nepjol.info/index.php/HREN/article/view/8217/6682

35. Pradhan A. Fitting ordinal regression analysis to anthropometric data. J Nepal Health Res Counc [Internet]. Pradhan, A. Department of Community Medicine, KIST Medical College, Lalitpur, Nepal. amiseason@yahoo.com; 2011;9:61–6. Available from: http://libaccess.mcmaster.ca/login?url=http://ovidsp.ovid.com/ovidweb.cgi?T=JS&CSC=Y&NEWS=N&PAGE=fulltext&D=med7&AN=22929716 http://sfx.scholarsportal.info/mcmaster?sid=OVID:medline&id=pmid:22929716&id=doi:&issn=1727-5482&isbn=&volume=9&issue=1&spage=61&p

36. Dorsey JL, Manohar S, Neupane S, Shrestha B, Klemm RDW, West KP. Individual, household, and community level risk factors of stunting in children younger than 5 years: Findings from a national surveillance system in Nepal. Matern Child Nutr [Internet]. (Dorsey, Manohar, Klemm, West) Center for Human Nutrition, Department of International Health, Bloomberg School of Public Health Johns Hopkins University Baltimore, Maryland USA (Neupane, Shrestha) PoSHAN Study Team Johns Hopkins University Kathmandu Nepa; 2017; Available from: http://onlinelibrary.wiley.com/journal/10.1111/(ISSN)1740-8709 http://libaccess.mcmaster.ca/login?url=http://ovidsp.ovid.com/ovidweb.cgi?T=JS&CSC=Y&NEWS=N&PAGE=fulltext&D=emexa&AN=614571679 http://sfx.scholarsportal.info/mcmaster?sid=OVID:embase&id=pmid:&

37. Kim R, Mejia-Guevara I, Corsi DJ, Aguayo VM, Subramanian S V. Relative importance of 13 correlates of child stunting in South Asia: insights from nationally representative data from Afghanistan, Bangladesh, India, Nepal, and Pakistan. Soc Sci Med [Internet]. Department of Social and Behavioral Sciences, Harvard T.H. Chan School of Public Health, Cambridge, MA 02138, USA. svsubram@hsph.harvard.edu; 2017; Available from: http://www.sciencedirect.com/science/journal/02779536 http://ovidsp.ovid.com/ovidweb.cgi?T=JS&CSC=Y&NEWS=N&PAGE=fulltext&D=caba6&AN=20173263878 http://bf4dv7zn3u.search.serialssolutions.com.myaccess.library.utoronto.ca/?url_ver=Z39.88-2004&rft_val_fmt=inf

38. Tiwari R, Ausman LM, Agho KE. Determinants of stunting and severe stunting among under-fives: evidence from the 2011 Nepal Demographic and Health Survey. BMC Pediatr [Internet]. Nutrition Promotion and Consultancy Service, Kathmandu, Nepal. rinatiwari@hotmail.com; 2014;14. Available from: http://www.biomedcentral.com/content/pdf/1471-2431-14-239.pdf http://ovidsp.ovid.com/ovidweb.cgi?T=JS&CSC=Y&NEWS=N&PAGE=fulltext&D=caba6&AN=20143375378 http://bf4dv7zn3u.search.serialssolutions.com.myaccess.library.utoronto.ca/?url_ver=Z39.88-2004&rft_val

39. Devakumar D, Kular D, Shrestha BP, Grijalva-Eternod C, Daniel RM, Saville NM, Manandhar DS, Costello A, Osrin D, Wells JCK. Socioeconomic determinants of growth in a longitudinal study in Nepal. Matern Child Nutr [Internet]. Devakumar, Delan. Institute for Global Health, UCL, London, UK. Kular, Dalvir. Institute for Global Health, UCL, London, UK. Shrestha, Bhim P. Mother and Infant Research Activities, Kathmandu, Nepal. Grijalva-Eternod, Carlos. Institute for Global Health, ; 2017;27. Available from: http://libaccess.mcmaster.ca/login?url=http://ovidsp.ovid.com/ovidweb.cgi?T=JS&CSC=Y&NEWS=N&PAGE=fulltext&D=medp&AN=28449415 http://sfx.scholarsportal.info/mcmaster?sid=OVID:medline&id=pmid:28449415&id=doi:10.1111%2Fmcn.12462&issn=1740-8695&isbn=&volume=&

40. Headey DD, Hoddinott J. Understanding the Rapid Reduction of Undernutrition in Nepal. PLoS One [Internet]. 2015 [cited 2018 Jan 26];10:e0145738. Available from: http://journals.plos.org/plosone/article/file?id=10.1371/journal.pone.0145738&type=printable

41. Headey D, Hoddinott J, Park S. Accounting for nutritional changes in six success stories: A regression- decomposition approach. Glob Food Sec [Internet]. 2017 [cited 2018 Jan 26];13:12–20. Available from: https://ac.els-cdn.com/S2211912416300992/1-s2.0-S2211912416300992-main.pdf?_tid=9209ebf8-02c0-11e8-b418-00000aab0f6c&acdnat=1516988862_c5e6fc22372b9d1e863bd7c2f40686bc

42. Headey D, Hoddinott J, Park S. Drivers of nutritional change in four South Asian countries: A dynamic observational analysis. Matern Child Nutr. 2016;12:210–8.

43. Krishna A, Mejía-Guevara I, McGovern M, Aguayo V, Subramanian S V. Trends in inequalities in child stunting in South Asia. Matern Child Nutr [Internet]. 2017;e12517. Available from: http://doi.wiley.com/10.1111/mcn.12517%0Ahttp://www.ncbi.nlm.nih.gov/pubmed/29048726

44. Restrepo-Méndez MC, Barros AJ, Black RE, Victora CG. Time trends in socio-economic inequalities in stunting prevalence: analyses of repeated national surveys. Public Health Nutr [Internet]. 2015;18:2097–104. Available from: http://www.journals.cambridge.org/abstract_S1368980014002924

45. Joshi AR. Maternal schooling and child health: preliminary analysis of the intervening mechanisms in rural Nepal. Health Transit Rev [Internet]. (Joshi) The World Bank, Washington, DC 20433. A.R. Joshi, The World Bank, Washington, DC 20433.; 1994;4:1–28. Available from: http://libaccess.mcmaster.ca/login?url=http://ovidsp.ovid.com/ovidweb.cgi?T=JS&CSC=Y&NEWS=N&PAGE=fulltext&D=emed6&AN=24940123 http://sfx.scholarsportal.info/mcmaster?sid=OVID:embase&id=pmid:10147162&id=doi:&issn=1036-4005&isbn=&volume=4&issue=1&spage=1&pa

46. Miller LC, Joshi N, Lohani M, Rogers B, Mahato S, Ghosh S, Webb P. Women’s education level amplifies the effects of a livelihoods-based intervention on household wealth, child diet, and child growth in rural Nepal. Int J Equity Health [Internet]. Miller, Laurie C. Department of Pediatrics, Tufts University, Boston, MA, USA. Laurie.miller@tufts.edu. Joshi, Neena. Heifer Nepal, Kathmandu, Nepal. Lohani, Mahendra. Heifer International, Little Rock, AR, USA. Rogers, Beatrice. Friedman School of Nutrit; 2017;16:183. Available from: http://libaccess.mcmaster.ca/login?url=http://ovidsp.ovid.com/ovidweb.cgi?T=JS&CSC=Y&NEWS=N&PAGE=fulltext&D=prem&AN=29047376 http://sfx.scholarsportal.info/mcmaster?sid=OVID:medline&id=pmid:29047376&id=doi:10.1186%2Fs12939-017-0681-0&issn=1475-9276&isbn=&

47. Dancer D, Anu R. Maternal autonomy and child nutrition: evidence from rural Nepal. Indian Growth Dev Rev [Internet]. 2009;2:18–38. Available from: http://www.emeraldinsight.com/Insight/viewContentItem.do

48. Sarki M, Robertson A, Parlesak A. Association between socioeconomic status of mothers, food security, food safety practices and the double burden of malnutrition in the Lalitpur district, Nepal. Arch Public Heal [Internet]. 2016;74. Available from: http://download.springer.com/static/pdf/798/art%253A10.1186%252Fs13690-016-0150-z.pdf?originUrl=http%3A%2F%2Farchpublichealth.biomedcentral.com%2Farticle%2F10.1186%2Fs13690-016-0150-z&token2=exp=1474618668~acl=%2Fstatic%2Fpdf%2F798%2Fart%25253A10.1186%252

49. Gaurav K, Poudel IS, Bhattarai S, Pradhan PMS, Pokharel PK. Malnutrition status among under-5 children in a hill community of Nepal. Kathmandu Univ Med J [Internet]. 2014;12:264–8. Available from: http://www.kumj.com.np/issue/48/264-268.pdf

50. The World Bank. Basic and Primary Education Project [Internet]. 1999. Available from: http://documents.worldbank.org/curated/en/273221468779978360/pdf/multi-page.pdf

51. Cunningham, K. et al. Reaching New Heights: 20 Years of Nutrition Progress in Nepal. 2011;115–24.

52. Nepal Law Commission. Non-Formal Education Policy, 2063 B.S. (2007 A.D.) [Internet]. 2007. Available from: www.lawcommission.gov.np

53. Eklund P, Imai K, Felloni F. Women’s organisations, maternal knowledge, and social capital to reduce prevalence of stunted children: evidence from rural Nepal. J Dev Stud [Internet]. 2007;43:456–89. Available from: http://www.informaworld.com/smpp/content~content=a773457964~db=all~order=page

54. Cunningham K, Ploubidis GB, Purnima M, Ruel M, Suneetha K, Uauy R, Ferguson E. Women’s empowerment in agriculture and child nutritional status in rural Nepal. Public Health Nutr [Internet]. Department of Population Health, Faculty of Epidemiology, London School of Hygiene and Tropical Medicine, Keppel Street, London WC1E 7HT, UK. kendacunningham@gmail.com; 2015;18:3134–45. Available from: http://journals.cambridge.org/action/displayJournal?jid=PHN http://ovidsp.ovid.com/ovidweb.cgi?T=JS&CSC=Y&NEWS=N&PAGE=fulltext&D=caba6&AN=20153400706 http://bf4dv7zn3u.search.serialssolutions.com.myaccess.library.utoronto.ca/?url_ver=Z39.88-2004&rft_val_f

55. Malapit HJL, Suneetha K, Quisumbing AR, Cunningham K, Tyagi P. Women’s empowerment mitigates the negative effects of low production diversity on maternal and child nutrition in Nepal. (Special Issue: Farm-level pathways to improved nutritional status.). J Dev Stud [Internet]. International Food Policy Research Institute, Poverty, Health, and Nutrition Division, 2033 K Street, NW, Washington, Dist. of Columbia, USA. h.malapit@cgiar.org; 2015;51:1097–123. Available from: http://www.tandfonline.com/loi/fjds20 http://ovidsp.ovid.com/ovidweb.cgi?T=JS&CSC=Y&NEWS=N&PAGE=fulltext&D=caba6&AN=20153324889 http://bf4dv7zn3u.search.serialssolutions.com.myaccess.library.utoronto.ca/?url_ver=Z39.88-2004&rft_val_fmt=info:ofi/fmt:kev:mt

56. The World Bank Group. Moving up the Ladder: Poverty Reduction and Social Mobility in Nepal. 2016;1–88.

57. Central Bureau of Statistics. Annual Household Survey 2015/16 (Major Findings) [Internet]. 2016. Available from: https://reliefweb.int/sites/reliefweb.int/files/resources/Annual Household Survey 2015_16_Major findings.pdf

58. Central Bureau of Statistics. Nepal in Figures 2018 [Internet]. 2018. Available from: www.cbs.gov.np

59. NPC, CBS, WFP, World Bank, UNICEF. Nepal Thematic Report on Food Security and Nutrition 2013. 2013;99. Available from: http://documents.wfp.org/stellent/groups/public/documents/ena/wfp256518.pdf?_ga=1.182793378.523972516.1476818874

60. Feed the Future Innovation Laboratory for Collaborative Research for Nutrition. Baseline Summary Report 1st Annual Panel Survey 2013: Policy and Science of Health, Agriculture and Nutrition (PoSHAN) Community Studies. 2016;1–106.

61. Gillespie S, Hodge J, Yosef S, Pandya-Lorch R. Nourishing Millions: Stories of Change in Nutrition [Internet]. 2016. Available from: http://ebrary.ifpri.org/utils/getfile/collection/p15738coll2/id/130395/filename/130606.pdf

62. Paudel R, Pradhan B, Wagle RR, Pahari DP, Onta SR. Risk factors for stunting among children: a community based case control study in Nepal. Kathmandu Univ Med J [Internet]. 2012;10:18–24. Available from: http://www.kumj.com.np/issue/39/18-24.pdf

63. Sreeramareddy CT, Ramakrishnareddy N, Mayoori S. Association between household food access insecurity and nutritional status indicators among children aged <5 years in Nepal: results from a national, cross-sectional household survey. Public Health Nutr [Internet]. Department of Population Medicine, Faculty of Medicine and Health Sciences, Universiti Tunku Abdul Rahman, Jalan Sungai Long, Bandar Sungai Long, Post code: 43000, Kajang, Cheras, Selangor, Malaysia. csts74@hotmail.com; 2015;18:2906–14. Available from: http://journals.cambridge.org/action/displayJournal?jid=PHN http://ovidsp.ovid.com/ovidweb.cgi?T=JS&CSC=Y&NEWS=N&PAGE=fulltext&D=caba6&AN=20153365880 http://bf4dv7zn3u.search.serialssolutions.com.myaccess.library.utoronto.ca/?url_ver=Z39.88-2004&rft_val_f

64. Psaki S, Bhutta ZA, Ahmed T, Shamsir A, Bessong P, Munirul I, John S, Kosek M, Lima A, Nesamvuni C, et al. Household food access and child malnutrition: results from the eight-country MAL-ED study. Popul Health Metr [Internet]. 2012;10. Available from: http://www.pophealthmetrics.com/content/10/1/24/abstract

65. Parajuli D, Acharya G. Impact of social fund on the welfare of rural households: evidence from the Nepal poverty alleviation fund [Internet]. World Bank Policy Research Working Paper …. 2012. Report No.: 6042. Available from: http://papers.ssrn.com/sol3/papers.cfm?abstract_id=2043470

66. Miller LC, Joshi N, Lohani M, Rogers B, Loraditch M, Houser R, Singh P, Mahato S. Community development and livestock promotion in rural Nepal: effects on child growth and health. Food Nutr Bull [Internet]. 2014;35:312–26. Available from: http://www.ingentaconnect.com/content/nsinf/fnb/2014/00000035/00000003/art00004 http://ovidsp.ovid.com/ovidweb.cgi?T=JS&CSC=Y&NEWS=N&PAGE=fulltext&D=caba6&AN=20143343465 http://bf4dv7zn3u.search.serialssolutions.com.myaccess.library.utoronto.ca/?url_ver=Z

67. Osei A, Pandey P, Nielsen J, Pries A, Spiro D, Davis D, Quinn V, Haselow N. Combining home garden, poultry, and nutrition education program targeted to families with young children improved anemia among children and anemia and underweight among nonpregnant women in Nepal. Food Nutr Bull [Internet]. Department of Social Affairs, African Union Commission, Room 1216, New Building, PO Box 3243, Addis Ababa, Ethiopia. andykofi20@gmail.com oseia@africa-union.org; 2017;38:49–64. Available from: http://journals.sagepub.com/loi/fnb http://ovidsp.ovid.com/ovidweb.cgi?T=JS&CSC=Y&NEWS=N&PAGE=fulltext&D=caba6&AN=20173176648 http://bf4dv7zn3u.search.serialssolutions.com.myaccess.library.utoronto.ca/?url_ver=Z39.88-2004&rft_val_fmt=info:ofi/fmt:kev:mtx:

68. Chaparro, C.; Oot, L.; Sethuraman K. Nepal Nutrition Profile. 2014;1–8.

69. SUN Movement. Nepal. 2014;84–7.

70. The DHS Program. STATcompiler [Internet]. Nepal - Breastfeeding. 2016. Available from: https://www.statcompiler.com/en/

71. Singh GCP, Manju N, Grubesic RB, Connell FA. Factors associated with underweight and stunting among children in rural Terai of Eastern Nepal. Asia Pacific J Public Heal [Internet]. 2009;21:144–52. Available from: http://aph.sagepub.com/cgi/content/abstract/21/2/144

72. Kattel S, McNeil N, Tongkumchum P. Social determinants of linear growth among under five years children in Nepal. Pertanika J Soc Sci Humanit [Internet]. Departments of Mathematics and Computer Science, Faculty of Science and Technology, Prince of Songhkla University, Pattani Campus, Thailand. sumitrakattel@gmail.com nittaya.ch@psu.ac.th phattrawan@gmail.com; 2017;25:851–9. Available from: http://www.pertanika.upm.edu.my/Pertanika PAPERS/JSSH Vol. 25 (2) Jun. 2017/21 JSSH-1551-2016-3rdProof.pdf http://ovidsp.ovid.com/ovidweb.cgi?T=JS&CSC=Y&NEWS=N&PAGE=fulltext&D=caba6&AN=20173288914 http://bf4dv7zn3u.search.serialssolutions.com.myaccess.lib

73. Panter-Brick C. Seasonal growth patterns in rural Nepali children. Ann Hum Biol [Internet]. 1997;24:1–18. Available from: http://libaccess.mcmaster.ca/login?url=http://ovidsp.ovid.com/ovidweb.cgi?T=JS&CSC=Y&NEWS=N&PAGE=fulltext&D=emed7&AN=27399238 http://sfx.scholarsportal.info/mcmaster?sid=OVID:embase&id=pmid:9022902&id=doi:&issn=0301-4460&isbn=&volume=24&issue=1&spage=1&pa

74. Shrestha B. Nutritional status of under-five children in western Nepal. J Nepal Paediatr Soc [Internet]. Department of Paediatrics, Gandaki Medical College Teaching Hospital and Research Centre, Pokhara, Nepal. bandana139@yahoo.com; 2014;34:119–24. Available from: http://www.nepjol.info/index.php/JNPS/article/view/10566/9226 http://ovidsp.ovid.com/ovidweb.cgi?T=JS&CSC=Y&NEWS=N&PAGE=fulltext&D=caba6&AN=20143403845 http://bf4dv7zn3u.search.serialssolutions.com.myaccess.library.utoronto.ca/?url_ver=Z39.88-2004&rft_val

75. Lamichhane DK, Leem JH, Kim HC, Park MS, Lee JY, Moon SH, Ko JK. Association of infant and young child feeding practices with under-nutrition: evidence from the Nepal Demographic and Health Survey. Paediatr Int Child Heal [Internet]. Lamichhane, Dirga Kumar. a Departments of Social and Preventive Medicine , School of Medicine, Inha University , Incheon , Korea. Leem, Jong Han. a Departments of Social and Preventive Medicine , School of Medicine, Inha University , Incheon , Korea. Leem; 2016;36:260–9. Available from: http://libaccess.mcmaster.ca/login?url=http://ovidsp.ovid.com/ovidweb.cgi?T=JS&CSC=Y&NEWS=N&PAGE=fulltext&D=medc&AN=26863233 http://sfx.scholarsportal.info/mcmaster?sid=OVID:medline&id=pmid:26863233&id=doi:10.1080%2F20469047.2015.1109281&issn=2046-9047&is

76. Busert LK, Neuman M, Rehfuess EA, Sophiya D, Harthan J, Chaube SS, Bishnu B, Costello H, Costello A, Manandhar DS, et al. Dietary diversity is positively associated with deviation from expected height in rural Nepal. J Nutr [Internet]. Institute for Medical Informatics, Biometry, and Epidemiology, Ludwig-Maximilians-Universitat Munchen, Munich, Germany. laura.busert@gmail.com; 2016;146:1387–93. Available from: http://jn.nutrition.org/content/146/7/1387.full http://ovidsp.ovid.com/ovidweb.cgi?T=JS&CSC=Y&NEWS=N&PAGE=fulltext&D=caba6&AN=20163248704 http://bf4dv7zn3u.search.serialssolutions.com.myaccess.library.utoronto.ca/?url_ver=Z39.88-2004&rft_val_fmt=info:ofi/

77. Poudel KC, Nakahara S, Okumura J, Wakai S. Day-care centre supplementary feeding effects on child nutrition in urban slum areas of Nepal. J Trop Pediatr [Internet]. Department of International Community Health, Graduate School of Medicine, The University of Tokyo, 7-3-1 Hongo, Bunkyo-ku, Tokyo 113-0033, Japan. shinji@m.u-tokyo.ac.jp; 2004;50:116–9. Available from: http://ovidsp.ovid.com/ovidweb.cgi?T=JS&CSC=Y&NEWS=N&PAGE=fulltext&D=caba5&AN=20043056842 http://bf4dv7zn3u.search.serialssolutions.com.myaccess.library.utoronto.ca/?url_ver=Z39.88-2004&rft_val_fmt=info:ofi/fmt:kev:mtx:journal&rfr_id=info:sid/Ovid:caba5&r

78. Yadav DK, Gupta N, Shrestha N, Kumar A, Bose DK. Community based nutrition education for promoting nutritional status of children under three years of age in rural areas of Mahottari district of Nepal. J Nepal Paediatr Soc [Internet]. School of Health and Allied Sciences, Pokhara University, Kaski, Nepal. dipendrayadavph@gmail.com; 2014;34:181–7. Available from: http://www.nepjol.info/index.php/JNPS/article/view/10286/10051 http://ovidsp.ovid.com/ovidweb.cgi?T=JS&CSC=Y&NEWS=N&PAGE=fulltext&D=caba6&AN=20153168015 http://bf4dv7zn3u.search.serialssolutions.com.myaccess.library.utoronto.ca/?url_ver=Z39.88-2004&rft_va

79. West KP, LeClerq SC, Shrestha SR, Wu LS, Pradhan EK, Khatry SK, Katz J, Adhikari R, Sommer A. Effects of vitamin A on growth of vitamin A-deficient children: field studies in Nepal. J Nutr. 1997;127:1957–65.

80. Pokhrel K, Nanishi K, Poudel KC, Pokhrel KG, Tiwari K, Jimba M. Undernutrition Among Infants and Children in Nepal: Maternal Health Services and Their Roles to Prevent it. Matern Child Heal J [Internet]. 2016;20:2037–49. Available from: http://libaccess.mcmaster.ca/login?url=http://ovidsp.ovid.com/ovidweb.cgi?T=JS&CSC=Y&NEWS=N&PAGE=fulltext&D=prem&AN=27236701

81. Langford R, Lunn P, Panter-Brick C. Hand-washing, subclinical infections, and growth: a longitudinal evaluation of an intervention in Nepali slums. Am J Hum Biol [Internet]. Langford, Rebecca. School of Social and Community Medicine, University of Bristol, Canynge Hall, Bristol, BS8 2PS, UK. beki.langford@bristol.ac.uk; 2011;23:621–9. Available from: http://libaccess.mcmaster.ca/login?url=http://ovidsp.ovid.com/ovidweb.cgi?T=JS&CSC=Y&NEWS=N&PAGE=fulltext&D=med7&AN=21630368 http://sfx.scholarsportal.info/mcmaster?sid=OVID:medline&id=pmid:21630368&id=doi:10.1002%2Fajhb.21189&issn=1042-0533&isbn=&volume=

82. Panter-Brick C, Lunn PG, Langford RM, Makhan M, Manandhar DS. Pathways leading to early growth faltering: an investigation into the importance of mucosal damage and immunostimulation in different socio-economic groups in Nepal. Br J Nutr [Internet]. Department of Anthropology, Durham University, 43 Old Elvet, Durham DH1 3HN, UK. catherine.panter-brick@durham.ac.uk; 2009;101:558–67. Available from: http://journals.cambridge.org/action/displayAbstract?fromPage=online&aid=3870372&fulltextType=RA&fileId=S000711450802744X http://ovidsp.ovid.com/ovidweb.cgi?T=JS&CSC=Y&NEWS=N&PAGE=fulltext&D=caba6&AN=20093108311 http://bf4dv7zn3u.search.serialssolutions.c

83. Vaidya A, Saville N, Shrestha B, Costello A, Manandhar D, Osrin D. Effects of antenatal multiple micronutrient supplementation on children’s weight and size at 2 years of age in Nepal: follow-up of a double-blind randomised controlled trial [Internet]. Lancet (london, england). 2008. p. 492–9. Available from: http://onlinelibrary.wiley.com/o/cochrane/clcentral/articles/976/CN-00629976/frame.html

84. Sharma KR. Farm commercialization and nutritional status of children: the case of the vegetables, fruits, and cash crops programme in western Nepal. Food Nutr Bull [Internet]. 2000;21:445–53. Available from: http://ovidsp.ovid.com/ovidweb.cgi?T=JS&CSC=Y&NEWS=N&PAGE=fulltext&D=caba5&AN=20001417846 http://bf4dv7zn3u.search.serialssolutions.com.myaccess.library.utoronto.ca/?url_ver=Z39.88-2004&rft_val_fmt=info:ofi/fmt:kev:mtx:journal&rfr_id=info:sid/Ovid:caba5&r

85. Victora C G, Huttly, S R, Fuchs, S C, Olinto, M T. The role of conceptual frameworks in epidemiological analysis: a hierarchical approach. Int J Epidemiol. 1997;26:224–7.

86. Jann B. The Blinder–Oaxaca decomposition for linear regression models. Stata J. 2008;8:453–79.

87. Headey DD, Hoddinott J. Understanding the Rapid Reduction of Undernutrition in Nepal. PLoS One. 2015;10:e0145738.

88. Woodruff BA, Wirth JP, Bailes A, Matji J, Timmer A, Rohner F. Determinants of stunting reduction in Ethiopia 2000 – 2011. Matern Child Nutr. 2017;13.

89. Alderman H, Headey D. The timing of growth faltering has important implications for observational analyses of the underlying determinants of nutrition outcomes. PLoS One. 2018;13:e0195904.

90. Restrepo-Méndez MC, Barros AJ, Black RE, Victora CG. Time trends in socio-economic inequalities in stunting prevalence: analyses of repeated national surveys. Public Health Nutr. 2014;18:2097–104.

91. Sandelowski M. Focus on Qualitative Methods Sample Size in Qualitative. Res Nurs Heal. 1995;18:179–83.

92. Green J, Browne J. Principles of Social Research. Green, J. & Browne J, editor. Maidenhead: Open University Press; 2009.

93. Miles, M.B. & Huberman M. Qualitative Data Analysis: A Sourcebook of New Methods. 2nd ed. Beverly Hills, California: SAGE Publications; 1994.

94. Government of Nepal. Food Act, 2023 (1967) [Internet]. 1967. Available from: http://www.dftqc.gov.np/downloadfile/foodact2023_1334043787_1382615399.pdf

95. The Government of Nepal. The Mother’s Milk Substitutes (Control of Sale and Distrivution) Act [Internet]. Nepal; 1992. Available from: https://extranet.who.int/nutrition/gina/sites/default/files/NPL 1992 Mother%27s Milk Substitues Control of sale and distribution Act 2049.pdf

96. Government of Nepal. The Mother’s Milk Substitutes (Control of Sale and Distribution) Regulation, 1994 (2051) [Internet]. 1994. Available from: http://nnfsp.gov.np/PublicationFiles/0751a21c-4cfd-46ff-bbdf-a7cd5cedcb52.pdf

97. Ministry of Health. Iodized Salt (Production, Sale and Distribution) Act, 2055 (1998) [Internet]. 1998. Available from: www.lawcommission.gov.npwww.lawcommission.gov.np

98. MOHP New ERA and ICF DHS Program. Nepal Demographic and Health Survey 2016. 2017;636.

99. Government of Nepal. Local Self-Governance Act, 2055 (1999) [Internet]. 1999. Available from: http://www.np.undp.org/content/dam/nepal/docs/reports/governance/UNDP_NP_Local Self-Governance Act 1999, MoLJ,HMG.pdf

100. Government of Nepal. National Health Policy. 1991;4–8.

101. Mishra RK. Critics of national health policy 1991 [Internet]. 2015 [cited 2018 Sep 6]. Available from: https://www.slideshare.net/RAVIKANTAMISHRA/critics-of-national-health-policy-1991

102. Dixit H. Plans, Policies and their Implications. Nepal’s Quest for Health [Internet]. 2000 [cited 2018 Sep 5]. p. 202–20. Available from: http://www.hdixit.org.np/quest/CHAP-6.pdf

103. Government of Nepal Ministry of Health and Population. SECOND LONG TERM HEALTH PLAN Perspective Plan for Health Sector Development. 1997; Available from: http://www.mohp.gov.np/app/webroot/upload/files/Second Long Term Health Plan.pdf

104. The World Bank. Education for All Project [Internet]. 2004. Available from: http://documents.worldbank.org/curated/en/265931468775555585/pdf/27890.pdf

105. UNESCO. Education for All 2000-2015: Achievements and Remaining Challenges in Nepal [Internet]. 2015. Available from: www.facebook.com/unescokathmandu

106. Government of Nepal. Nepal: Poverty Reduction Strategy Paper [Internet]. 2003. Available from: https://www.imf.org/external/pubs/ft/scr/2003/cr03305.pdf

107. Ministry of Health. Nepal Health Sector Programme - Implementation Plan [Internet]. 2004. Available from: http://dohs.gov.np/wp-content/uploads/2014/04/NHSP_IP.pdf

108. Government of Nepal. National Nutrition Policy and Strategy. 2004;57.

109. Government of Nepal. National School Health and Nutrition Strategy, Nepal [Internet]. 2006. Available from: http://www.nnfsp.gov.np/PublicationFiles/ce710975-310a-486a-b445-c8ce422674f6.pdf

110. Rai C, Lee SF, Rana HB, Shrestha B kumar. Improving children’s health and education by working together on school health and nutrition ( SHN ) programming in Nepal. 2009;3:0–6.

111. Non-Formal Education Center. Non-Formal Education in Nepal: Status Report 2015-16 [Internet]. 2016. Available from: http://nfec.gov.np/publications/NFE Status Report 2015-16.pdf

112. Ministry of Education. School Sector Reform Plan 2009-2015 [Internet]. 2009. Available from: http://www.moe.gov.np/assets/uploads/files/SSRP_English.pdf

113. Poyck MC, Koirala DBN, Aryal DPN, Sharma CNK. Joint Evaluation of Nepal’s School Sector Reform Plan Programme 2009-16 [Internet]. 2016. Available from: http://finnida.fi/wp-content/uploads/evaluoinnit/hankekohtaiset/School Sector Reform Nepal Final Joint Evaluation 2016 %5B2016,Nepal,2009-2016%5D.pdf

114. Ministry of Health and Population. NEPAL HEALTH SECTOR PROGRAMME-IMPLEMENTATION PLAN II (NHSP-IP 2) [Internet]. 2010. Available from: http://www.nationalplanningcycles.org/sites/default/files/country_docs/Nepal/nhp_nepal.pdf

115. Ministry of Health. National Communication Strategy for Maternal, Newborn and Child Health 2011-16 [Internet]. 2011. Available from: http://dohs.gov.np/wp-content/uploads/chd/SafeMotherhood/National_Communication_Strategy_for_MNCH_2011_2016_EN.pdf

116. Government of Nepal. SANITATION AND HYGIENE MASTER PLAN [Internet]. 2011. Available from: http://washinschoolsmapping.com/wengine/wp-content/uploads/2015/10/Nepal-Government-Sanitation-and-Hygiene-Master-Plan.pdf

117. Himalayan News Service. 38 districts yet to attain ODF status - The Himalayan Times [Internet]. 2016 [cited 2018 Sep 6]. Available from: https://thehimalayantimes.com/nepal/38-districts-yet-attain-odf-status/

118. Kathmandu Tribune. Saptari declared ODF and fully-immunized district - News, sport and opinion from the Kathmandu Tribune’s global edition [Internet]. 2018 [cited 2018 Sep 6]. Available from: https://kathmandutribune.com/saptari-declared-odf-and-fully-immunized-district/

119. Scaling Up Nutrition. Nepal - SUN [Internet]. 2015 [cited 2018 Sep 6]. Available from: http://scalingupnutrition.org/sun-countries/nepal/

120. Government of Nepal National Planning Commission. Multi-sectoral Nutrition Plan: For Accelerating the Reduction of Maternal and Child Under-nutrition in Nepal. 2012;II:1–155. Available from: https://scalingupnutrition.org/wp-content/uploads/2013/03/Nepal_MSNP_2013-2017.pdf (Accessed 20 Jan 2018)

121. National Planning Commission. Multi-sector Nutrition Plan II (2018-2022), Nepal Multi-sector Nutrition Plan [Internet]. 2018. [cited 2018 Sep 6]. Available from: https://publichealthupdate.com/multi-sector-nutrition-plan-ii-2018-2022-nepal/

122. Ministry of Health and Population. Health Sector Strategy for Addressing Maternal Undernutrition [Internet]. 2013. Available from: http://dohs.gov.np/wp-content/uploads/chd/Nutrition/National_Strategy_Maternal_Undernutrition.pdf

123. Ministry of Agricultural Development. Food and Nutrition Security Plan of Action. 2013;1–52.

124. Ministry of Health and Population of Nepal. Strategy for infant and young child feeding: Nepal 2014. Nepal; 2014.

125. USAID. LEARNING FROM NEPAL’S MULTI-SECTORAL NUTRITION PLAN IMPLEMENTATION IN THREE PRIORITY DISTRICTS : “Pathways to Better Nutrition” Study [Internet]. 2014. Available from: https://www.spring-nutrition.org/sites/default/files/publications/briefs/nepal-pbn-brief_web-tagged_1.pdf

126. World Health Organization. EPI Fact Sheet - Nepal [Internet]. 2016. Available from: http://www.searo.who.int/immunization/data/nepal.pdf

127. Government of Nepal Ministry of Health and Population. National Immunization Program [Internet]. 2016 [cited 2018 Aug 21]. Available from: http://www.chd.gov.np/index.php/programs/national-immunization-programme

128. Dahal P, Sharma A, Chitekwe S. A journey to multi-sector nutrition programming in Nepal: evolution, processes and way forward | ENN [Internet]. 2017 [cited 2018 Sep 6]. Available from: https://www.ennonline.net/fex/54/multisectornutritionnepal

129. USAID. Vitamin A Supplements for Children [Internet]. 2012. Available from: www.nfhp.org.np

130. Fiedler JL. The Nepal National Vitamin A Program: prototype to emulate or donor enclave? Health Policy Plan [Internet]. 2000 [cited 2018 Sep 6];15:145–56. Available from: http://www.ncbi.nlm.nih.gov/pubmed/10837037

131. Department of Health Services. Annual Report [Internet]. 2016. Available from: http://dohs.gov.np/wp-content/uploads/2017/06/DoHS_Annual_Report_2072_73.pdf

132. The World Bank. School enrollment, primary (% net) | Data [Internet]. Open Data . 2018 [cited 2018 Sep 6]. Available from: https://data.worldbank.org/indicator/SE.PRM.NENR

133. UNDP. Micro Enterprise Development Programme (MEDEP) [Internet]. 2017. Available from: http://www.np.undp.org/content/dam/nepal/docs/factsheets/medep.pdf

134. Government of Nepal. Micro-Enterprise Development Programme [Internet]. 2008 [cited 2018 Sep 6]. Available from: http://medep.org.np/index.php?page=#

135. UNDP. Funding and delivery | UNDP in Nepal [Internet]. 2018 [cited 2018 Sep 6]. Available from: http://www.np.undp.org/content/nepal/en/home/about-us/funding-and-delivery.html

136. Pokharel RK, Houston R, Harvey P, Bishwakarma R, Adhikari J, Dev Pant K, Gartoulla R. Nepal Nutrition Assessment and Gap Analysis Final Report Nutrition Assessment Team 1 [Internet]. 2009. Available from: https://pdf.usaid.gov/pdf_docs/pnaea792.pdf

137. USAID. Suaahara Project – Good Nutrition - Nepal | ReliefWeb [Internet]. 2016 [cited 2018 Sep 6]. Available from: https://reliefweb.int/report/nepal/suaahara-project-good-nutrition

138. Nutrition International. Micronutrient Initiative Supports Mandatory Flour Fortification in Nepal to Improve Health of the Most Vulnerable [Internet]. 2011 [cited 2018 Sep 6]. Available from: https://www.nutritionintl.org/2011/08/micronutrient-initiative-supports-mandatory-flour-fortification-nepal-improve-health-vulnerable/

139. Food Fortification Initative. Country Profile - Nepal [Internet]. 2012 [cited 2018 Sep 6]. Available from: http://www.ffinetwork.org/country_profiles/country.php?record=149

140. Oshima K, Biradavolu M, Bashyal C, Bhattarai M. Qualitative Study of &quot;Sunaula Hazar Din&quot; Community Action for Nutrition Project Nepal [Internet]. 2017. Available from: www.worldbank.org

141. USAID Nepal. Knowledge-based Integrated Sustainable Agriculture and Nutrition (KISAN) Project. 2017; Available from: https://www.usaid.gov/sites/default/files/documents/1861/SEED - KISAN-FINAL.pdf

142. Devkota B, Van Teijlingen ER. Understanding effects of armed conflict on health outcomes: the case of Nepal. [cited 2018 Mar 21]; Available from: https://conflictandhealth.biomedcentral.com/track/pdf/10.1186/1752-1505-4-20?site=conflictandhealth.biomedcentral.com

143. The World Bank. World Bank Indicator [Internet]. [cited 2018 Aug 29]. Available from: https://data.worldbank.org/indicator

144. The World Bank. World Bank Indicator.

145. Sharma S, Shibani P, Pathak D, Sijapati-Banett B. State of Migration in Nepal [Internet]. Centre for the Study of Labour and Mobility. 2014. 102 p. Available from: http://ceslam.org/docs/publicationManagement/STATE OF MIGRATION IN NEPAL1404964819.pdf

146. Muzzini E, Aparicio G. Urban growth and spatial transition in Nepal: an initial assessment. Directions in Development. 2013. 163 p.

147. University of Oxford. Oxford Poverty and Human Development Initiative (OPHI) Country Briefing : Egypt. 2016;1–10. Available from: www.ophi.org.uk/multidimensional-poverty-index/mpi-country-briefings/

148. National Planning Commission,Government of Nepal OP and HDI. Nepal Multidimensional Poverty Index. 2018;19:1. Available from: http://www.springerlink.com/index/9XJGK6DD7DFWMLW9.pdf

149. Government of Nepal Ministry of Education. Education for all national review report 2001-2015: Nepal. 2015.

150. MoE. Non-Formal Education Policy, 2063 B.S. (2007 A.D.). 2007;1–23.

151. Wright J. Essential Package of Health Services Country Snapshot: Bangladesh. Heal Financ Gov Proj [Internet]. 2015; Available from: https://www.hfgproject.org/essential-package-of-health-services-country-snapshot-bangladesh/

152. Mishra SR, Khanal P, Dhimal M. Nepal’s quest for Universal Health Coverage. J Pharm Pract Community Med. 2016;2:104–6.

153. World Health Organization. Health System in Nepal: Challenges and Strategic Options November 2007. 2007.

154. DHS/MOH, WHO, UNICEF U. Development of Integrated Management of Childhood Illness (IMCI) in Nepal. 2002.

155. Acharya, L.B. & Cleland J. Maternal And Child Health Services In Rural Nepal : Does Access Or Quality Matter More? Health Policy (New York). 2000;15:223–9.

156. Khatri RB, Mishra SR, Khanal V. Female Community Health Volunteers in Community-Based Health Programs of Nepal: Future Perspective. Front Public Heal [Internet]. 2017;5:1–4. Available from: http://journal.frontiersin.org/article/10.3389/fpubh.2017.00181/full

157. Andersen K, Singh A, Shrestha MK, Shah M, Pearson E, Hessini L. Early pregnancy detection by female community health volunteers in Nepal facilitated referral for appropriate reproductive health services. Glob Heal Sci Pract [Internet]. 2013;1:372–81. Available from: http://www.ghspjournal.org/cgi/doi/10.9745/GHSP-D-12-00026

1. Improved sanitation facilities include flush/pour flush toilets connected to piped sewer systems, septic tanks or pit latrines; pit latrines with slabs (including ventilated pit latrines), and composting toilets (WHO GHO) [↑](#footnote-ref-1)
2. Improved water sources include piped water, boreholes or tubewells, protected dug wells, protected springs, and packaged or delivered water (WHO GHO) [↑](#footnote-ref-2)
