# Supplementary material for: Drivers of stunting reduction in Nepal: a country case study
Source: Am J Clin Nutr. 2020 Sep 5;112(Suppl 2):844S–859S. doi: 10.1093/ajcn/nqaa218 (PMC7487432; doi:10.1093/ajcn/nqaa218)
Supplement: nqaa218_Supplemental_Files [file nqaa218_supplemental_files.zip › Nepal_Stunting_Appendices_May62020.docx]

## **Appendices**

**Appendix 1: Systematic literature review of stunting determinants**

Among the basic determinants of stunting in Nepal, our literature review identified living rurally and within the mountain or hill regions of the country (1–7), wealth index (1,4,6–18) and maternal education (8,9,13,17,19–24) to be significant. A total of 7 studies found the prevalence of stunting among children under-5 in Nepal to be higher in rural areas and in the mountain or hill regions compared to more urban locations and the Terai plains. In particular, one study by Gaire et al in 2016 found that children living in the hill region were 1.24 times as likely to be stunted as children in the Terai plains region, and children in the mountain region were 1.52 times as likely to be stunted, though only the mountain region findings were statistically significant (6). In terms of the impact of wealth index on child growth, 11 studies showed that children in the poorest households were more likely to be stunted than children in the richest households, with many of these studies showing the odds to be twice as likely or more (1,4,6,7,10–12,15–18). Corresponding to this, 4 studies found that asset accumulation, which can be used as a proxy for increases in wealth, was linked to improvements in HAZ among children in Nepal. However, despite these findings and the overall reduction in poverty that Nepal has experienced over the last two decades, 3 studies found persistent inequalities across wealth quintiles for stunting decline over the years, with larger reductions seen among the wealthy compared to the poor (3,20,25). Lastly, maternal education and women’s empowerment, particularly as it relates to household agricultural production (26–28), were seen to have an impact on stunting outcomes in Nepal. In total, 10 studies found maternal education to have a positive impact on stunting outcomes (8,9,13,17,19–24). In particular, a 2015 study by Headey and Hoddinott found that the HAZ difference between the child of a mother with no education and one with six years of education was approximately 0.17 standard deviations, and between a child whose mother had no education and one whose mother had completed secondary school, it was 0.34 standard deviations difference (9).

Underlying determinants of stunting in Nepal were food insecurity (12,29–31), infant and young child feeding (10,31–36), access to health services (4,8,13,14,19,37,38), and improved sanitation (8,9,13,14,38). A 2010 cross-sectional study conducted by Osei et al in the Terai region found that 69% of households were food insecure and that the prevalence of stunting was slightly higher among food insecure homes, however this was not found to be statistically significant (12). In contrast to this, however, three studies included in our literature review did find living in food deficit families to be a statistically significant risk factor for stunting in children, with one 2012 study by Paudel et al showing food insecurity to increase the odds of being stunted by 4.26 times compared to children in food secure homes (29–31). Along with food insecurity, infant and young child feeding practices were linked to stunting in seven studies included in the literature review (10,31–36). Specifically, issues around inappropriate exclusive breastfeeding, complementary feeding and a lack of dietary diversity were seen to be risk factors (31). 2014 data compiled by Chaparro et al showed that the greatest increases in stunting in Nepal were taking place between infants 9-18 months of age due to inadequate breastfeeding and complementary feeding and the rise in illness and infection at this age (39). An additional five studies supported this finding of higher rates or worsening in the severity of stunting among older infants in Nepal (12,21,40–42). Access to health services, and particularly maternal and newborn health care, has been linked to a reduction in stunting in Nepal by seven studies (4,8,13,14,19,37,38). A 2015 study using linear probability modeling of Nepal’s Demographic and Health Survey data from 2001 – 2011 by Headey and Hoddinott found that women attending at least four antenatal care visits predicted a 0.09 standard deviation increase in HAZ, and delivery in a hospital was associated with an almost 0.20 standard deviation improvement in HAZ (9). Lastly, improvements in sanitation were shown to have an impact on stunting outcomes in Nepal in five studies (8,9,13,14,38). In one study, community toilet use showed a 0.14 standard deviation improvement in children’s HAZ scores (8). Findings on the impact of access to an improved water source have been more mixed, with two of the five aforementioned studies showing tubewell water sources to have a positive impact on HAZ (9,13), while all five studies showed piped water to have no impact on HAZ (8,9,13,14,38).

The immediate determinants of stunting reduction in Nepal according to our literature review include immunization (6,8,32,43) and maternal nutrition (8,12,17–19,32,44). Overall, four studies found links between immunization for childhood illnesses and HAZ outcomes among children in Nepal (6,8,32,43). In particular, a 2016 study by Cunningham et al found through regression analysis of Nepal’s DHS data from 1996-2011 that there was a relative contribution of 0.18 standard deviations to HAZ among children who received all of their vaccinations (8). Conversely, two studies included in this literature review found an association between infection and illness in Nepalese children and poor growth outcomes (41,45). Maternal nutrition was also linked to child growth outcomes in seven studies. Specifically, two studies found associations between mother’s body mass index and the stunting and/or length-for-age outcomes of their children, while five studies found the intergenerational impact of maternal height on child stunting outcomes to be significant, albeit to varying degrees (8,12,17–19,32,44).

**Appendix 2: Program and policy review**

Following the establishment of a constitutional monarchy and multi-party democracy in 1990, the Government of Nepal launched several initiatives to reform and strengthen healthcare delivery. Chief among these was the new vision for the health system via the 1991 National Health Policy, which focused on providing essential care services to the country’s largely rural population in a more accessible way. Along with this came the Second Long Term Health Plan (1997-2017), which built on the First Long Term Health Plan’s efforts to improve health service delivery by working to address disparities in health outcomes and making quality healthcare more readily available at the community-level.

In 2007, Nepal’s Interim Constitution stated that every citizen had the right to free basic health services provided by the State. Since then, free essential health services have been progressively rolled out, reaching national scale in 2009. All citizens are now eligible to access district hospitals, primary health care centres, health posts and sub-health posts without paying a registration fee. They can also access free outpatient, in-patient and emergency services, and free medication from a list of essential drugs (46,47). Despite the government commitment to health as a human right and an observed increase in service utilization, challenges to the implementation of free essential health services have been ongoing in Nepal. Among these are issues surrounding proximity and distribution of health services across the country, inadequate budgeting for health, drug stock outs, and a lack sufficient human resources for health, particularly in remote and rural areas (46,48)

These initiatives also came alongside longstanding commitments by both the government and external donors to lifesaving health interventions such as the National Immunization Program (1979-present) and the National Vitamin A Supplementation Program, as well as the development of new packages of interventions such as the Community-Based Integrated Management of Neonatal and Childhood Illness (CB-IMNCI) (1997-present), and the National Nutritional Policy and Strategy (2004-present).

In order to achieve its goal of greater access within the health system and the broader provision of essential services, the government began a push in the 1990s for decentralization through two key initiatives. Firstly, the Female Community Health Volunteer (FCHV) Program helped extend the reach of the health system through the training of married women between the ages of 25-45 years to provide health and nutrition information and basic health services within their communities. When it was rolled out nationally in 1992, the FCHV program had approximately 20,000 volunteers. According to the most recent estimates, there are currently more than 50,000 volunteers working across Nepal (49). Secondly, the Local Self Governance Act of 1999 created the legal framework for important decisions to be made at the community level, including those related to health and education.

Along with a focus on basic health services, the Government of Nepal and its development partners placed special focus on improving maternal and newborn health outcomes through overarching programs such as the Safe Motherhood Program (1997-present) (50). From this initiative, subsequent efforts such as the 2005 Safe Delivery Incentive Program (later becoming *Aama*, the Nepali word for “mother”) and the 2006 National Policy on Skilled Birth Attendants were created. While the former works to provide free delivery services and cash incentives to both the institutions providing maternal care and the women accessing it, the latter has worked to drive up the number of skilled birth attendants available to assist in delivery (51,52).

The prioritization of key nutrition-sensitive programs by both government and donors was also noteworthy. Among these include long-term efforts to improve education and literacy through the Education for All Initiative (2000-2015); a focus on pro-poor economic growth and increased access to social and economic resources for the marginalized through the Poverty Reduction Strategy (2002-2007); and locally-driven efforts to improve the water, sanitation and hygiene (WASH) sector via Community-Led Total Sanitation (CLTS) beginning in 2000.

**Appendix 3: Qualitative inquiry results**

### **National Expert Stakeholders**

Among the contextual factors for stunting decline that national expert stakeholders pointed to were the overcoming of political instability and conflict, improvements in education (particularly for women and girls), women’s empowerment, increases in remittances allowing for improvements in standard of living and nutrition, reduction in poverty and urbanization.

Nutrition-specific and –sensitive policies and programs discussed during interviews with national expert stakeholders were ranked based on the number of times they were mentioned or endorsed by the participants. Overall, those most frequently referenced point to the importance of a broad mixture of initiatives that target both specific outcomes such as maternal and newborn health and nutrition, and more broad-reaching efforts, such as those focused on WASH, poverty reduction and education.

Among the underlying causes of stunting reduction, national expert stakeholders pointed to improvements in WASH, an increase in the accessibility of essential health services at the community-level, and critical improvements in food security and overall dietary diversity due to changing agricultural practices.

*“WASH-related policies were also quite useful in reducing infection. Campaigns on ODF (Open Defecation Free) were instrumental in reducing infections among children, thereby improving nutritional status including stunting.”* - Representative from the Ministry of Health and Population (former)

Among the immediate causes of stunting reduction, expert stakeholders outlined a reduction in infections and communicable diseases, increased dietary intake (particularly of macro and micronutrients and improved infant and young child feeding practices), and decreased fertility, especially among the adolescent population.

### **Community Stakeholders**

Community stakeholders, represented by Female Community Health Volunteers and other community health workers, described increases in remittance incomes and expanded access to health services as essential to improvements in child health outcomes. Importantly, the labour migration of men within communities and increased education among women were seen to have knock on effects for women’s empowerment, with more women assuming roles as the head of the household and as decision-makers.

*“...Education, especially among women, has improved over the years and has led to self-understanding about the availability and utilization of health services. Women’s education is a proven and important means of achieving gender equality, the effects of which are felt throughout families and communities if compared with the past.” -* Community Health Worker, Thecho

Related to nutrition-specific and –sensitive policies and programs, community health workers outlined the importance of increased donor and development partner funding leading to an expansion of health service providers, which led to improvements in the ability to address maternal, newborn and child health, as well as nutrition.

For underlying causes, many community health workers described increased access to health services and water, sanitation and hygiene improvements over the study period. For immediate causes, participants among the health workers also pointed to a reduction in child illness and improved dietary intake, including exclusive breastfeeding.

### ***Mothers in Communities***

Among the contextual factors outlined by mothers were improvements in socioeconomic status and a reduction in poverty as well as improvements in education and the empowerment of women to make decisions within their families and communities.

*“…compared to early days, it has improved a lot. We had nothing. We were very much poor. The major source of income was only agriculture. We struggled for minimal day-to-day survival. But now the times have changed. We are now economically strong. Our sons/daughters are abroad. They send us money. We do have our own agricultural land. We consume food for ourselves from there and also sell seasonally.” (Smiling*) - P1: Mother of a child born between 1995-2000.

Among the underlying factors for stunting reduction, mothers pointed to improvements in food security, water and sanitation, and overall access to health services. In particular, women pointed out how the conflict from 1996-2006 and issues of geographic remoteness had an impact on the availability of food. They also recognized how demands on their time had changed over the last 20 years, allowing for more attention to be paid to infant and young child feeding, and particularly to breastfeeding. In terms of health services, local access via community health workers and improvement of government and private health facilities was seen to be important. Several participants also pointed to improvements in roads and transportation services, though many noted progress is still need in this area.

*“The health facilities in the past used to have no medical equipment and professional personnel used to be very far, which is not the case today. Now the transports reach to the door of health institutions. Everything has improved with time. Availability of drugs and other services is noted to be continually provided to every sick patient with no age bar” -* P5: Mother of child born between 1995-2000

Among the immediate causes of stunting reduction mentioned by mothers in the community were improved dietary diversity and overall dietary intake, and decreased incidence of infectious disease, low birth weight and fertility rates, as well as greater spacing between births.
